# Polyaromatic Calixarene Hosts: Calix[4]pyrenes

**DOI:** 10.1021/acs.orglett.4c01850

**Published:** 2024-06-27

**Authors:** Michal Farber, Varun Rawat, Yael Diskin-Posner, Roman Dobrovetsky, Arkadi Vigalok

**Affiliations:** † School of Chemistry, The Raymond and Beverly Sackler Faculty of Exact Science, Tel Aviv University, Tel Aviv 69978, Israel; ‡ Department of Chemical Research Support, Weizmann Institute of Science, Rehovot 7610001, Israel

## Abstract

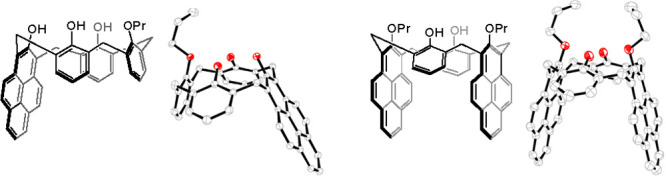

Calixpyrenes, calix[4]arenes incorporating one or two pyrene moieties as a part of their hydrophobic cavities, have been prepared and fully characterized. Distally di-*O*-propoxy diether of the calix dipyrene, which exists in the pinched cone conformation with nearly parallel pyrene moieties, demonstrates strongly enhanced binding of an organic cation (*N*-methylpyridinium) compared with the analogous diethers of the parent calix[4]arene.

Host–guest complexation of small molecules and ions within the hydrophobic cavities of 3D cyclic aromatic compounds has been extensively studied for several decades.^1^ Most of these cyclic hosts are produced by a condensation reaction between an aldehyde or ketone and an electron-rich aromatic molecule. This synthetic strategy provided access to the calixarene and resorcinarenes,^2^ as the early examples of the aromatic hosts, and later produced new families of the cyclic structures, e.g., calixpyrroles,^3^ calixnaphthalenes,^4^ pillararenes,^5^ and prismarenes.^6^ Through the years, calix[4]arenes (from now on, calixarenes) have received arguably the most extensive amount of attention. With its crystal structure established over 40 years ago (as a host–guest complex with toluene),^7^ many modifications of the calixarene structure followed thanks to the straightforward synthetic protocols. Most commonly, these modifications are introduced at the phenolic part of the molecule (lower rim) via the etherification or esterification reactions.^8^ Alternatively, direct aromatic substitution in the position *para*- to the phenolic oxygen (upper rim) was used to introduce various groups (Figure 1a).^9^ These classical approaches to modify the calixarene cavities produced numerous structures with interesting properties for the chemosensory, biological, or materials applications. The structures, however, only possess functional groups as pendants to the calixarene cavity with a high degree of flexibility.

**Figure 1:**
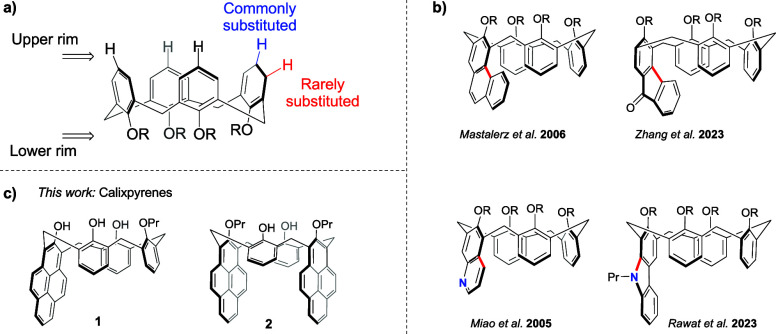
(a) A calix[4]arene molecule with possible substitution sites. (b) Selected examples of calix[4]arenes containing fused aromatic moieties. (c) New calix[4]pyrenes.

Calixarene substitution at the *meta*-position is significantly more challenging and a fairly new area of research.^10^ In their seminal work, Lhoták et al. employed mercury reagents to achieve the selective *direct meta*-functionalization of unsubstituted calixarenes.^11^ Other approaches involved calixarenes premodified in the *para*-position, including stereoselective substitution.^12^ This approach has been used to prepare relatively rare fused calixarene systems, giving various rigid poly(hetero)aromatic calixarene scaffolds, some of which are shown in Figure 1b.^13,14^ Very recently, our group reported the preparation of the first calixarenes incorporating a carbazole motif (Figure 1b, right), giving inherently fluorescent host molecules which demonstrated interesting properties in the host–guest complexation and sensing.^13d^ Interestingly, to our knowledge, no bicyclic, double-fused calixarenes substituted at *both meta*-positions of a single aromatic ring have appeared in the literature. Such a substitution pattern can give access to new families of inherently fluorescent calixarene hosts with expanded polyaromatic cavities with potential applications in fluorescent host–guest sensing.^15^ Here, we present the synthesis and host–guest complexation studies of the first members of the calix[4]pyrene family **1** and **2** (Figure 1c) that incorporate the pyrene moiety, double-fused at the *meta*-positions, as a part of the calixarene cavity.

The synthetic protocol toward the calix monopyrene **1** is shown in Scheme 1a. In short, the previously reported boronic ester **3** was reacted with the commercial dialdehyde **4** under the Suzuki cross-coupling conditions, affording the new calixarene **5** in a 70% yield.^16^ Next, the aldehyde groups were converted to terminal acetylenes via the two-step Corey–Fuchs reaction. The resulting **6** was cyclized to the calix monopyrene **7** under the PtCl_2_-mediated conditions.^17^ The ^1^H NMR spectrum of **7** shows the expected pattern for the pyrene unit at ca. 7.8–8.6 ppm, significantly downfield from diacetylene **6**. Finally, the protecting benzyl groups were removed by 48% HBr giving the calix monopyrene **1**. The solution UV and emission spectra of **7** and **1** show multiple signals common for a pyrene moiety, with strong fluorescence maxima at 392 and 411 nm (Figure 2a). Ultimate confirmation of the proposed structure was obtained from the X-ray crystallographic analysis. The crystal structure of **1** demonstrates the incorporation of a pyrene moiety into a calixarene scaffold (Figure 3a). There are two calixarene molecules in the unit cell with the interpenetrating pyrene moieties (Figure S72). As expected from the resonance structures, the C–C bond distances of the K-region (1.352 (3) and 1.346 (3) Å) are the shortest within the pyrene fragment. The calixarene scaffold adopts a pinched cone conformation with the opposing free phenol units engaging in hydrogen bonding with the etheric oxygen atoms. This arrangement results in a wider upper rim opening for phenol rings, although this is certainly affected by the π-stacking of two pyrene units in the crystal packing.

**Figure 2:**
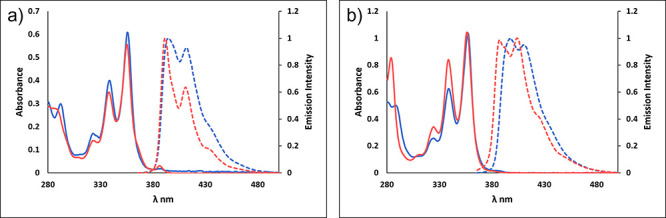
UV–vis (solid line) and emission (dashed line) spectra of (a) calix monopyrenes **7** (blue) and **1** (red) and (b) calix dipyrenes **11** (blue) and **2** (red). The spectra are taken in CHCl_3_–CH_3_CN (9:1) at 10 μM concentration.

**Figure 3:**
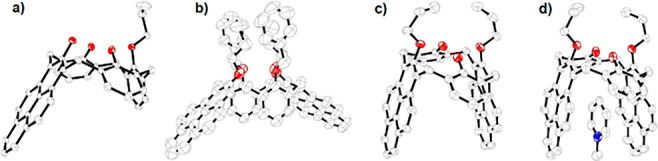
X-ray structures of compounds **1**, **11**, **2**, and host–guest complex **12⊂2**. Hydrogen atoms, solvents, and anions are omitted for clarity.

**Scheme 1:**
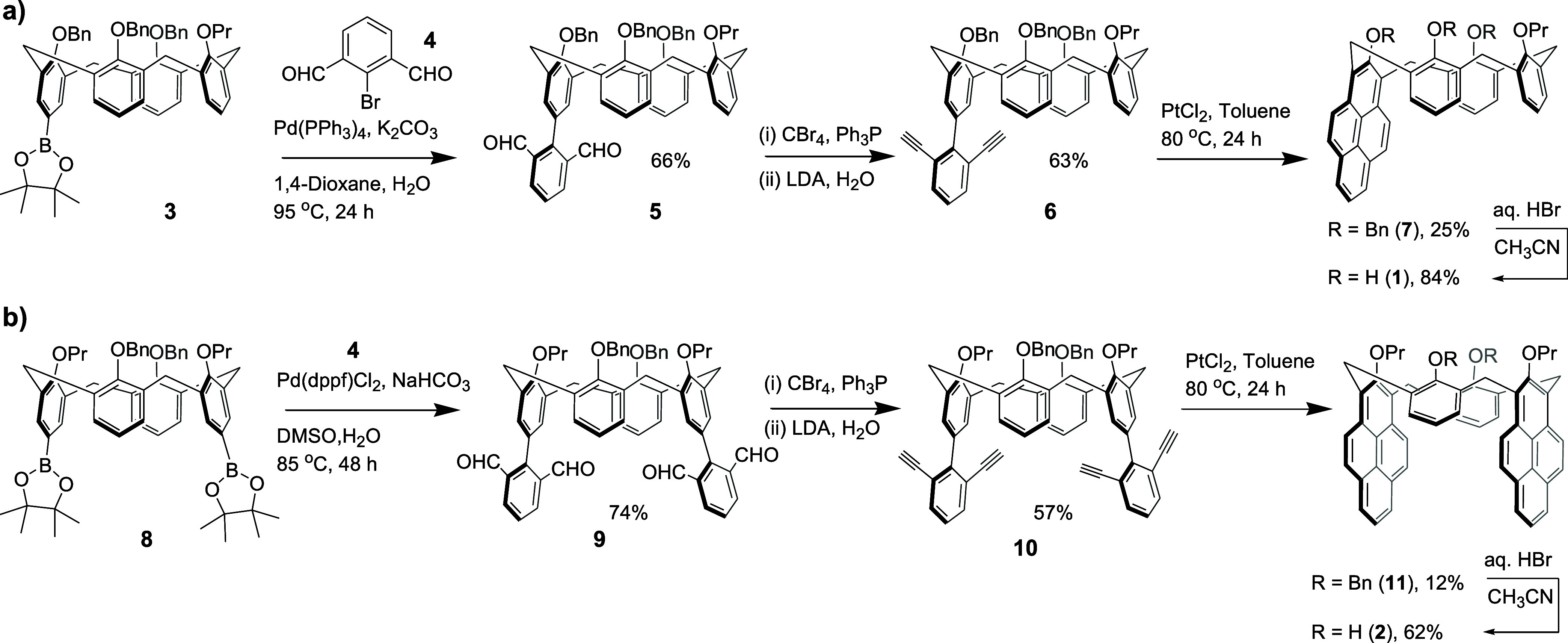
Synthesis of the Calix[4]pyrenes

The calix dipyrene **2** was prepared following the synthetic route similar to the synthesis of **1** (Scheme 1b). The pyrene cyclization step assisted by PtCl_2_ proved to be more challenging than in the case of the calix monopyrene **7**, giving the corresponding dibenzyl ether **11** in only 12% yield. The ^1^H NMR spectra of **11** and **2** showed the pattern expected for a symmetrically substituted calixarene system. Both calix dipyrenes were fluorescent in solution (Figure 2b); however, the deprotected **2** showed weaker absorbance and fluorescence signals. The emission spectra did not show evidence for the excimer formation, suggesting partial aggregation-caused quenching taking place upon moving two pyrene fragments in the nearly parallel positions. Indeed, the X-ray structure of **11** shows the pyrene fragments forming the wide opening of the calixarene cavity in the pinched cone conformation (Figure 3b). The same conformation is apparently present in solution, as the signals of the protons at the unsubstituted aromatic rings in **11** move strongly to high field compared with diacetylene precursor **10** and appear at ca. 5.5 ppm, testifying to their proximity to the pyrene moieties (Figure 4a). This proximity is also evident from the NOESY measurement which shows through space interactions between these protons and the protons of the pyrene K-region (Figure S42). Similar conformational arrangement was also observed for the relevant calixarene tetraethers bearing fused phenanthrene and quinoline units, with the fused rings forming the wide opening of the pinched cone.^18,19^

**Figure 4:**
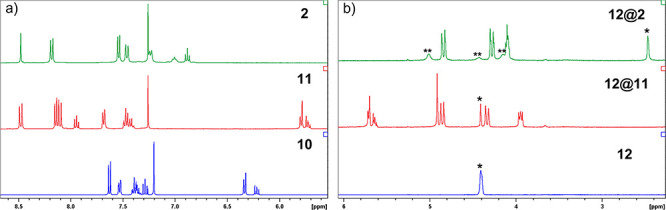
(a) Aromatic region of the ^1^H NMR spectra of compounds **10**, **11**, and **2**. (b) Partial ^1^H NMR spectrum of the guest **12** and its 1:1 mixtures with **11** and **2**. The guest signals are indicated with asterisks.

On the other hand, in **2**, the pinched conformation has two pyrene fragments nearly parallel to each other with the two remaining aromatic rings moving apart. All ^1^H NMR signals for the calixarene rings in **2** appear in the area typical of aromatic protons. This arrangement is also evident from the solid state studies which show significantly smaller upper rim opening between the pyrene fragments vs the remaining phenolic groups (Figure 3c). The intramolecular distance between the pyrene units would be even shorter if not for the *inter*molecular π–π interactions taking place in the crystal lattice, with the structure showing intercalating pyrene units with a distance of ca. 3.5 Å.

The observed parallel arrangement of polycyclic aromatic pyrene moieties capable of strong π–π and cation−π interactions should make calixpyrene **2** predisposed to host electron poor aromatic compounds. In contrast, the tetraether **11** should demonstrate a weaker complexation due to the pyrene units being far apart. To verify this, we studied the host–guest complexation properties of the new calixpyrenes using the *N*-methylpyridinium (NMP) cation **12** (OTf^–^, triflate salt) as a guest. For comparison, we also studied the complexation properties of the alkyl ethers of the parent calix[4]arene under the same conditions (1 mM, CDCl_3_:CD_3_CN, 9:1). Early ^1^H NMR studies of the host–guest complexation between a cation of **12** and tetraalkyl calixarenes **13** provided low binding constants, while the corresponding dialkyl ethers **14** showed somewhat stronger complexation.^20,21^ The stronger complexation properties of the diethers were attributed to the more parallel orientation of the alkyl-substituted phenolic moieties in **14** which likely maximizes π-interactions with the electron-poor aromatic guest.^1a,21^ We recently provided additional evidence for such interactions playing a major role in **12** complexation within the calixarene cavities.^22^ Under our conditions, the NMR titration values were 12.4 ± 0.2 and 71 ± 2.8 M^–1^ for compounds **13** and **14**, respectively, which is similar to the earlier reported data (Table 1).^16^ The ^1^H NMR titration of host **11** with **12** showed roughly 3 times larger *K*_a_ (36.6 ± 6.5 M^–1^) than that for the tetraether **13** as the host but still a relatively weak binding. Remarkably, similar titration of **2** demonstrated a significantly stronger binding with *K*_a_ of (1.37 ± 0.3) × 10^4^ M^–1^—almost 200 times stronger than the corresponding diether **14**. It is also nearly 400 times stronger than the tetraether **11** possessing the “wrong” conformation. Such strong binding is particularly manifested in the large upfield shift of the guest’s signals upon its complexation with **2** (ca. 2 ppm vs free **12**), as compared with minor changes observed for the 1:1 mixture of **12** and **11** (Figure 4b). The ^1^H NMR titration data were further corroborated by fluorescence analysis. Upon the addition of **12**, the fluorescence of **2** was affected dramatically. The titration of **2** with **12** gave the binding constant *K*_a_ = (7.1 ± 1.0) × 10^4^ M^–1^, the same order of magnitude as the constant obtained from the ^1^H NMR titration experiments. It must be noted that the NMR analysis was performed at a 100 times higher concentration than the fluorescence measurements (1 mM vs 10 μM) and thus more susceptible to the error associated with the ion aggregation in nonpolar media.^23^

**Table 1:**
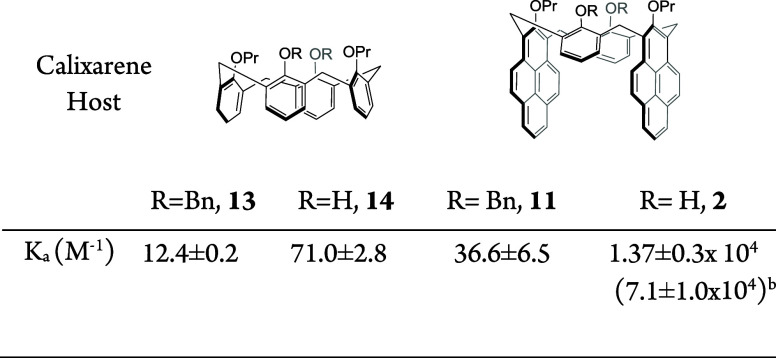
Comparative Binding of Cation **12** with Calixarene Hosts^a^

a ^1^H NMR experiments were performed in a CDCl_3_–CD_3_CN (9:1) mixture.

b From the fluorescence measurements.

We were able to obtain a single crystal of host–guest complex **12**⊂**2**. The crystal structure of this complex (Figure 3d) shows that the whole *N*-methylpyridinium cation is fully immersed in the calixpyrene cavity, with the methyl group pointing toward the opening. The pyridine ring is located at roughly equal distances of ca. 3.5 Å from the pyrene fragments indicating strong π-stacking interactions. The wider opening between the two phenolic fragments is barely affected upon the complexation of **12** (9.949 Å in **2** vs 10.099 Å in **12**⊂**2**), suggesting that no significant conformational changes are necessary to accommodate the pyridinium cation.

Although it is challenging to assess weak interactions using quantum mechanical theoretical methods, a comparison between interactions of a similar nature could still be made. Therefore, to obtain theoretical support for the experimental differences in guest–host interactions in this study, preliminary density functional theory (DFT) gas phase calculations at the BP86-D3(BJ)/def2-SVP level of theory were performed.^24^

Two reactions were calculated to compare the guest–host interactions among **2**, **11**, **14**, and **12**. The exchange of the guest **12** between **11** and **14** was computed. Consequently, the reaction between **11** and **12**⊂**14** leading to **12**⊂**11** and **14** is almost thermoneutral with a Gibbs free energy of −3.1 kcal mol^–1^ (reaction **a**, Figure 5). In contrast, the reaction between **2** and **12**⊂**14** leading to **12**⊂**2** and **14** is significantly more exergonic with a Gibbs free energy of −15.6 kcal mol^–1^ (reaction **b**, Figure 5).

**Figure 5:**
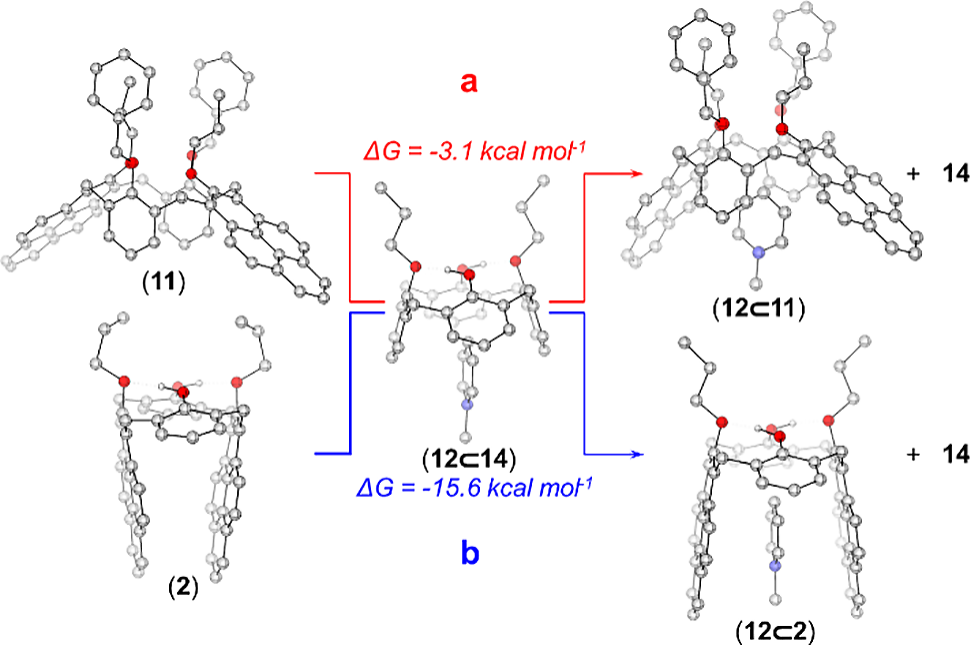
DFT-computed guest, **12**, exchange reactions between **14**, **11**, and **2**.

In summary, we reported the synthesis and complete characterization of the first members of the polyaromatic calix[4]pyrene family. The new compounds show strong fluorescence in solution and demonstrate the enhanced binding of a small organic cation (*N*-methylpyridinium) within the calixarene cavity.

## Data Availability

The data underlying this study are available in the published article and its Supporting Information.

## Supporting Information Available

Complete experimental details (PDF)
xyz files of **2**, **11**, **12** in **2**, **12** in **11**, **12** in **14**, and **14** (ZIP)

## References

[ref1] aGutsche C. D. Calixarenes Revisited; The Royal Society of Chemistry: Cambridge, U.K., 1998.

[ref2] aGutsche C. D. Calixarenes. Acc. Chem. Res. 1983, 16, 161–170. 10.1021/ar00089a003.

[ref3] Kim D. S.; Sessler J. L. Calix[4]pyrroles: Versatile Molecular Containers with Ion Transport, Recognition, and Molecular Switching Functions. Chem. Soc. Rev. 2015, 44, 532–546. 10.1039/C4CS00157E. 24984014

[ref4] Ogoshi T.; Yamagishi T. Pillararenes: Versatile Synthetic Receptors for Supramolecular Chemistry. Eur. J. Org. Chem. 2013, 2013, 2961–2975. 10.1002/ejoc.201300079.

[ref5] Georghiou P. E.; Ashram M.; Li Z.; Chaulk S. G. Syntheses of Calix[4]naphthalenes Derived from 1-Naphthol. J. Org. Chem. 1995, 60, 7284–7289. 10.1021/jo00127a038.

[ref6] Della Sala P.; Del Regno R.; Talotta C.; Capobianco A.; Hickey N.; Geremia S.; De Rosa M.; Spinella A.; Soriente A.; Neri P.; Gaeta C. Prismarenes: A New Class of Macrocyclic Hosts Obtained by Templation in a Thermodynamically Controlled Synthesis. J. Am. Chem. Soc. 2020, 142, 1752–1756. 10.1021/jacs.9b12216. 31898458 PMC7993634

[ref7] Andreetti G. D.; Ungaro R.; Pochini A. Crystal and Molecular Structure of Cyclo{quater[(5-t-butyl-2-hydroxy-1,3-phenylene)methylenel) Toluene (1:1) Clathrate. J.C.S. Chem. Comm. 1979, 1005–1007. 10.1039/c39790001005.

[ref8] aJose P.; Menon S. Lower-Rim Substituted Calixarenes and Their Applications. Bioinorg. Chem. Appl. 2007, 2007, 1 10.1155/2007/65815. PMC1885865 17611612

[ref9] aYesypenko O. A.; Trybrat O. O.; Karpichev Y. A.; Kalchenko V. I. Regioselective Functionalization of the para-Positions at the Calix[4]arene Upper Rim. Curr. Org. Chem. 2023, 27, 510–525. 10.2174/1385272827666230524120812.

[ref10] Lhoták P. Direct Meta Substitution of Calix[4]arenes. Org. Biomol. Chem. 2022, 20, 7377–7390. 10.1039/D2OB01437H. 36083220

[ref11] Slavik P.; Dudic M.; Flidrova K.; Sykora J.; Cisarova I.; Böhm S.; Lhoták P. Unprecedented Meta-Substitution of Calixarenes: Direct Way to Inherently Chiral Derivatives. Org. Lett. 2012, 14, 3628–3631. 10.1021/ol301420t. 22758402

[ref12] Hodson L.; Visagie K. J.; Smith M.-P.; Loots L.; Kuter D.; Snayer T. M.; Arnott G. E. Facile synthesis of a C4-symmetrical inherently chiral calix[4]arene. Chem. Commun. 2021, 57, 11045–11048. 10.1039/D1CC04607A. 34617530

[ref13] aMastalerz M.; Hüggenberg W.; Dyker G. Photochemistry of Styrylcalix[4]arenes. Eur. J. Org. Chem. 2006, 2006, 3977–3987. 10.1002/ejoc.200600265.

[ref14] aTlustý M.; Dvořáková H.; Čejka J.; Kohout M.; Lhoták P. Regioselective Formation of the Quinazoline Moiety on the Upper Rim of Calix[4]arene as a Route to Inherently Chiral Systems. New J. Chem. 2020, 44, 6490–6500. 10.1039/D0NJ01035A.

[ref15] Kumar R.; Sharma A.; Singh H.; Suating P.; Kim H. S.; Sunwoo K.; Shim I.; Gibb B. C.; Kim J. S. Revisiting Fluorescent Calixarenes: From Molecular Sensors to Smart Materials. Chem. Rev. 2019, 119, 9657–9721. 10.1021/acs.chemrev.8b00605. 31306015

[ref16] See the Supporting Information for details.

[ref17] Mamane V.; Hannen P.; Fürstner A. Synthesis of Phenanthrenes and Polycyclic Heteroarenes by Transition-Metal Catalyzed Cycloisomerization Reactions. Chem. Eur. J. 2004, 10, 4556–4575. 10.1002/chem.200400220. 15378635

[ref18] Hüggenberg W.; Seper A.; Oppel I. M.; Dyker G. Multifold Photocyclization Reactions of Styrylcalix[4]arenes. Eur. J. Org. Chem. 2010, 2010, 6786–6797. 10.1002/ejoc.201001108.

[ref19] Tlustý M.; Eigner V.; Dvořáková H.; Lhoták P. The Formation of Inherently Chiral Calix[4]quinolines by Doebner–Miller Reaction of Aldehydes and Aminocalixarenes. Molecules 2022, 27, 8545 10.3390/molecules27238545. 36500638 PMC9736694

[ref20] aAraki K.; Shimizu H.; Shinkai S. Cation-π Interactions in Calix[4]arene-based Host Molecules. What Kind of Cavity-shape ls Favored for the Cation-binding?. Chem. Lett. 1993, 22, 205–208. 10.1246/cl.1993.205.

[ref21] aOrda-Zgadzaj M.; Wendel V.; Fehlinger M.; Ziemer B.; Abraham W. Inclusion of Organic Cations by Calix[4]arenes Bearing Cyclohepta-2,4,6-trienyl Substituents. Eur. J. Org. Chem. 2001, 2001, 1549–1561. 10.1002/1099-0690(200104)2001:8<1549::AID-EJOC1549>3.0.CO;2-X.

[ref22] aRawat V.; Vigalok A. Electronic Tuning of Host-Guest Interactions within the Cavities of Fluorophore-Appended Calix[4]arenes. Molecules 2022, 27, 5689 10.3390/molecules27175689. 36080456 PMC9457996

[ref23] Gibson H. W.; Jones J. W.; Zakharov L. N.; Rheingold A. L.; Slebodnick C. Complexation Equilibria Involving Salts in Non-Aqueous Solvents: Ion Pairing and Activity Considerations. Chem. Eur. J. 2011, 17, 3192–3206. 10.1002/chem.201002522. 21308807

[ref24] aPerdew J. P. Density-Functional Approximation for the Correlation Energy of the Inhomogeneous Electron Gas. Phys. Rev. B 1986, 33, 8822–8824. 10.1103/PhysRevB.33.8822. 9938299

