# Supplementary material for: Polyaromatic Calixarene Hosts: Calix[4]pyrenes
Source: Org Lett. 2024 Jun 27;26(27):5731–5. doi: 10.1021/acs.orglett.4c01850 (PMC11249777; doi:10.1021/acs.orglett.4c01850)
Supplement: Supplementary file 1 — ol4c01850_si_001.pdf [file ol4c01850_si_001.pdf]

Polyaromatic Calixarene Hosts: Calix[4]pyrenes

Michal Farber,[1] Varun Rawat,[1] Yael Diskin-Posner,[2] Roman Dobrovetsky[1*] and Arkadi Vigalok[1*]

[1-] School of Chemistry, The Raymond and Beverly Sackler Faculty of Exact Science, Tel Aviv University, Tel Aviv 69978, Israel. Email: avigal@tauex.tau.ac.il. [2-] Department of Chemical Research Support, Weizmann Institute of Science, Rehovot 7610001, Israel.

Electronic Supporting Information

Contents	Page
1. General Information	S2
2. Experimental Section	S3-S9
3. NMR and MS Spectra	S10-S38
4. Absorption and Emissions Spectra	S39-S40
5. Host-guest binding experiments	S41-S45
6. X-ray data collection and structure refinement	S46-S52
7. References	S53

1. General Information

General. The synthetic manipulations involving air-sensitive compounds were performed in a nitrogen-filled Vigor glove box. All dry solvents were degassed and stored under high-purity nitrogen and activated 4Å molecular sieves. Commercially available reagents (Aldrich, Strem, and Acros) were used as received. Heating was performed using an oil bath with a temperature-controlled internal heater. The NMR spectra were recorded on Bruker Avance 400MHz spectrometer. ^{1}H and $^{13}C\{^{1}H\}$ NMR signals are reported in δ ppm downfield from TMS. Unless stated otherwise, all measurements were performed at 22°C in $CDCl_3$. CombiFlash® NextGen 300+ with silica-filled columns was used for chromatographic purifications unless stated otherwise. X-ray diffraction data for the reported compounds were collected on a Rigaku Oxford Diffraction machine and solved using CrysAlis^{Pro} software. ESI-MS analyses were done on a Waters Acquity UPLC Xevo TQD system, and the High-Resolution Mass Spectrometry data was recorded on a Waters Acquity UPLC Xevo G2-XS QTof instrument. UV and Fluorescence spectra were recorded on Vernier fluorescence/UV-Vis spectrophotometer and Hitachi F-2710 fluorescence spectrophotometer.

Association constant (K_a, M^{-1}) values for the complexes between the *N*-Methyl pyridinium triflate (**12**) and compounds **2**, **11**, **13** and **14** were determined by the ^{1}H NMR titration method in a 9:1 $CDCl_3$:CD_3CN mixture. The host concentration was kept constant at 1 mM while the guest concentrations varied. The constants were calculated using Excel Solver software from equation[1,2] (I):

(I) $$\Delta\delta = \frac{\delta_{\Delta HG}}{2[H]_0}\left(\left([G]_0 + [H]_0 + \frac{1}{K}\right) - \sqrt{\left([G]_0 + [H]_0 + \frac{1}{K}\right)^2 - 4[G]_0[H]_0}\right)$$

Fitting the data using online application at http://supramolecular.org/apps/ gave the same results.

Association constant (K_a, M^{-1}) values for the formation of the complexes between the *N*-Methyl pyridinium triflate (**12**) and **2** were additionally determined by fluorescence titration experiments in a 9:1 $CHCl_3$:CH_3CN mixture. ΔF_{obs} (The initial fluorescence intensity minus the measured fluorescence intensity) against the concentration of the quencher [**12**] was plotted, and the association constant was obtained from a non-linear regression fitting using Excel Solver software and equation[1,2] (II):

(II) $$\Delta\delta = \frac{\delta_{\Delta HG}}{2}\left(\left([G]_0 + [H]_0 + \frac{1}{K}\right) - \sqrt{\left([G]_0 + [H]_0 + \frac{1}{K}\right)^2 - 4[G]_0[H]_0}\right)$$

Fitting the data using online application at http://supramolecular.org/apps/ gave the same results.

Compounds **I**[3], **II** and **IV**[4], **12**[5] and **14**[6] were reported previously in the literature.

DFT calculations were performed using Gaussian 09.[7] Geometry optimization of all the molecules, intermediates, and the transition state were carried out using the BP86-D3(BJ)/def2-SVP basis sets.[8] Thermal energy corrections were extracted from the results of frequency analysis performed at the same level of theory. Frequency analysis of all the molecules and intermediates contained no imaginary frequency showing that these are energy minima.

2. Synthesis of compounds 1-13

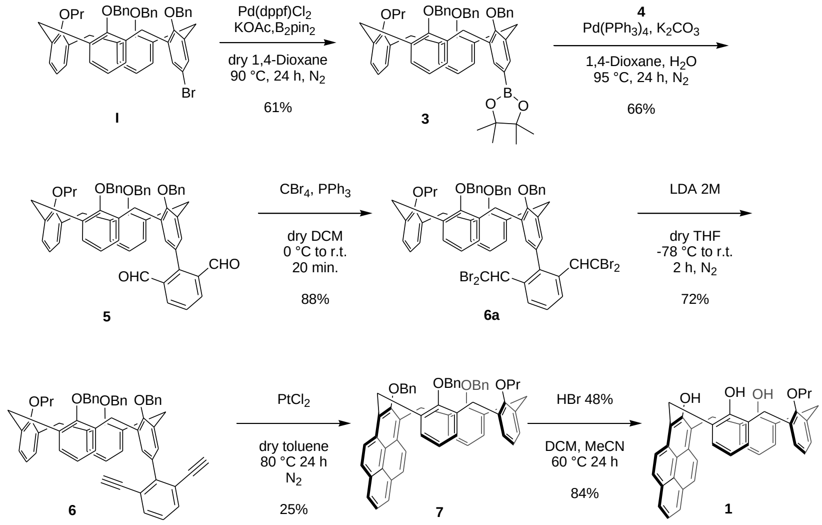

Scheme S1. Synthesis of compound **1**.

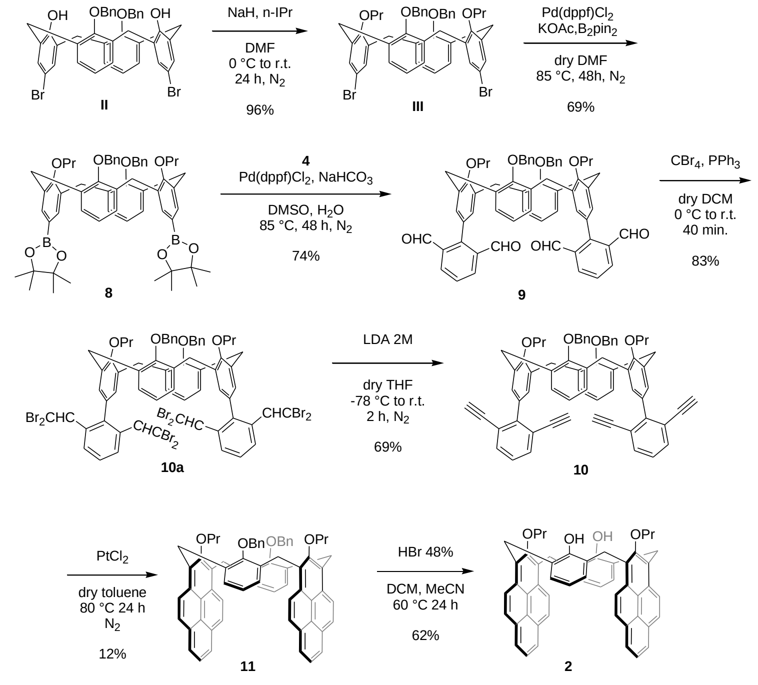

Scheme S2. Synthesis of compound **2**.

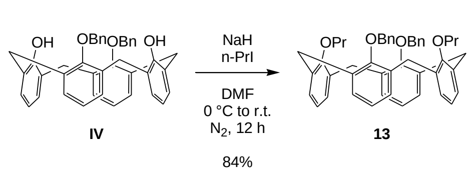

Scheme S3. Synthesis of compound **13.**

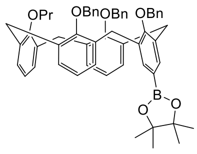

3

Compound 3: Compound **3** synthesis is based on a reported literature method.[3] A mixture of compound **I**[3] (1.9 g, 2.3 mmol), Pd(dppf)Cl_2 (84 mg, 0.12 mmol), K_2CO_3 (0.25 g, 2.5 mmol), dry 1,4-Dioxane (20 mL) and Bis(pinacolato)diboron (0.71 g, 2.8 mmol) was stirred under nitrogen atmosphere at 90 °C for 24 hours. After cooling the reaction to room temperature, the solvent was evaporated under vacuum, and the residue was dissolved in EtOAc and washed with water. The organic phase was dried over $MgSO_4$, filtered, and concentrated to dryness. The crude product was purified using Combi*Flash* ® (CH_2Cl_2-hexane, 1:1) to give white solid **3**. Yield: 61% (1.2 g). ^{1}H NMR (400 MHz, $CDCl_3$) 7.45-7.32 (m, 12H), 7.20-7.16 (m, 1H), 7.09-7.00 (m, 6H), 6.89 (t, *J* = 7.4 Hz, 1H), 6.30-6.26 (m, 2H), 6.16 (d, *J* = 7.6 Hz, 4H), 5.02 (s, 2H), 4.74 (s, 4H), 4.48 (d, *J* = 13.4 Hz, 2H), 4.15 (d, *J* = 13.4 Hz, 2H), 4.04-4.00 (m, 2H), 3.12 (d, *J* = 13.5 Hz, 2H), 2.97 (d, *J* = 13.6 Hz, 2H), 1.92-1.82 (m, 2H), 1.39 (s, 12H), 0.72 (t, *J* = 7.5 Hz, 3H). ^{13}C{^{1}H} NMR (100 MHz, $CDCl_3$) 159.1, 157.7, 154.7, 137.8, 137.7, 136.9, 136.6, 135.6, 133.49, 133.47, 130.1, 128.9, 128.8, 128.3, 127.9, 127.8, 127.7, 127.6, 127.58, 127.53, 122.4, 121.9, 83.6, 77.2, 76.4, 75.4, 31.2, 31.0, 25.0, 23.1, 9.8. HRMS (TOF MS ES+) m/z: $[M+Na]^+$ calcd for $C_{58}H_{59}BO_6Na$ 885.4302, found 885.4319.

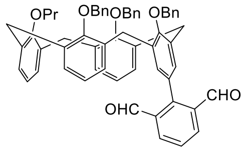

5

Compound 5: Compound **5** synthesis is based on a reported literature method.[9] A mixture of compound **3** (500 mg, 0.58 mmol), 2-Bromoisophthalaldehyde (**4**) (284 mg, 1.33 mmol), K_2CO_3 (240 mg, 1.74 mmol), 1,4-Dioxane (7 mL) and water (1 mL) was degassed with N_2 for 20 minutes. $Pd(PPh_3)_4$ (12 mg, 0.01 mmol) was added, and the reaction was stirred under a nitrogen atmosphere at 95 °C for 24 hours. After evaporating the solvents, water was added, and the aqueous solution was extracted with DCM (x3). The organic phases were combined, washed with brine solution, dried over $MgSO_4$, filtered and concentrated under vacuum. The crude product was purified using Combi*Flash*® (CH_2Cl_2-hexane, 1:1) to give a white-yellow solid **5**. Yield: 66% (331 mg). ^{1}H NMR (400 MHz, $CDCl_3$) 9.59 (s, 1H), 8.82 (s, 1H), 8.10 (d, *J* = 7.7 Hz, 2H), 7.51 (t, *J* = 7.7 Hz, 1H), 7.42-7.28 (m, 11H), 7.25-7.21 (m, 2H), 7.16-7.14 (m, 2H), 6.84 (dd, *J* = 7.4, 1.7 Hz, 2H), 6.70 (t, *J* = 7.4 Hz, 2H), 6.63 (d, *J* = 7.5 Hz, 4H), 6.46 (s, 2H), 6.42 (t, *J* = 7.5 Hz, 1H), 5.23 and 5.15 (ABq, *J* = 11.7 Hz, 4H), 4.87 (s, 2H), 4.41 (d, *J* = 13.2 Hz, 2H), 4.14 (d, *J* = 13.3 Hz, 2H), 3.82-3.78 (m, 2H), 3.16 (d, *J* = 13.3 Hz, 2H), 2.80 (d, *J* = 13.4 Hz, 2H), 1.92-1.84 (m, 2H), 0.95 (t, *J* = 7.5 Hz, 3H). ^{13}C{^{1}H} NMR (100 MHz, $CDCl_3$) 191.7, 191.2, 156.1, 155.4, 155.1, 148.3, 137.7, 136.7, 136.1, 135.34, 135.33, 134.7, 134.5, 134.2, 132.5, 132.0, 131.2, 130.1, 129.9, 129.1, 128.3, 128.2, 128.1, 128.0, 127.6, 125.4, 122.8, 122.3, 77.2, 76.8, 76.2, 31.4, 31.3, 23.4, 10.4. HRMS (TOF MS ES+) m/z: $[M+Na]^+$ calcd for $C_{60}H_{52}O_6Na$ 891.3662, found 891.3652.

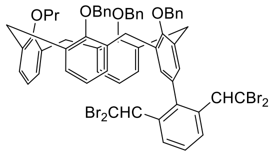

6a

Compound 6a: Triphenylphosphine (1.2 g, 4.72 mmol) was dissolved in dry DCM (10 mL), and the solution was cooled to 0 °C. Carbon tetrabromide (783 mg, 2.36 mmol) was added, and after the mixture became yellow (about 1 minute of stirring), compound **5** (517 mg, 0.59 mmol) in dry DCM (6 mL) at 0 °C was added dropwise. The mixture was left stirring at room temperature for 20 minutes (monitoring with TLC, CH_2Cl_2-hexane, 1:1). Then water was added, and the product was extracted with DCM. The combined organic phases were dried over Na_2SO_4, filtered, and the solvent was reduced under vacuum. The crude product was purified using Combi*Flash*® (CH_2Cl_2-hexane, 1:1) to give white solid **6a**. Yield: 88% (615 mg). ^{1}H NMR (400 MHz, $CDCl_3$) 7.75 (d, *J* = 7.8 Hz, 2H), 7.54-7.52 (m, 4H), 7.45-7.35 (m, 7H), 7.32 (br s, 1H), 7.25-7.21 (m, 1H), 7.18 (t, *J* = 7.0 Hz, 2H), 7.08-7.06 (m, 5H), 6.97 (t, *J* = 7.4 Hz, 1H), 6.81 (s, 2H), 6.36 (t, *J* = 7.6 Hz, 2H), 6.20-6.15 (m, 4H), 5.04 (s, 2H), 4.74 (s, 4H), 4.55 (d, *J* = 13.4 Hz, 2H), 4.20 (d, *J* = 13.4 Hz, 2H), 4.13-4.09 (m, 2H), 3.17 (d, *J* = 13.5 Hz, 2H), 2.96 (d, *J* = 13.5 Hz, 2H), 2.02-1.93 (m, 2H), 0.78 (t, *J* = 7.5 Hz, 3H). $^{13}C\{^1H\}$ NMR (100 MHz, $CDCl_3$) 157.9, 155.4, 154.7, 141.0, 138.0, 137.9, 137.2, 137.0, 136.9, 135.4, 135.3, 133.5, 132.8, 131.5, 130.6, 129.8, 129.0, 128.6, 128.4, 128.0, 127.8, 127.7, 127.6, 127.5, 126.9, 122.9, 121.9, 90.9, 90.4, 77.4, 77.3, 75.4, 31.2, 31.0, 23.3, 9.7. HRMS (TOF MS ES+) m/z: $[M+Na]^+$ calcd for $C_{62}H_{52}Br_4O_4Na$ 1203.0456, found 1203.0485.

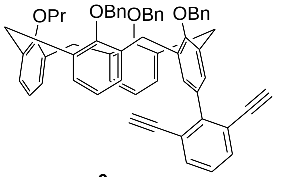

6

Compound 6: Compound **6a** (259 mg, 0.22 mmol) was dissolved in dry THF (5 mL) and cooled to -78 °C under a nitrogen atmosphere. Lithium diisopropylamide (2M in THF, 0.66 mL, 1.32 mmol) was added dropwise, and the solution was stirred at -78 °C for 1 hour. After stirring for another hour at room temperature, the reaction was quenched with HCl 1M and extracted with DCM (x3). The organic phases were combined and washed with water and brine, dried over $MgSO_4$, filtered, and concentrated under reduced pressure. The crude product was purified using Combi*Flash*® (CH_2Cl_2-hexane, 1:2) to give a white solid **6**. Yield: 72% (137 mg). ^{1}H NMR (400 MHz, $CDCl_3$) 7.66 (d, *J* = 7.7 Hz, 2H), 7.56 (m, 4H), 7.48-7.39 (m, 6H), 7.31 (t, *J* = 7.8 Hz, 1H), 7.29-7.24 (m, 1H), 7.18-7.15 (m, 4H), 7.13 (s, 2H), 7.09-7.05 (m, 2H), 6.99 (t, *J* = 7.4 Hz, 1H), 6.38 (dd, *J* = 7.6, 1.6 Hz, 2H), 6.33 (t, *J* = 7.5 Hz, 2H), 6.24 (dd, *J* = 7.4, 1.5 Hz, 2H), 5.09 (s, 2H), 4.82 (s, 4H), 4.58 (d, *J* = 13.4 Hz, 2H), 4.28 (d, *J* = 13.3 Hz, 2H), 4.13-4.09 (m, 2H), 3.21 (d, *J* = 13.5 Hz, 2H), 3.07-3.02 (m, 4H), 2.06-1.97 (m, 2H), 0.82 (t, *J* = 7.5 Hz, 3H). $^{13}C\{^1H\}$ NMR (100 MHz, $CDCl_3$) 157.8, 155.4, 154.8, 148.2, 138.0, 137.6, 137.1, 136.6, 133.6, 133.5, 133.3, 132.7, 130.5, 130.1, 129.0, 128.7, 128.4, 128.2, 128.0, 127.65, 127.62, 127.5, 126.8, 122.6, 122.4, 121.9, 82.7, 80.6, 77.3, 76.4, 75.6, 31.3, 31.1, 23.3, 9.8. HRMS (TOF MS ES+) m/z: $[M+Na]^+$ calcd for $C_{62}H_{52}O_4Na$ 883.3763, found 883.3764.

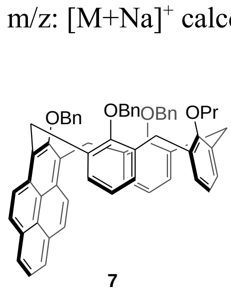

7

Compound 7: Compound **6** (242 mg, 0.28 mmol) was dissolved in dry toluene (12 mL) under a nitrogen atmosphere. Platinum(II) chloride (7.4 mg, 0.028 mmol) was added, and the solution was stirred at 80 °C for 24 hours. After completion of the reaction was determined by TLC (CH_2Cl_2-hexane, 1:2), the solution was concentrated under reduced pressure, and the crude product was purified using Combi*Flash*® (CH_2Cl_2-hexane, 1:1) to give white solid **7**. Yield: 25% (61 mg). ^{1}H NMR (400MHz, CD_2Cl_2) 8.43 (d, *J* = 9.3 Hz, 2H), 8.19 (d, *J* = 7.6 Hz, 2H), 8.12 (d, *J* = 9.3 Hz, 2H), 7.99 (t, *J* = 7.6 Hz, 1H), 7.60-7.39 (m, 10H), 7.19-7.13 (m, 3H), 7.00-6.91 (m, 5H), 6.07 (d, *J* = 7.4 Hz, 2H), 5.97 (t, *J* =

7.6 Hz, 2H), 5.76 (d, J = 7.5 Hz, 2H), 5.22 (s, 2H), 4.90 and 4.81 (ABq, J = 11.0 Hz, 4H), 4.64 (d, J = 14.3 Hz, 2H), 4.54 (d, J = 13.2 Hz, 2H), 4.19 (d, J = 14.2 Hz, 2H), 4.10-4.06 (m, 2H), 3.15 (d, J = 13.3 Hz, 2H), 2.13-2.03 (m, 2H), 0.82 (t, J = 7.5 Hz, 3H). $^{13}C\{^1H\}$ NMR (100 MHz, CD_2Cl_2) 157.7, 154.8, 154.1, 138.0, 137.4, 137.2, 133.5, 133.2, 132.8, 130.8, 130.4, 129.2, 128.86, 128.81, 128.4, 127.9, 127.8, 127.6, 127.4, 127.0, 126.9, 125.3, 125.2, 124.4, 124.0, 122.2, 122.1, 121.9, 77.3, 76.7, 76.3, 31.1, 24.8, 23.2, 9.5. HRMS (TOF MS ES+) m/z: $[M+Na]^+$ calcd for $C_{62}H_{52}O_4Na$ 883.3763, found 883.3765.

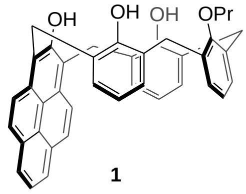

1

Compound 1: Compound **7** (40 mg, 0.05 mmol) was dissolved in DCM (1 mL), and then MeCN (1 mL) and HBr 48% (1 mL) were added. The mixture was stirred at 60 °C for 24 hours. After completion of the reaction was determined by TLC (CH_2Cl_2-hexane, 1:1), The reaction was neutralized with $NaHCO_3$ conc. solution and extraction with DCM was repeated several times. The organic phase was washed with water and brine, filtered, and concentrated under vacuum. The crude product was purified using Combi*Flash*® (CH_2Cl_2-hexane, 1:1) to give white solid **1**. Yield: 84% (23 mg). ^1H NMR (400 MHz, $CDCl_3$) 10.72 (s, 1H), 9.69 (s, 2H), 8.57 (d, J = 9.4 Hz, 2H), 8.01 (d, J = 9.3 Hz, 2H), 7.90 (d, J = 7.6 Hz, 2H), 7.67 (t, J = 7.6 Hz, 1H), 7.51 (dd, J = 7.7, 1.4 Hz, 2H), 7.06 (dd, J = 7.5, 1.4 Hz, 2H), 6.98 (d, J = 7.6 Hz, 2H), 6.73-6.68 (m, 3H), 4.69-4.61 (m, 4H), 4.43 (d, J = 13.0 Hz, 2H), 4.17 (t, J = 6.8 Hz, 2H), 3.49 (d, J = 13.0 Hz, 2H), 2.28-2.23 (m, 2H), 1.36 (t, J = 7.4 Hz, 3H). $^{13}C\{^1H\}$ NMR (100 MHz, $CDCl_3$) 151.43, 151.42, 148.7, 133.9, 129.9, 129.5, 129.3, 129.0, 128.6, 128.4, 127.6, 127.4, 126.0, 125.1, 124.9, 124.8, 123.3, 122.8, 121.6, 120.7, 79.1, 31.6, 26.3, 23.4, 10.8. HRMS (TOF MS ES+) m/z: $[M+Na]^+$ calcd for $C_{41}H_{34}O_4Na$ 613.2355, found 613.2357.

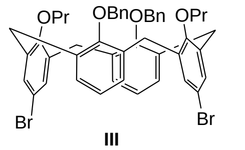

III

Compound III: Compound **III** synthesis is based on a reported literature method.[4] Compound **II** (4.6 g, 6.0 mmol) was dissolved in DMF (150 mL), stirred at 0 °C under N_2, and NaH (1.2 g, 30.0 mmol) was added in batches. After stirring for 1 hour at room temperature, n-propyl iodide (3 mL, 30 mmol) was added, and the reaction was left stirring for an additional 24 hours. The reaction was monitored with TLC (CH_2Cl_2-hexane, 1:3). After all the starting material was consumed, the mixture was cooled to 0 °C and quenched with HCl 1M solution (100 mL). The precipitate was stirred for 15 minutes at 0 °C and collected by vacuum filtration. The filter cake was washed with water and MeOH and dried under a vacuum to obtain a white solid **III**. Yield: 96% (4.9 g). ^1H NMR (400 MHz, $CDCl_3$) 7.43-7.33 (m, 10H), 7.02 (s, 4H), 6.48 (m, 2H), 6.40 (d, J = 7.3 Hz, 4H), 4.83 (s, 4H), 4.34 (d, J = 13.3 Hz, 4H), 3.76-3.72 (m, 4H), 3.05 (d, J = 13.4 Hz, 4H), 1.70-1.60 (m, 4H), 0.65 (t, J = 7.5 Hz, 6H). $^{13}C\{^1H\}$ NMR (100 MHz, $CDCl_3$) 156.5, 154.7, 138.1, 137.4, 133.5, 131.0, 129.3, 128.2, 128.1, 128.0, 122.8, 114.5, 77.1, 76.7, 30.9, 22.8, 9.6. HRMS (TOF MS ES+) m/z: $[M+Na]^+$ calcd for $C_{48}H_{46}Br_2O_4Na$ 869.1640, found 869.1654.

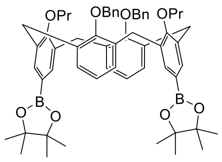

8

Compound 8: Compound **8** synthesis is based on a reported literature method.[10] A mixture of compound **III** (1.0 g, 1.2 mmol), Pd(dppf)Cl_2 (52 mg, 0.07 mmol), K_2CO_3 (0.70 g, 7.1 mmol), dry DMF (20 mL), and Bis(pinacolato)diboron (0.90 g, 3.5 mmol) was stirred under nitrogen atmosphere at 85 °C for 48 hours. After cooling the reaction to room temperature, the solvent was evaporated under vacuum, and the residue was dissolved in DCM and washed with water and brine. The organic phase was dried over $MgSO_4$, filtered, and concentrated to dryness. The crude product was purified using Combi*Flash*® (CH_2Cl_2-hexane, 1:1) to give a white solid **8**. Yield: 69% (0.77 g). ^{1}H NMR (100 MHz, $CDCl_3$) 7.59 (s, 4H), 7.48-7.31 (m, 10H), 6.26-6.23 (m, 2H), 6.16 (d, J = 7.5 Hz, 4H), 4.71 (s, 4H), 4.42 (d, J = 13.3 Hz, 4H), 3.88-3.84 (m, 4H), 3.16 (d, J = 13.4 Hz, 4H), 1.66-1.58 (m, 4H), 1.39 (s, 24H), 0.52 (t, J = 7.4 Hz, 6H). ^{13}C{^{1}H} NMR (100 MHz, $CDCl_3$) 161.2, 154.5, 137.7, 136.5, 135.7, 133.2, 128.9, 128.3, 127.9, 127.7, 122.5, 121.9, 83.6, 77.5, 76.5, 31.0, 25.1, 22.9, 9.4. HRMS (TOF MS ES+) m/z: $[M+Na]^+$ calcd for $C_{60}H_{70}B_2O_8Na$ 963.5175, found 963.5154.

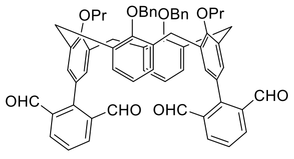

9

Compound 9: Compound **9** synthesis is based on a reported literature method.[11] A mixture of compound **8** (1.6 g, 1.7 mmol), 2-Bromoisophthalaldehyde (**4**) (1.5 g, 6.9 mmol), $NaHCO_3$ (0.87 g, 10.4 mmol), DMSO (48 mL) and water (8 mL) was degassed under N_2 for 20 minutes. Pd(dppf)Cl_2 (64 mg, 0.087 mmol) was added, and the reaction was stirred under a nitrogen atmosphere at 85 °C for 48 hours. After cooling to room temperature and diluting with water, the mixture was extracted with DCM (X3). The organic phases were combined, washed with brine solution, dried over Na_2SO_4, filtered and concentrated under vacuum. The crude product was purified using Combi*Flash*® (CH_2Cl_2-hexane, 1:1 to 100% DCM) to give a bright yellow solid **9**. Yield: 74% (1.2 g). ^{1}H NMR (400 MHz, $CDCl_3$) 9.78 (s, 2H), 8.25 (br s, 2H), 8.09 (dd, J = 7.5, 1.5 Hz, 2H), 7.46-7.33 (m, 14H), 6.86 (d, J = 7.4 Hz, 4H), 6.78-6.74 (m, 2H), 6.61 (s, 4H), 5.29 (s, 4H), 4.38 (d, J = 13.2 Hz, 4H), 3.81-3.77 (m, 4H), 3.14 (d, J = 13.3 Hz, 4H), 1.87-1.78 (m, 4H), 0.94 (t, J = 7.4 Hz, 6H). ^{13}C{^{1}H} NMR (100 MHz, $CDCl_3$) 191.6, 189.6, 157.0, 154.8, 147.8, 137.5, 135.7, 134.7, 134.3, 133.7, 132.6, 132.5, 132.0, 130.3, 128.9, 128.2, 128.0, 127.0, 124.8, 123.4, 77.5, 76.4, 31.3, 23.1, 10.3 HRMS (TOF MS ES+) m/z: $[M+Na]^+$ calcd for $C_{64}H_{56}O_8Na$ 975.3873, found 975.3873.

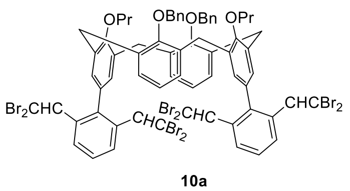

10a

Compound 10a: Triphenylphosphine (3.1 g, 11.7 mmol) was dissolved in DCM (40 mL) and cooled to 0 °C. Carbon tetrabromide (1.9 g, 5.8 mmol) was added, and after the solution became yellow (1 minute of stirring), compound **9** (700 mg, 0.73 mmol) in DCM (20 mL) at 0 °C was added dropwise. The mixture was left stirring at room temperature for 40 minutes (monitoring with TLC, EtOAc-hexane,

1:9). Then water was added, and the product was extracted with DCM. The combined organic phases were washed with water and brine, dried over Na_2SO_4, and filtered, and the solvent was reduced under vacuum. The crude product was purified using Combi*Flash*® (CH_2Cl_2-hexane, 1:2) to give a white solid **10a**. Yield: 83% (952 mg). ^{1}H NMR (400 MHz, $CDCl_3$) 7.75 (d, *J* = 7.8 Hz, 4H), 7.56-7.54 (m, 4H), 7.45-7.34 (m, 10H), 7.10 (br s, 2H), 6.91 (s, 4H), 6.46-6.42 (m, 2H), 6.25 (d, *J* = 7.6 Hz, 4H), 4.75 (s, 4H), 4.56 (d, *J* = 13.3 Hz, 4H), 3.99-3.94 (m, 4H), 3.18 (d, *J* = 13.4 Hz, 4H), 1.78-1.69 (m, 4H), 0.64 (t, *J* = 7.5 Hz, 6H). ^{13}C{^{1}H} NMR (100 MHz, CD_2Cl_2) 157.9, 154.5, 140.9, 137.7, 137.2, 137.0, 136.9, 135.4, 135.3, 132.8, 130.8, 129.9, 128.6, 128.1, 127.7, 127.5, 126.6, 123.0, 90.7, 90.2, 77.3, 76.4, 30.9, 22.9, 9.2. HRMS (TOF MS ES+) m/z: $[M+Na]^+$ calcd for $C_{68}H_{56}Br_8O_4Na$ 1598.7461, found 1598.7493.

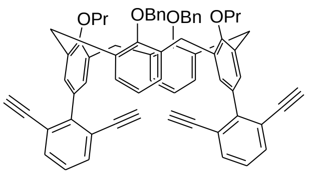

10

Compound 10: Compound **10a** (1.0 g, 0.63 mmol) was dissolved in dry THF (30 mL) and cooled to -78 °C under a nitrogen atmosphere. LDA (2M in THF, 5.1 mL, 10.15 mmol) was added dropwise, and the solution was stirred at -78 °C for 1 hour. After stirring for another hour at room temperature, the reaction was quenched with HCl 1M solution. The mixture was extracted with DCM (X3), and the combined organic phases were washed with water and brine, dried over $MgSO_4$, filtered, and concentrated under reduced pressure. The crude product was purified using Combi*Flash*® (CH_2Cl_2-hexane, 3:7) to give a white solid **10**. Yield: 69% (410 mg). ^{1}H NMR (400 MHz, $CDCl_3$) 7.63 (d, *J* = 7.7 Hz, 4H), 7.54-7.52 (m, 4H), 7.41-7.35 (m, 6H), 7.29 (t, *J* = 7.8 Hz, 2H), 7.20 (s, 4H), 6.33 (d, *J* = 7.5 Hz, 4H), 6.24-6.20 (m, 2H), 4.76 (s, 4H), 4.54 (d, *J* = 13.2 Hz, 4H), 3.96-3.92 (m, 4H), 3.19 (d, *J* = 13.4 Hz, 4H), 3.02 (s, 4H), 1.78-1.74 (m, 4H), 0.63 (t, *J* = 7.5 Hz, 6H). ^{13}C{^{1}H} NMR (100 MHz, $CDCl_3$) 157.6, 154.5, 147.8, 137.8, 136.0, 133.4, 133.2, 132.0, 130.1, 128.6, 128.1, 127.9, 127.7, 126.6, 122.4, 121.9, 82.7, 80.5, 77.2, 76.3, 31.0, 22.9, 9.4. HRMS (TOF MS ES+) m/z: $[M+Na]^+$ calcd for $C_{68}H_{56}O_4Na$ 959.4076, found 959.4084.

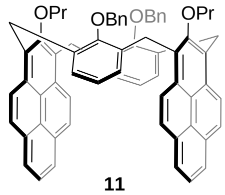

11

Compound 11: Compound **10** (206 mg, 0.22 mmol) was dissolved in dry toluene (10 mL) under a nitrogen atmosphere. Platinum(II) chloride (11.7 mg, 0.04 mmol) was added, and the solution was stirred at 80 °C for 24 hours. After completion of the reaction was determined by TLC (CH2Cl2-hexane, 1:2), The solution was concentrated under reduced pressure, and the crude product was purified using Combi*Flash*® (CH_2Cl_2-hexane, 3:7) to give white solid **11**. Yield: 12% (25 mg). ^{1}H NMR (400 MHz, $CDCl_3$) 8.48 (d, *J* = 9.4 Hz, 4H), 8.14 (d, *J* = 7.6 Hz, 4H), 8.10 (d, *J* = 9.3 Hz, 4H), 7.95 (t, *J* = 7.6 Hz, 2H), 7.69-7.69-7.68 (m, 4H), 7.49-7.40 (m, 6H), 5.79 (d, *J* = 7.1 Hz, 4H), 5.74-5.71 (m, 2H), 4.99 (s, 4H), 4.93 (d, *J* = 13.9 Hz, 4H), 4.40 (d, *J* = 13.9 Hz, 4H), 4.04-4.00 (m, 4H), 1.99-1.89 (m, 4H), 0.61 (t, *J* = 7.5 Hz, 6H). ^{13}C{^{1}H} NMR (100 MHz, $CDCl_3$) 156.2, 154.6, 137.8, 133.3, 132.1, 130.7, 129.2, 129.1, 128.4, 128.1, 127.2, 127.1, 125.4, 125.1, 124.5, 123.9, 122.5, 122.0, 77.6, 77.5, 24.8, 22.7, 9.5. HRMS (TOF MS ES+) m/z: $[M+Na]^+$ calcd for $C_{68}H_{56}O_4Na$ 959.4076, found 959.4072.

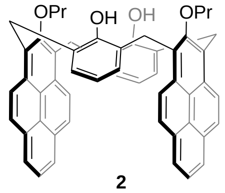

2

Compound 2: Compound **11** (24.9 mg, 0.026 mmol) was dissolved in DCM (2 mL), and then MeCN (1 mL) and HBr 48% (1 mL) were added. The solution was stirred at 60 °C for 24 hours. After completion of the reaction was determined by TLC (CH_2Cl_2-hexane, 2:3), The reaction was neutralized with $NaHCO_3$ conc. solution and extraction with DCM was repeated several times. The organic phase was washed with water and brine, filtered, and concentrated under vacuum. The crude product was purified using Combi*Flash*® (CH_2Cl_2-hexane, 3:7) to give white solid **2**. Yield: 62% (12.3 mg). ^{1}H NMR (400 MHz, $CDCl_3$) 8.47 (s, 2H), 8.13 (d, *J* = 9.3 Hz, 4H), 7.55 (d, *J* = 7.6 Hz, 4H), 7.36 (d, *J* = 9.2 Hz, 4H), 7.04-7.02 (br m, 4H), 6.91 (t, *J* = 7.6 Hz, 2H), 6.78 (br s, 2H), 4.88 (d, *J* = 13.9 Hz, 4H), 4.33 (d, *J* = 13.9 Hz, 4H), 4.11-4.08 (m, 4H), 2.23-2.15 (m, 4H), 1.45 (t, *J* = 7.4 Hz, 6H). ^{13}C{^{1}H} (100 MHz, $CDCl_3$) 155.6, 151.3, 130.1, 129.9, 129.2, 126.6, 126.6, 125.8, 124.4, 124.1, 123.6, 123.3, 122.9, 117.9, 78.4, 29.5, 23.8, 11.2. HRMS (TOF MS ES+) m/z: $[M+Na]^+$ calcd for $C_{54}H_{44}O_4Na$ 779.3137, found 779.3151.

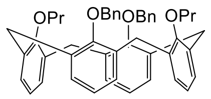

13

Compound 13: Compound **IV** (1.9 g, 3.1 mmol) was dissolved in DMF (20 mL) and stirred at 0 °C. Sodium hydride (0.62 g, 15.5 mmol) was added in batches, followed by the addition of n-propyl iodide (1.5 mL, 15.5 mmol). The reaction was left stirring for 12 hours. After all the starting material was consumed (TLC, CH_2Cl_2-hexane, 3:7), the mixture was cooled to 0 °C and quenched with HCl 1M solution (10 mL). The precipitate was stirred for 15 minutes at 0 °C and collected by vacuum filtration. The filter cake was washed with water and MeOH and dried under a vacuum to obtain a white solid **13**. Yield: 84% (1.8 g). ^{1}H NMR (400 MHz, $CDCl_3$) 7.52-7.50 (m, 4H), 7.41-7.33 (m, 6H), 6.92 (d, *J* = 7.4 Hz, 4H), 6.81-6.77 (m, 2H), 6.46-6.38 (m, 6H), 4.88 (s, 4H), 4.46 (d, *J* = 13.3 Hz, 4H), 3.84-3.80 (m, 4H), 3.15 (d, *J* = 13.4 Hz, 4H), 1.78-1.68 (m, 4H), 0.69 (t, *J* = 7.5 Hz, 6H). ^{13}C{^{1}H} NMR (100 MHz, $CDCl_3$) 157.4, 154.9, 137.9, 136.2, 134.3, 129.2, 128.6, 128.2, 127.9, 127.8, 122.4, 121.9, 77.0, 76.6, 31.2, 23.0, 9.7. HRMS (TOF MS ES+) m/z: $[M+Na]^+$ calcd for $C_{48}H_{48}O_4Na$ 711.3450, found 711.3458.

3. NMR and Mass spectra

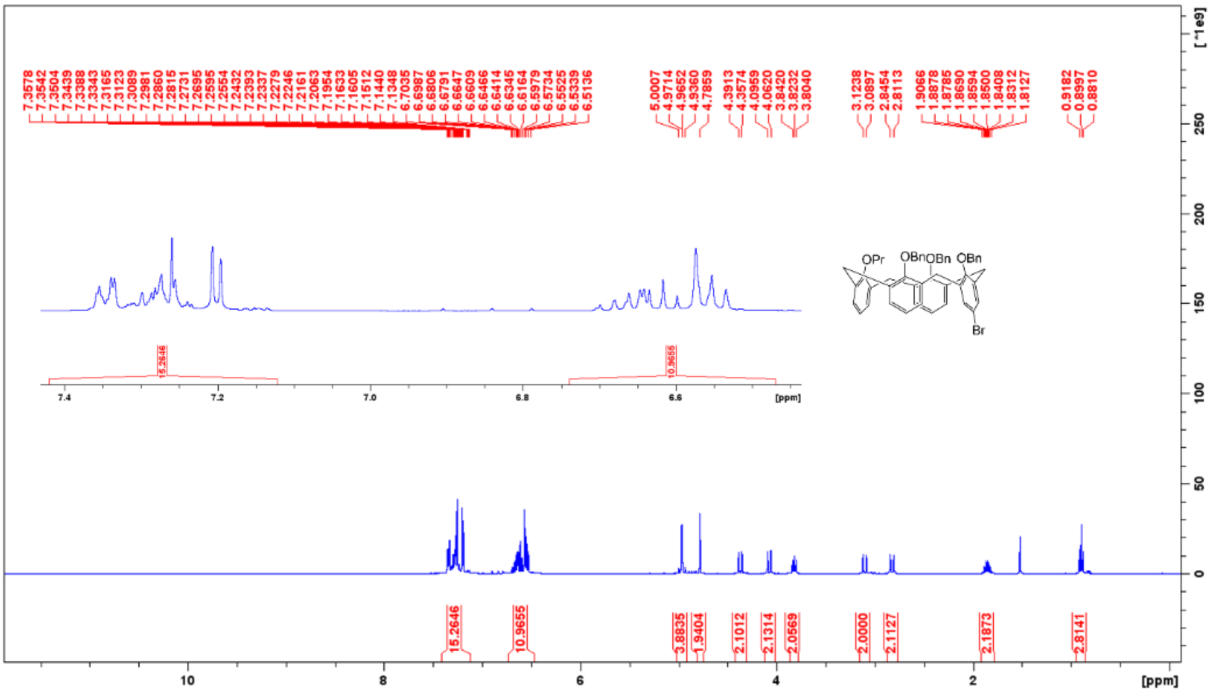

Fig. S1 - ^{1}H NMR spectrum of ***I*** (400 MHz, $CDCl_3$)

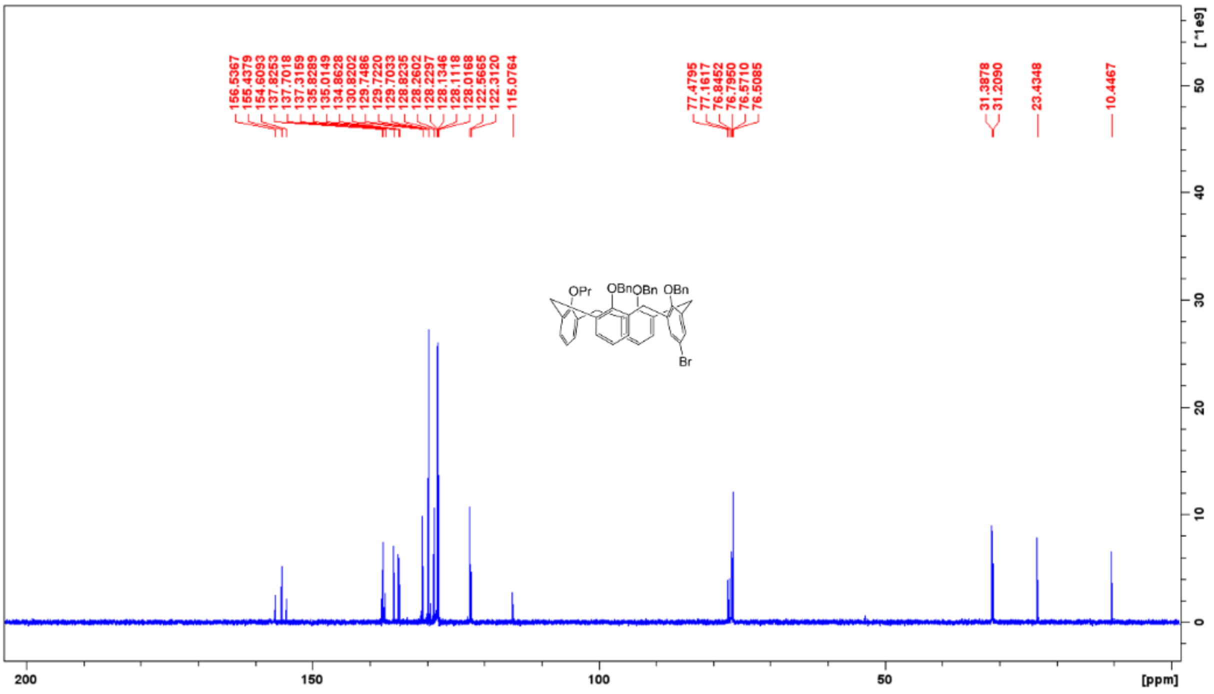

Fig. S2 - ^{13}C{^{1}H} NMR spectrum of ***I*** (100 MHz, $CDCl_3$)

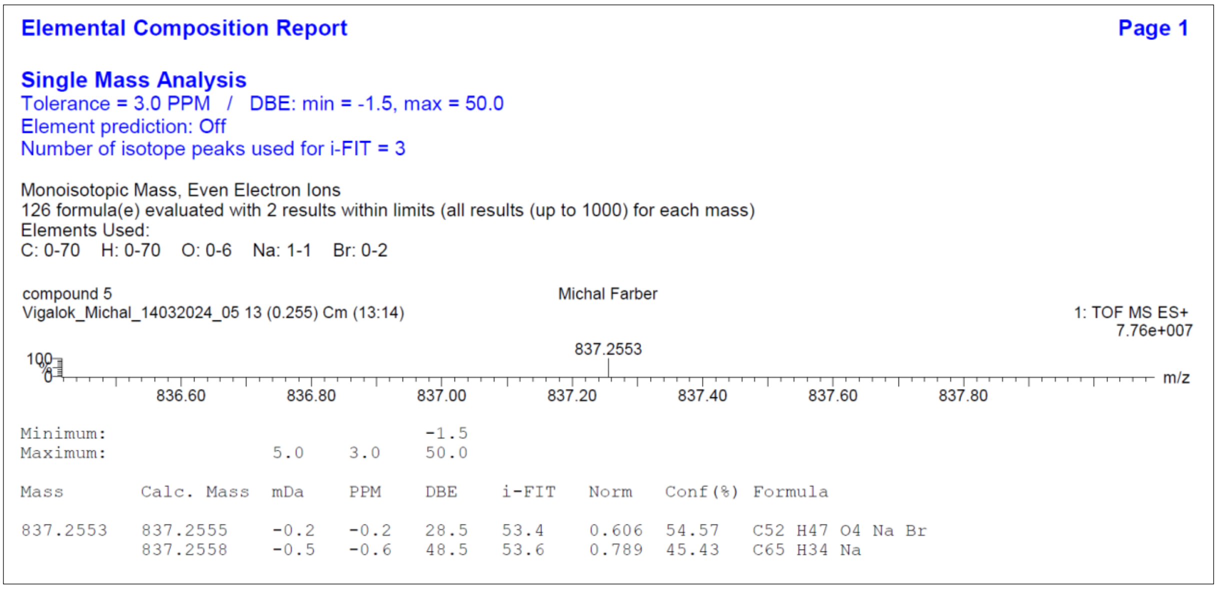

Elemental Composition Report | Page 1

Single Mass Analysis
Tolerance = 3.0 PPM / DBE: min = -1.5, max = 50.0
Element prediction: Off
Number of isotope peaks used for i-FIT = 3

Monoisotopic Mass, Even Electron Ions
126 formula(e) evaluated with 2 results within limits (all results (up to 1000) for each mass)
Elements Used:
C: 0-70 H: 0-70 O: 0-6 Na: 1-1 Br: 0-2

compound 5 — Michal Farber
Vigalok_Michal_14032024_05 13 (0.255) Cm (13:14) — 1: TOF MS ES+ 7.76e+007

Minimum:				-1.5				
Maximum:		5.0	3.0	50.0				
Mass	Calc. Mass	mDa	PPM	DBE	i-FIT	Norm	Conf(%)	Formula
837.2553	837.2555	-0.2	-0.2	28.5	53.4	0.606	54.57	C52 H47 O4 Na Br
	837.2558	-0.5	-0.6	48.5	53.6	0.789	45.43	C65 H34 Na

Fig. S3 - Mass spectrum of ***1***

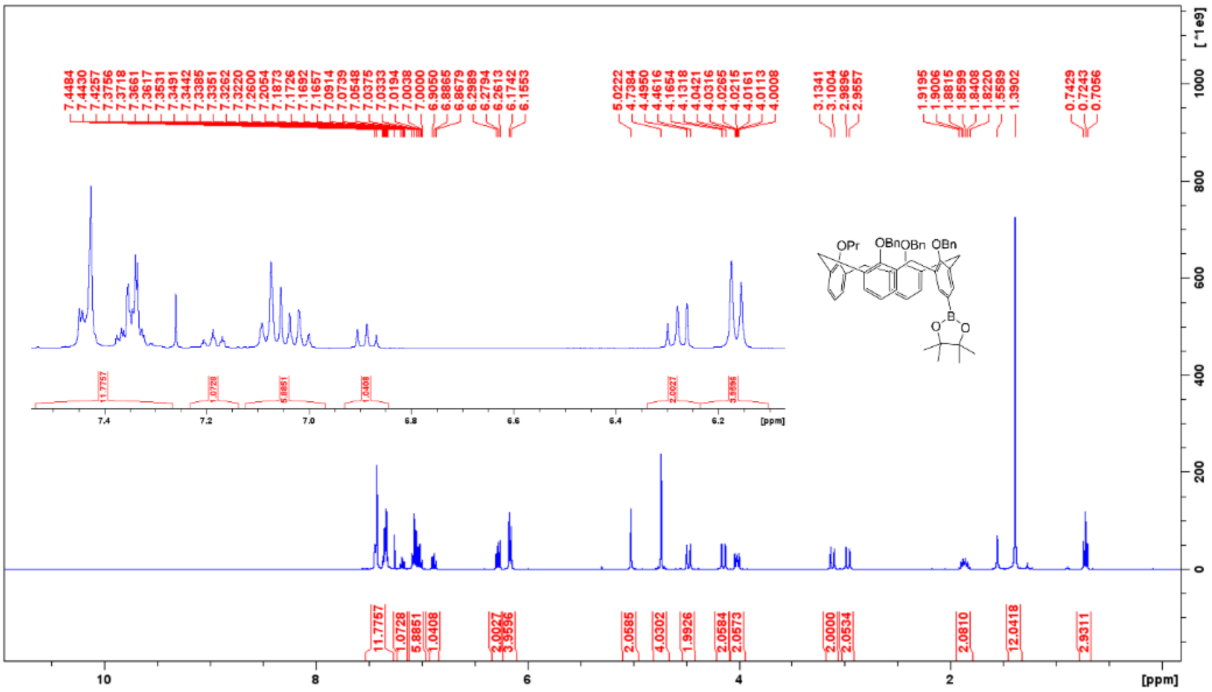

Fig. S4 - ^{1}H NMR spectrum of ***3*** (400 MHz, $CDCl_3$)

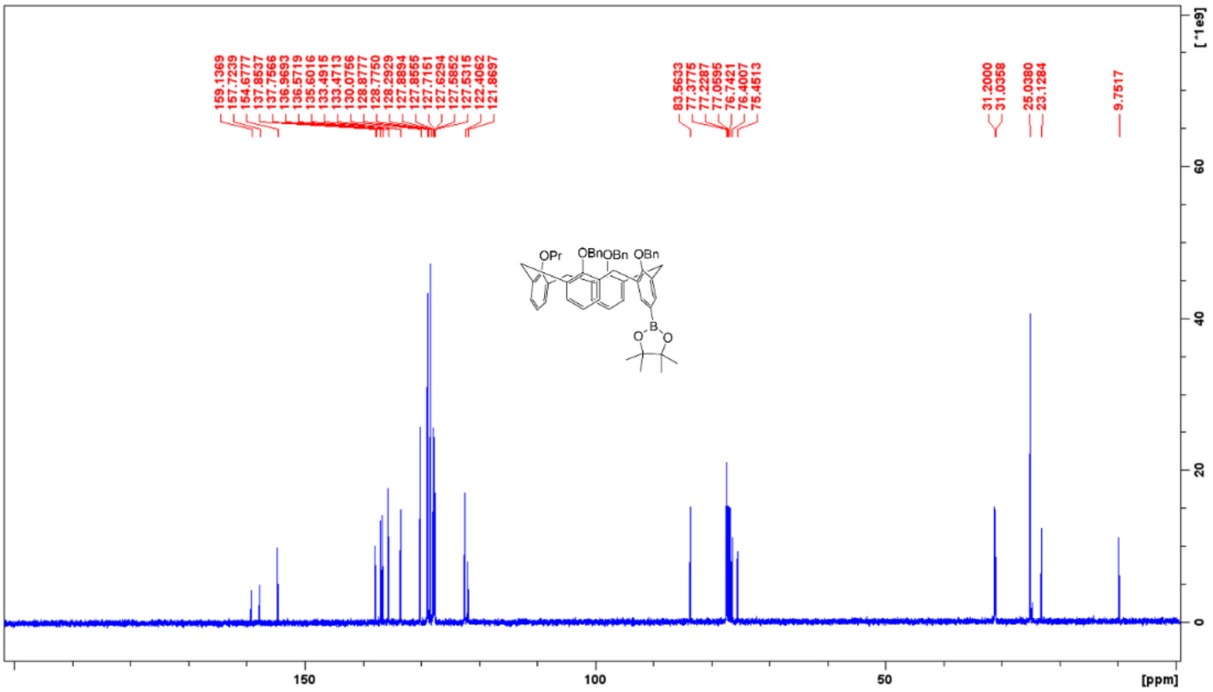

*Fig. S5 - $^{13}C\{^1H\}$ NMR spectrum of **3*** (100 MHz, $CDCl_3$)

Elemental Composition Report

Page 1

Single Mass Analysis

Tolerance = 3.0 PPM / DBE: min = -1.5, max = 50.0
Element prediction: Off
Number of isotope peaks used for i-FIT = 3

Monoisotopic Mass, Even Electron Ions
124 formula(e) evaluated with 1 results within limits (all results (up to 1000) for each mass)
Elements Used:
C: 0-70 H: 0-70 O: 0-6 Na: 1-1 B: 0-2

compound 6 Michal Farber
Vigalok_Michal_14032024_06 8 (0.161) Cm (8:9)
1: TOF MS ES+
4.94e+008

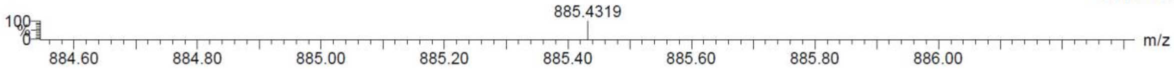

Minimum:				-1.5				
Maximum:		5.0	3.0	50.0				
Mass	Calc. Mass	mDa	PPM	DBE	i-FIT	Norm	Conf(%)	Formula
885.4319	885.4302	1.7	1.9	29.5	48.0	n/a	n/a	C58 H59 O6 Na B

*Fig. S6 - Mass spectrum of **3***

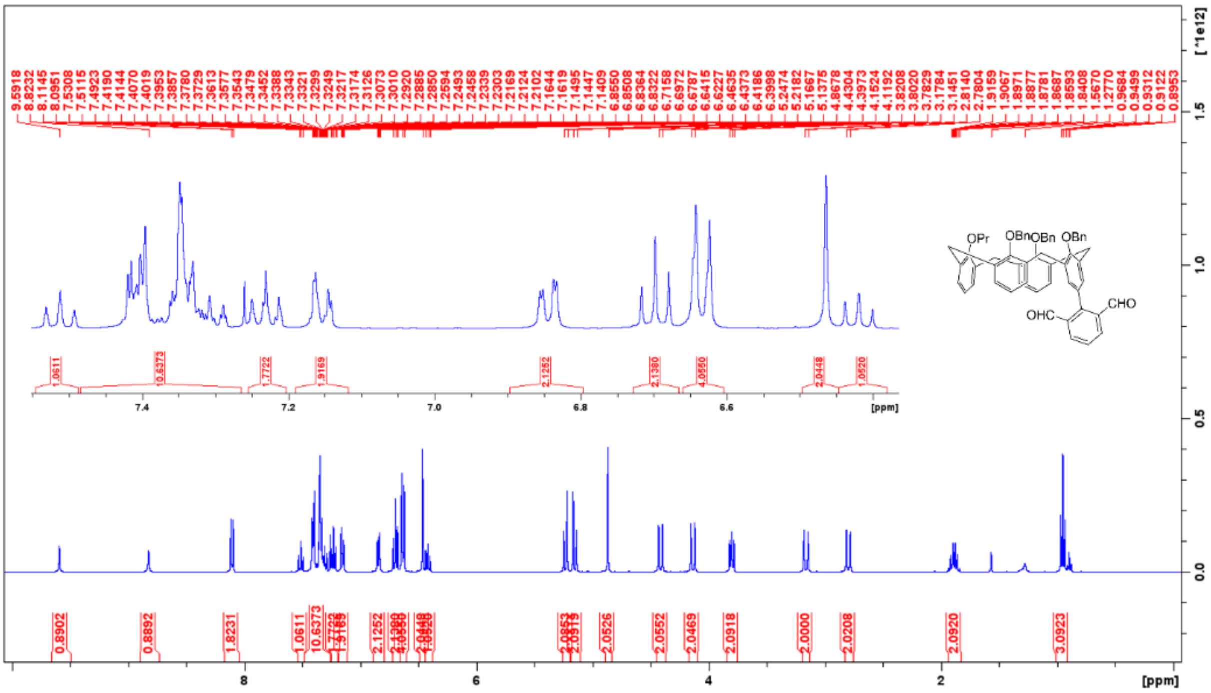

*Fig. S7 - ^{1}H NMR spectrum of **5*** (400 MHz, $CDCl_3$)

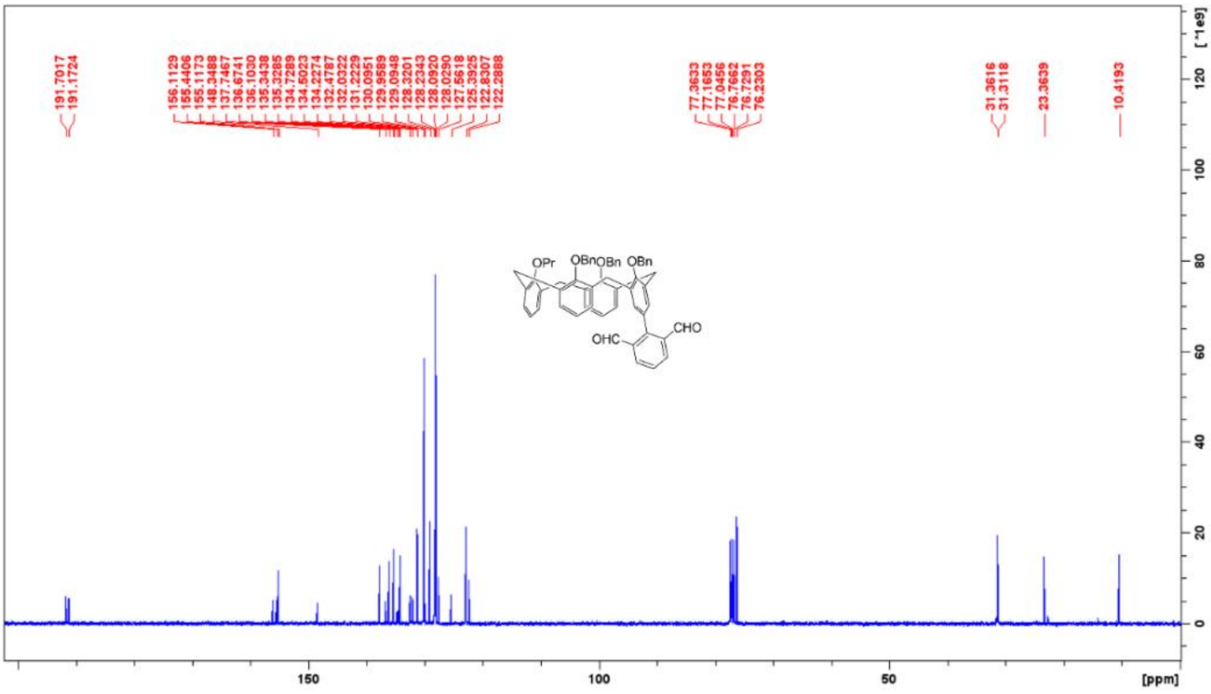

*Fig. S8 - ^{13}C{^{1}H} NMR spectrum of **5*** (100 MHz, $CDCl_3$)

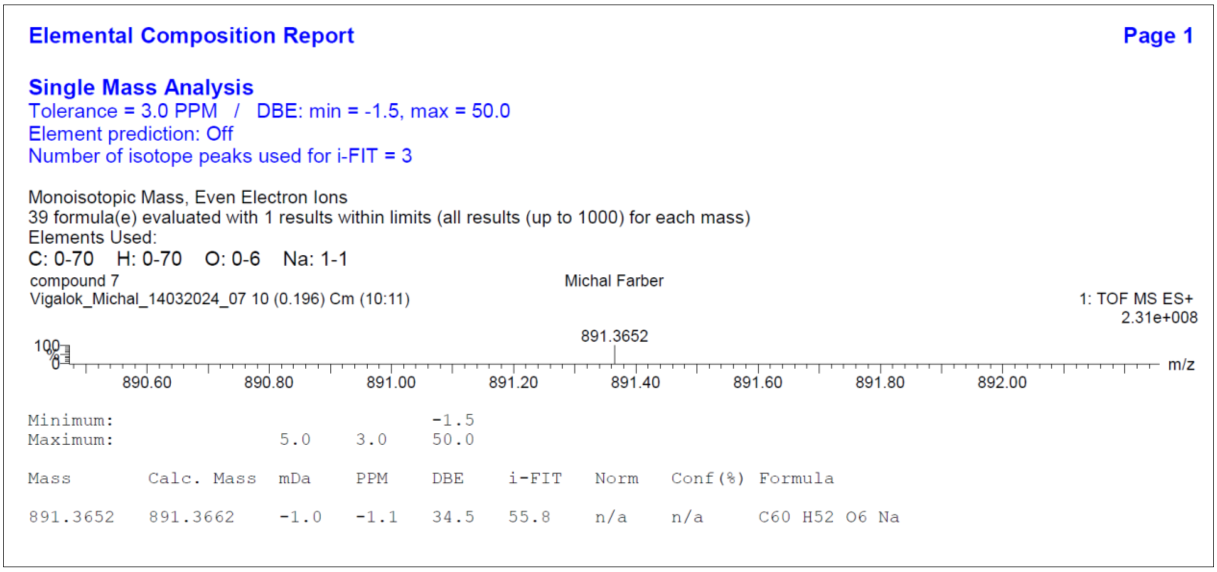

Elemental Composition Report Page 1

Single Mass Analysis
Tolerance = 3.0 PPM / DBE: min = -1.5, max = 50.0
Element prediction: Off
Number of isotope peaks used for i-FIT = 3

Monoisotopic Mass, Even Electron Ions
39 formula(e) evaluated with 1 results within limits (all results (up to 1000) for each mass)
Elements Used:
C: 0-70 H: 0-70 O: 0-6 Na: 1-1
compound 7 Michal Farber
Vigalok_Michal_14032024_07 10 (0.196) Cm (10:11) 1: TOF MS ES+ 2.31e+008

	Calc. Mass	mDa	PPM	DBE	i-FIT	Norm	Conf(%)	Formula
Minimum:				-1.5				
Maximum:		5.0	3.0	50.0				
Mass	Calc. Mass	mDa	PPM	DBE	i-FIT	Norm	Conf(%)	Formula
891.3652	891.3662	-1.0	-1.1	34.5	55.8	n/a	n/a	C60 H52 O6 Na

*Fig. S9 - Mass spectrum of **5***

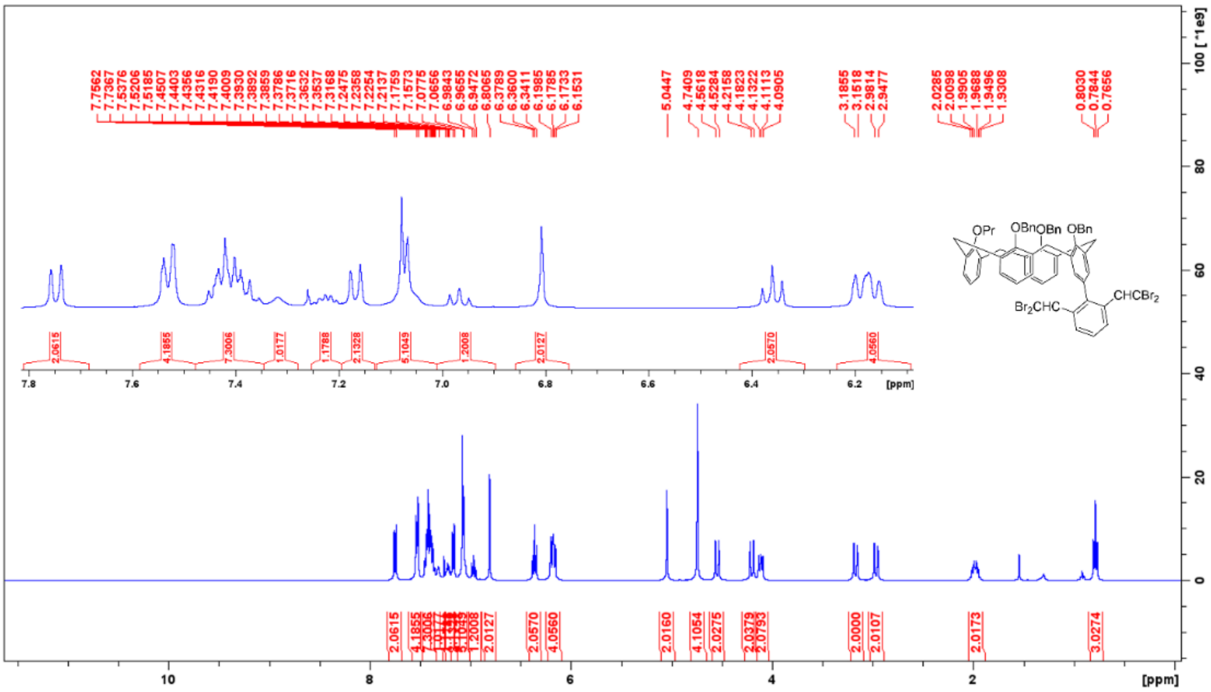

*Fig. S10 – ^{1}H NMR spectrum of **6a*** (400 MHz, $CDCl_3$)

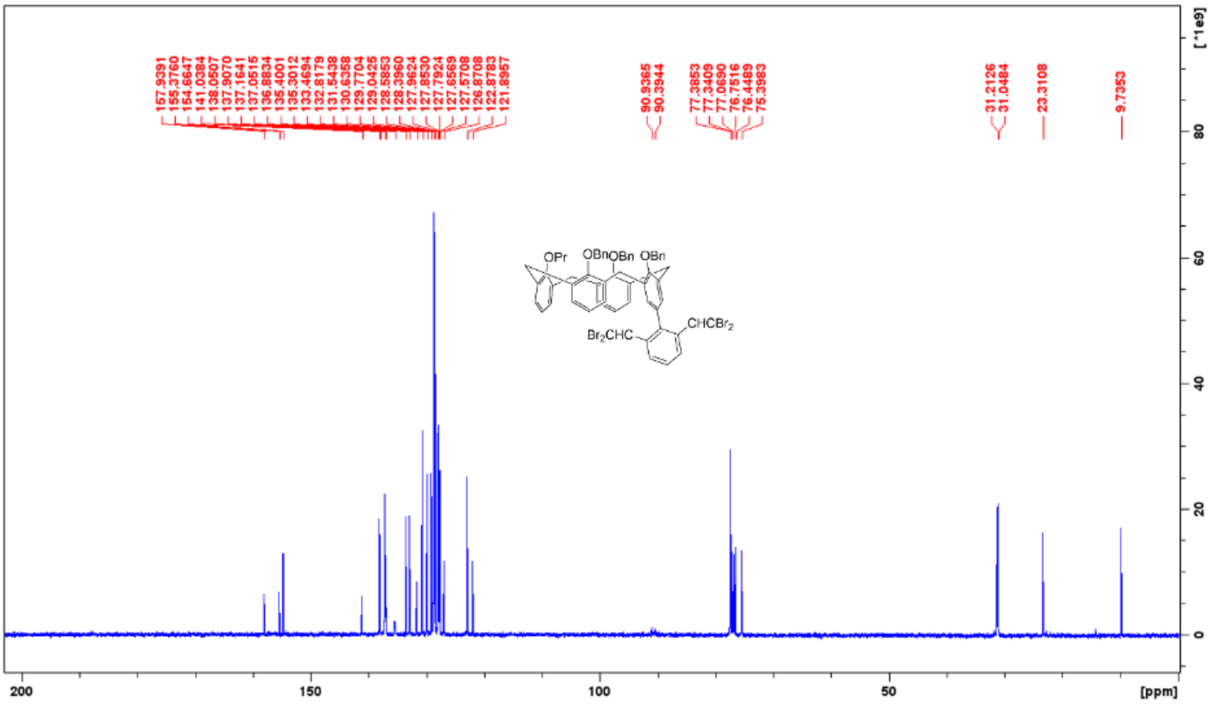

*Fig. S11 - $^{13}C\{^1H\}$ NMR spectrum of **6a*** (100 MHz, $CDCl_3$)

Elemental Composition Report

Page 1

Single Mass Analysis

Tolerance = 3.0 PPM / DBE: min = -1.5, max = 50.0
Element prediction: Off
Number of isotope peaks used for i-FIT = 3

Monoisotopic Mass, Even Electron Ions
272 formula(e) evaluated with 1 results within limits (all results (up to 1000) for each mass)
Elements Used:
C: 0-70 H: 0-70 O: 0-4 Na: 1-1 79Br: 2-4 81Br: 2-4

compound 8 — Michal Farber
Vigalok_Michal_14032024_08 12 (0.238) Cm (12:13) — 1: TOF MS ES+ 1.24e+006

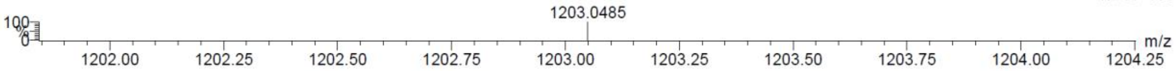

Minimum:				-1.5				
Maximum:		5.0	3.0	50.0				
Mass	Calc. Mass	mDa	PPM	DBE	i-FIT	Norm	Conf(%)	Formula
1203.0485	1203.0456	2.9	2.4	34.5	44.9	n/a	n/a	C62 H52 O4 Na 79Br2 81Br2

*Fig. S12 - Mass spectrum of **6a***

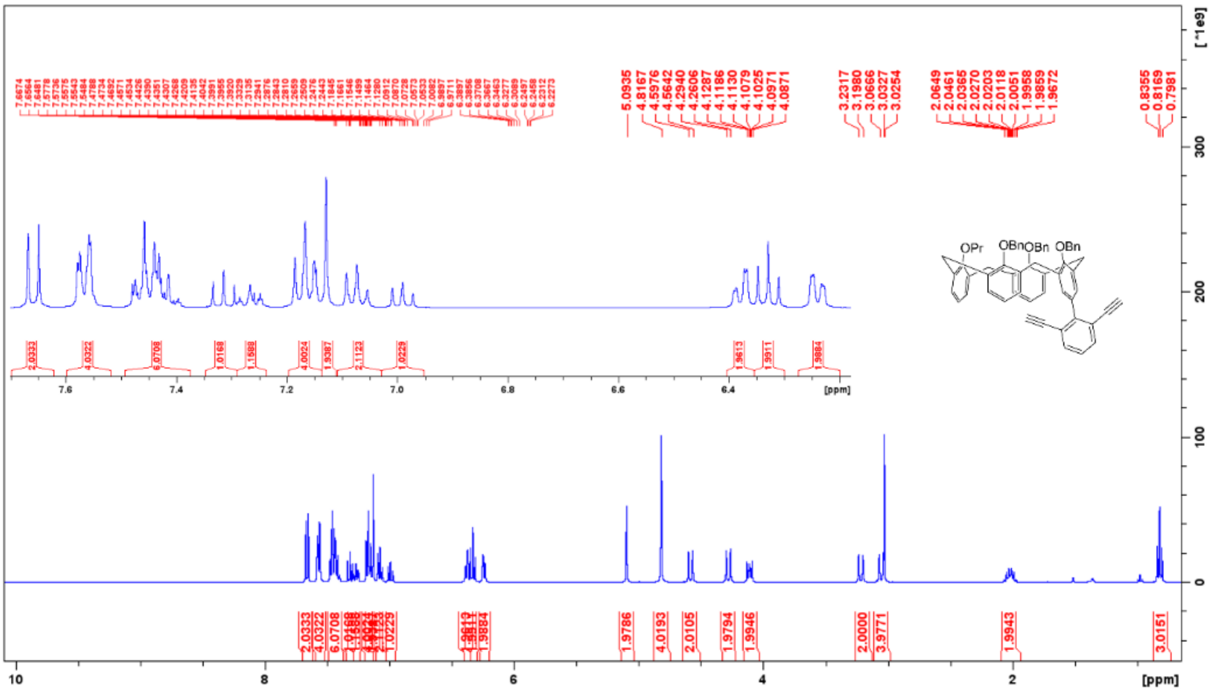

*Fig. S13 - ^{1}H NMR spectrum of **6*** (400 MHz, $CDCl_3$)

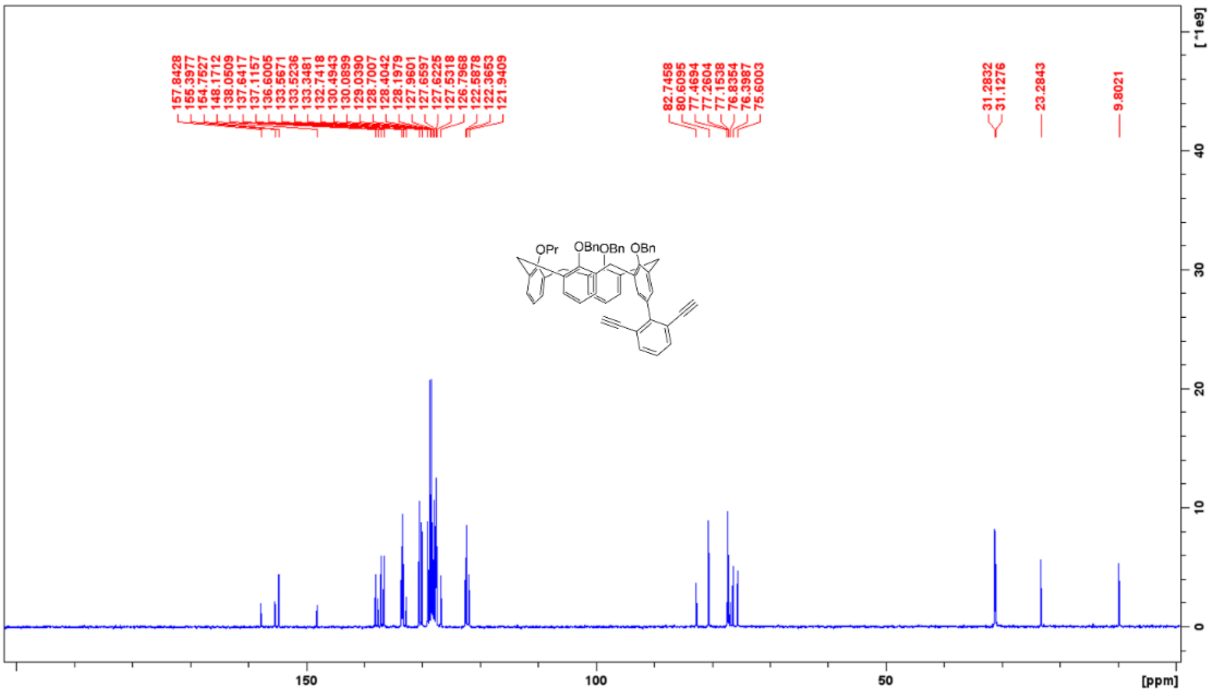

*Fig. S14 - $^{13}C\{^1H\}$ NMR spectrum of **6*** (100 MHz, $CDCl_3$)

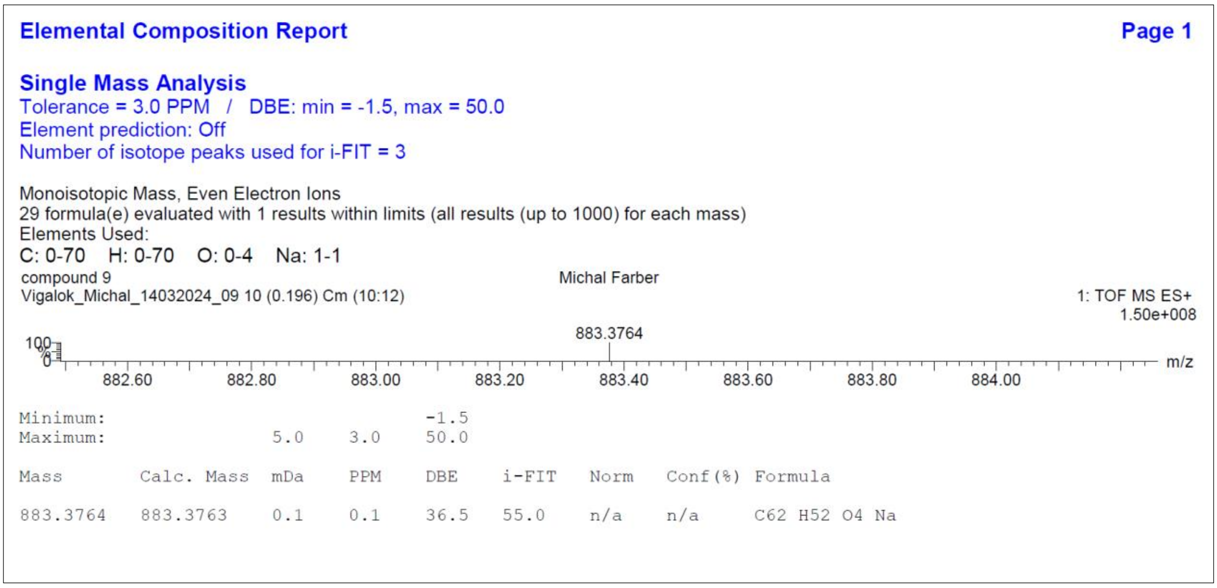

Elemental Composition Report

Page 1

Single Mass Analysis

Tolerance = 3.0 PPM / DBE: min = -1.5, max = 50.0
Element prediction: Off
Number of isotope peaks used for i-FIT = 3

Monoisotopic Mass, Even Electron Ions
29 formula(e) evaluated with 1 results within limits (all results (up to 1000) for each mass)
Elements Used:
C: 0-70 H: 0-70 O: 0-4 Na: 1-1
compound 9 Michal Farber
Vigalok_Michal_14032024_09 10 (0.196) Cm (10:12) 1: TOF MS ES+ 1.50e+008

Minimum:				-1.5				
Maximum:		5.0	3.0	50.0				
Mass	Calc. Mass	mDa	PPM	DBE	i-FIT	Norm	Conf(%)	Formula
883.3764	883.3763	0.1	0.1	36.5	55.0	n/a	n/a	C62 H52 O4 Na

Fig. S15 - Mass spectrum of ***6***

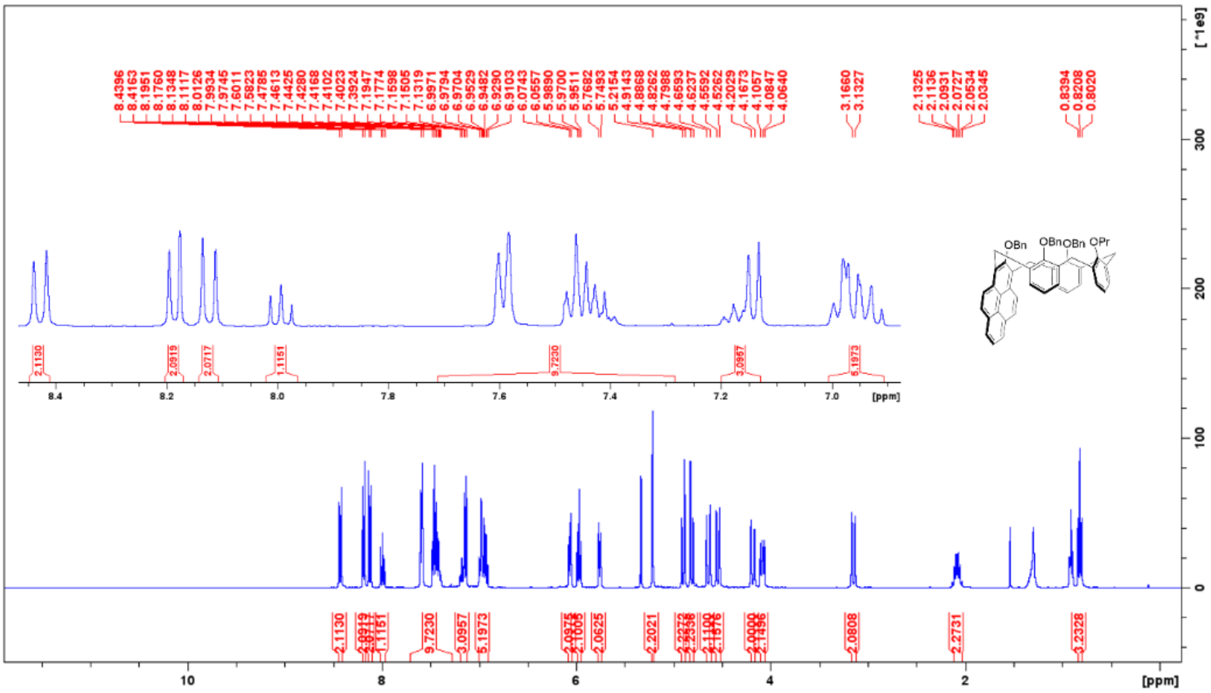

Fig. S16 - ^{1}*H NMR spectrum of* ***7*** (400 MHz, CD_2Cl_2)

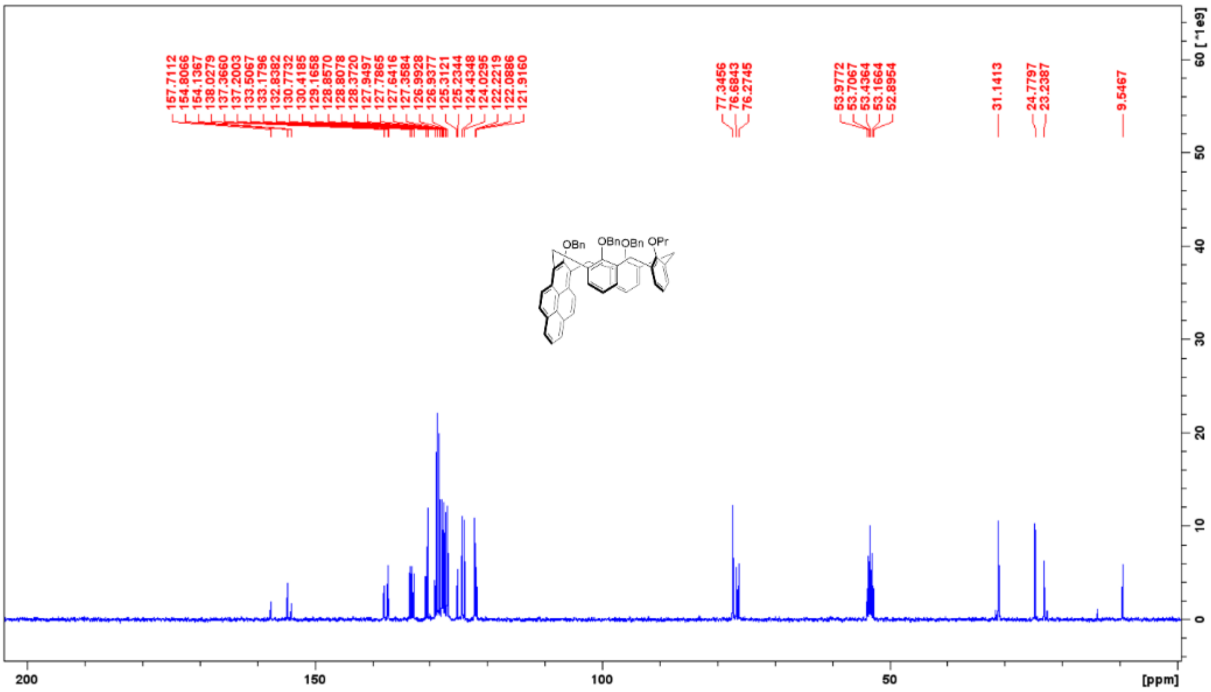

*Fig. S17 - $^{13}C\{^{1}H\}$ NMR spectrum of **7*** (100 MHz, CD_2Cl_2)

Elemental Composition Report **Page 1**

Single Mass Analysis
Tolerance = 3.0 PPM / DBE: min = -1.5, max = 50.0
Element prediction: Off
Number of isotope peaks used for i-FIT = 3

Monoisotopic Mass, Even Electron Ions
29 formula(e) evaluated with 1 results within limits (all results (up to 1000) for each mass)
Elements Used:
C: 0-70 H: 0-70 O: 0-4 Na: 1-1
compound 10 Michal Farber
Vigalok_Michal_14032024_10 10 (0.196) Cm (10) 1: TOF MS ES+ 5.61e+007

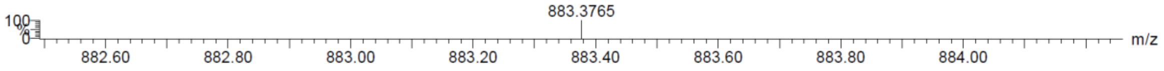

Minimum:				-1.5				
Maximum:		5.0	3.0	50.0				
Mass	Calc. Mass	mDa	PPM	DBE	i-FIT	Norm	Conf(%)	Formula
883.3765	883.3763	0.2	0.2	36.5	53.0	n/a	n/a	C62 H52 O4 Na

*Fig. S18 - Mass spectrum of **7***

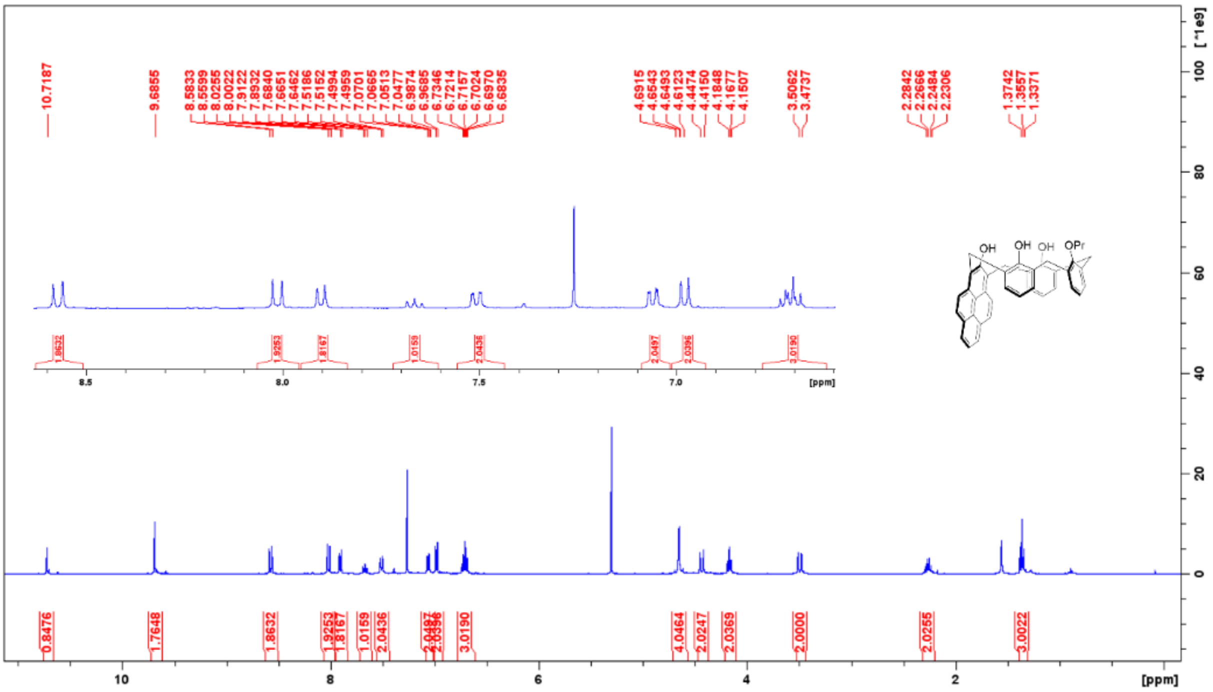

*Fig. S19 – ^{1}H NMR spectrum of **1*** (400 MHz, $CDCl_3$)

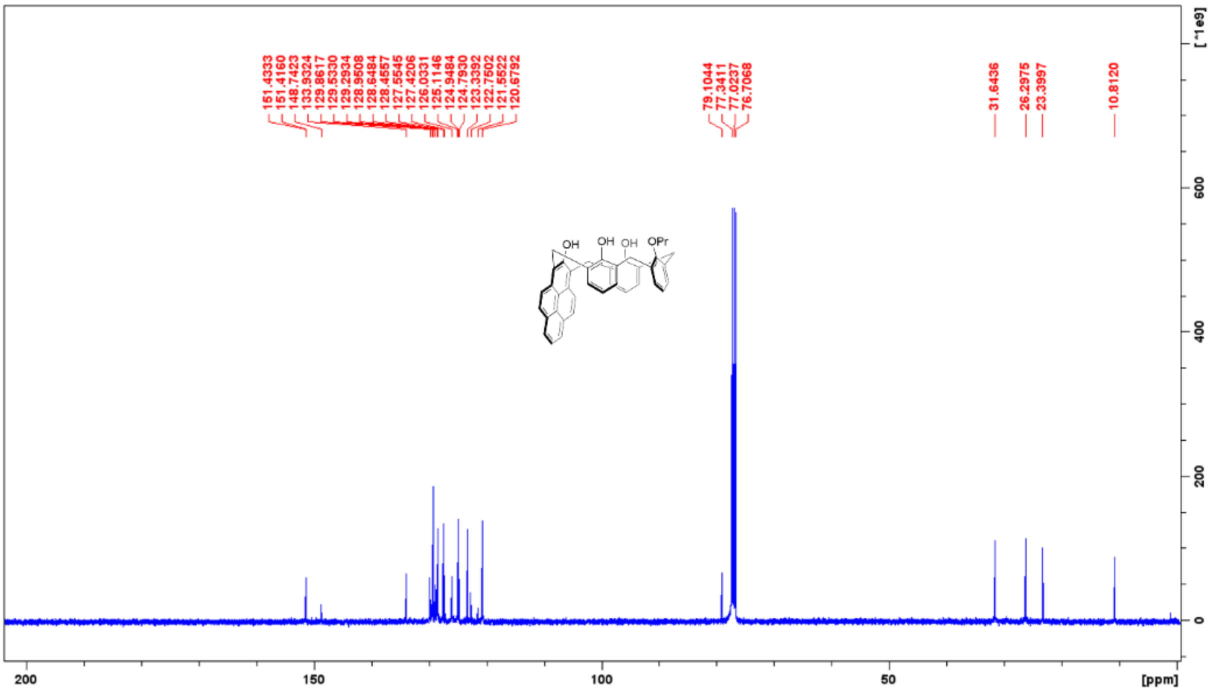

*Fig. S20 - ^{13}C{^{1}H} NMR spectrum of **1*** (100 MHz, $CDCl_3$)

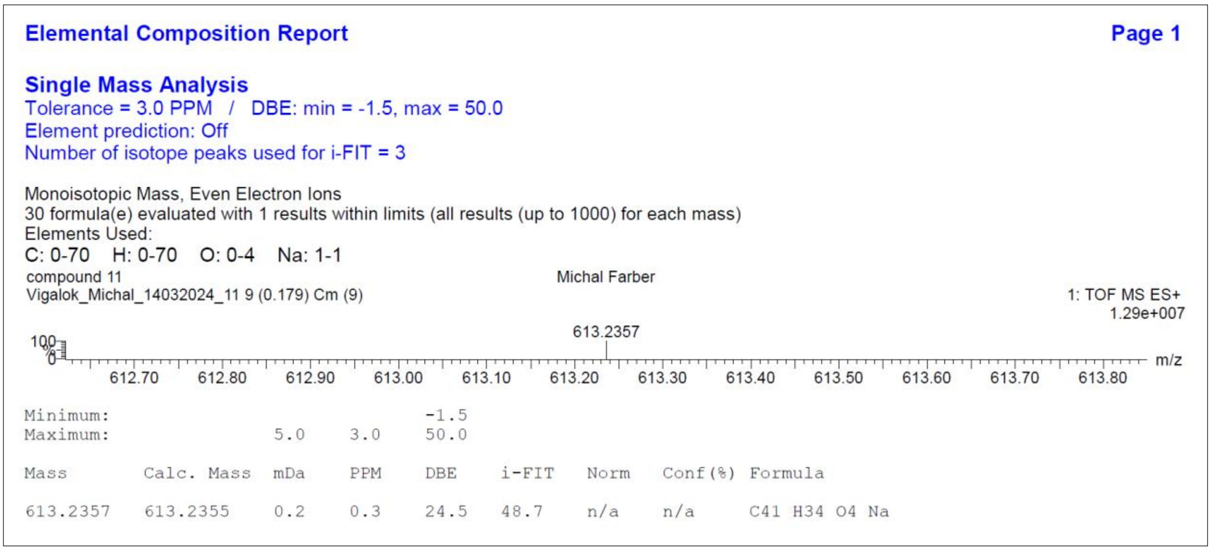

Elemental Composition Report — Page 1

Single Mass Analysis
Tolerance = 3.0 PPM / DBE: min = -1.5, max = 50.0
Element prediction: Off
Number of isotope peaks used for i-FIT = 3

Monoisotopic Mass, Even Electron Ions
30 formula(e) evaluated with 1 results within limits (all results (up to 1000) for each mass)
Elements Used:
C: 0-70 H: 0-70 O: 0-4 Na: 1-1
compound 11 — Michal Farber
Vigalok_Michal_14032024_11 9 (0.179) Cm (9) — 1: TOF MS ES+ 1.29e+007

Minimum:				-1.5				
Maximum:		5.0	3.0	50.0				
Mass	Calc. Mass	mDa	PPM	DBE	i-FIT	Norm	Conf(%)	Formula
613.2357	613.2355	0.2	0.3	24.5	48.7	n/a	n/a	C41 H34 O4 Na

*Fig. S21 - Mass spectrum of **1***

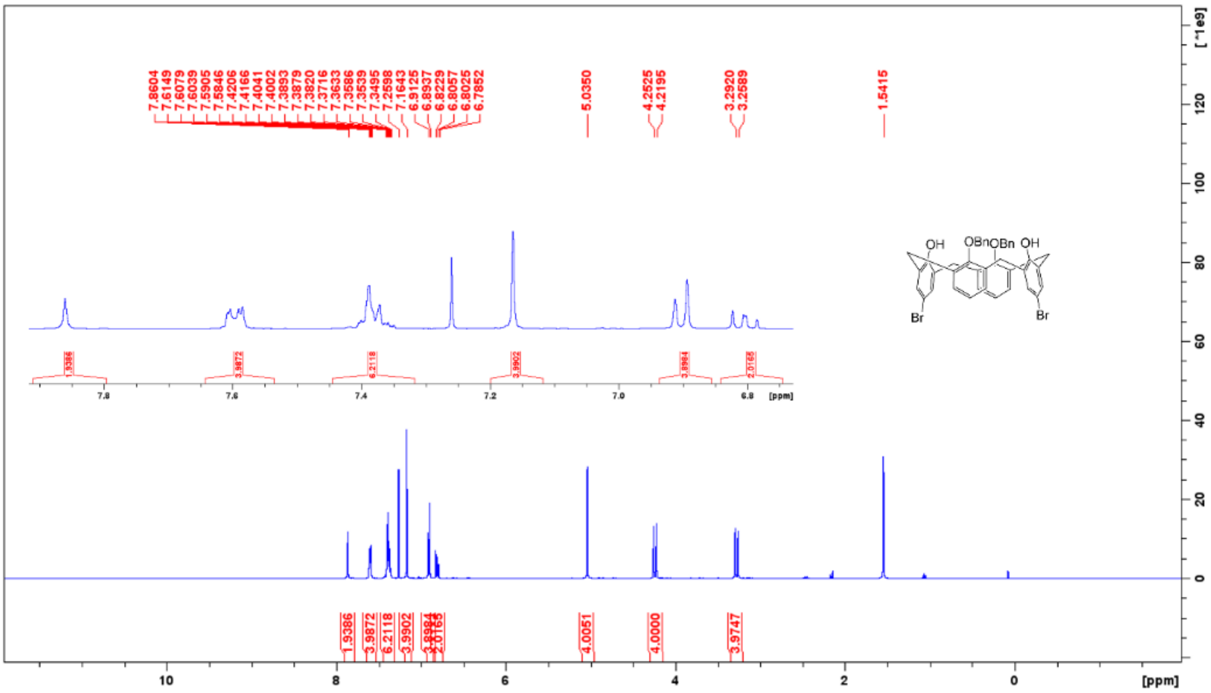

*Fig. S22 - ^{1}H NMR spectrum of **II*** (400 MHz, $CDCl_3$)

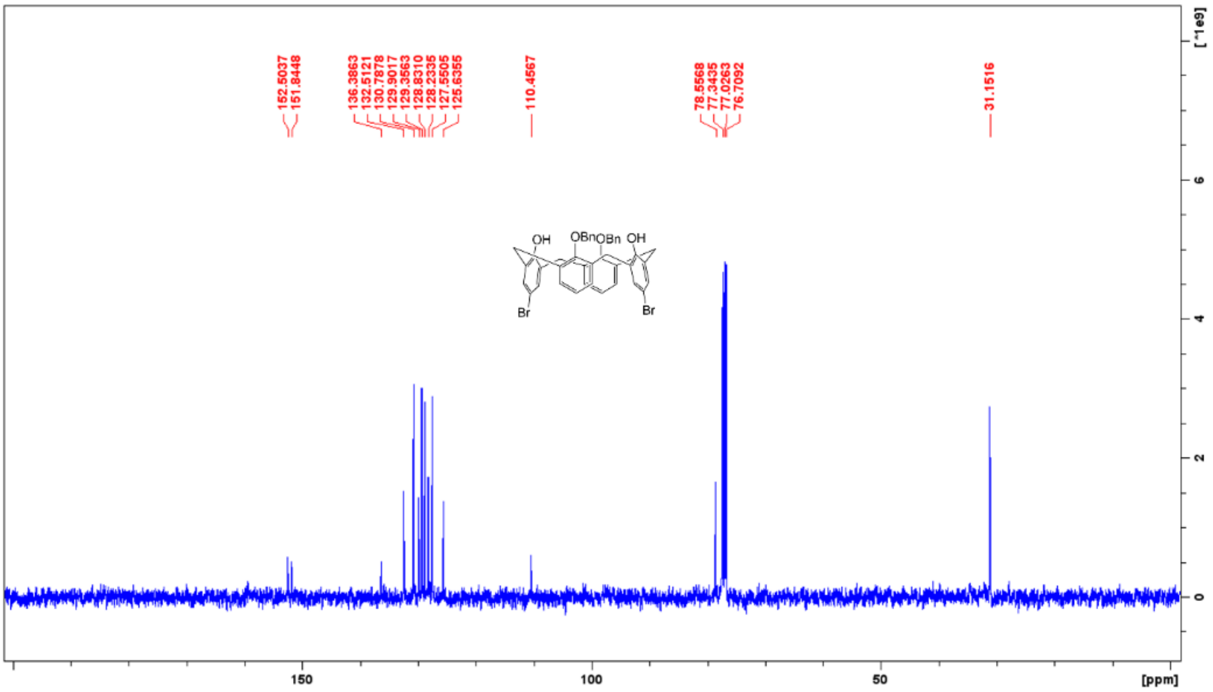

*Fig. S23 – $^{13}\{C\}^{1}H$ NMR spectrum of **II*** (100 MHz, $CDCl_3$)

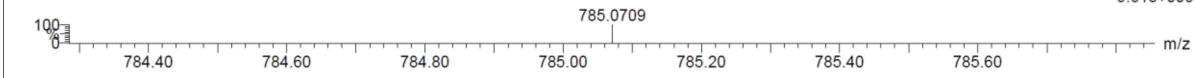

Elemental Composition Report Page 1

Single Mass Analysis
Tolerance = 3.0 PPM / DBE: min = -1.5, max = 50.0
Element prediction: Off
Number of isotope peaks used for i-FIT = 3

Monoisotopic Mass, Even Electron Ions
117 formula(e) evaluated with 1 results within limits (all results (up to 1000) for each mass)
Elements Used:
C: 0-70 H: 0-70 O: 0-4 Na: 1-1 79Br: 1-2 81Br: 1-2

compound 12 Michal Farber
Vigalok_Michal_14032024_12 8 (0.161) Cm (8:10) 1: TOF MS ES+ 9.61e+006

	Mass	Calc. Mass	mDa	PPM	DBE	i-FIT	Norm	Conf(%)	Formula
Minimum:					-1.5				
Maximum:			5.0	3.0	50.0				
	785.0709	785.0701	0.8	1.0	24.5	48.5	n/a	n/a	C42 H34 O4 Na 79Br 81Br

*Fig. S24 - Mass spectrum of **II***

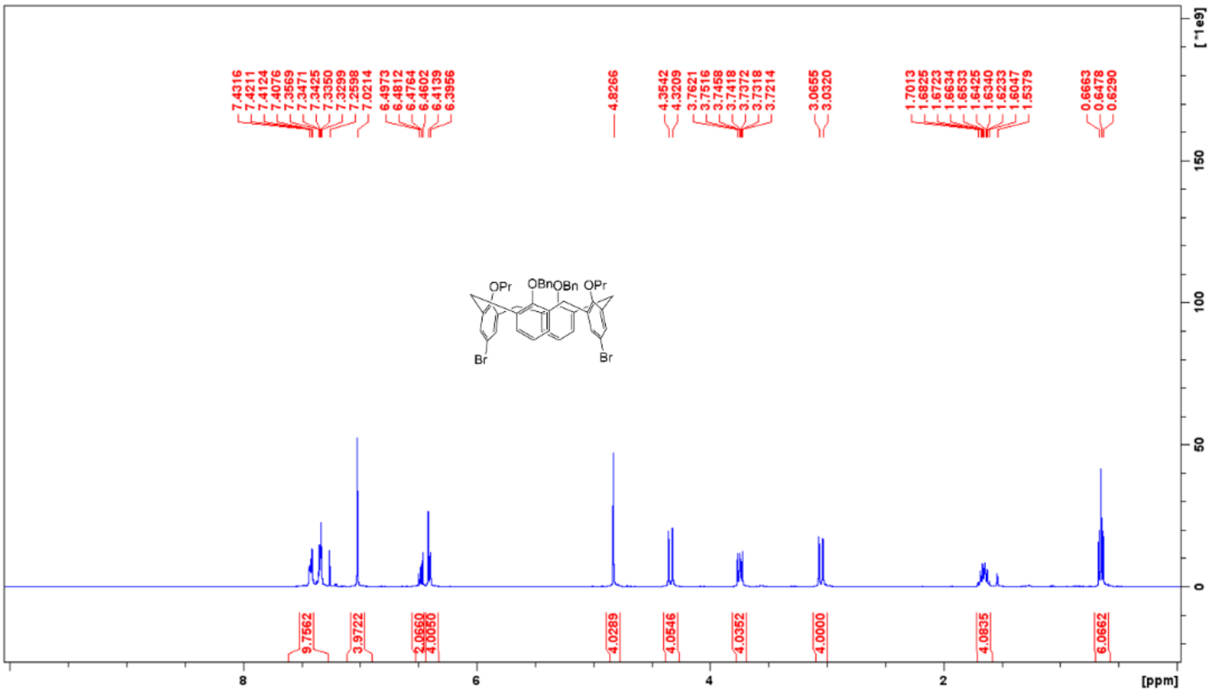

Fig. S25 - ^{1}H NMR spectrum of ***III*** (400 MHz, $CDCl_3$)

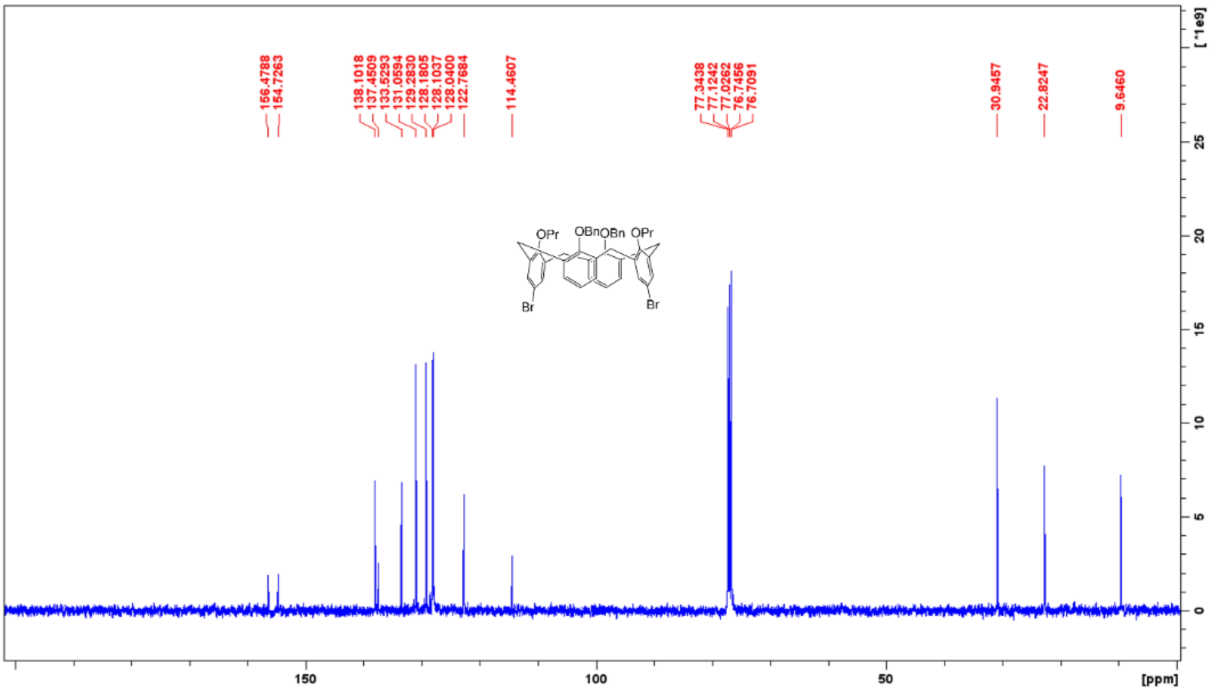

Fig. S26 – ^{13}{C}^{1}H NMR spectrum of ***III*** (100 MHz, $CDCl_3$)

Elemental Composition Report

Page 1

Single Mass Analysis

Tolerance = 3.0 PPM / DBE: min = -1.5, max = 50.0
Element prediction: Off
Number of isotope peaks used for i-FIT = 3

Monoisotopic Mass, Even Electron Ions
122 formula(e) evaluated with 1 results within limits (all results (up to 1000) for each mass)
Elements Used:
C: 0-70 H: 0-70 O: 0-4 Na: 1-1 79Br: 1-2 81Br: 1-2

compound 13 Michal Farber
Vigalok_Michal_14032024_13 10 (0.196) Cm (10:13) 1: TOF MS ES+ 7.08e+007

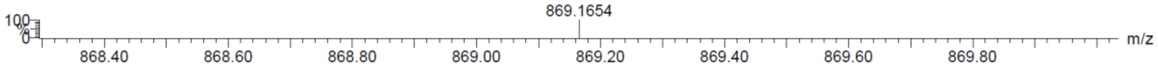

	Mass	Calc. Mass	mDa	PPM	DBE	i-FIT	Norm	Conf(%)	Formula
Minimum:					-1.5				
Maximum:			5.0	3.0	50.0				
	869.1654	869.1640	1.4	1.6	24.5	53.6	n/a	n/a	C48 H46 O4 Na 79Br 81Br

*Fig. S27 - Mass spectrum of **III***

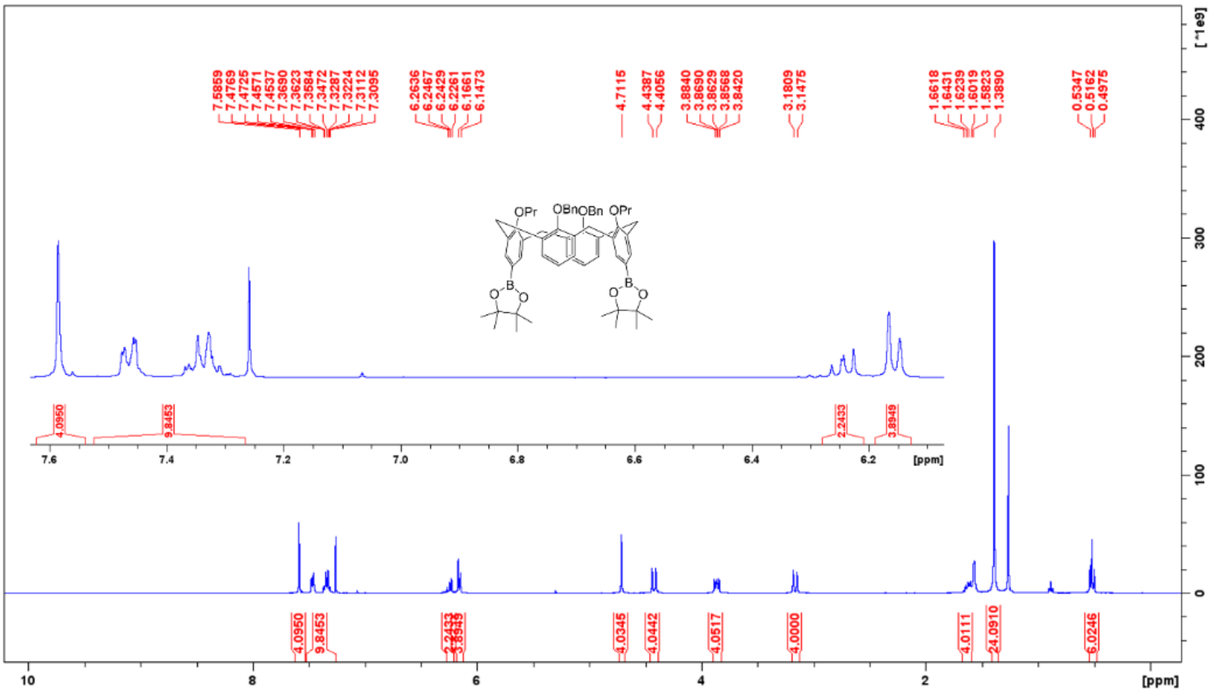

*Fig. S28 - ^{1}H NMR spectrum of **8*** (400 MHz, $CDCl_3$)

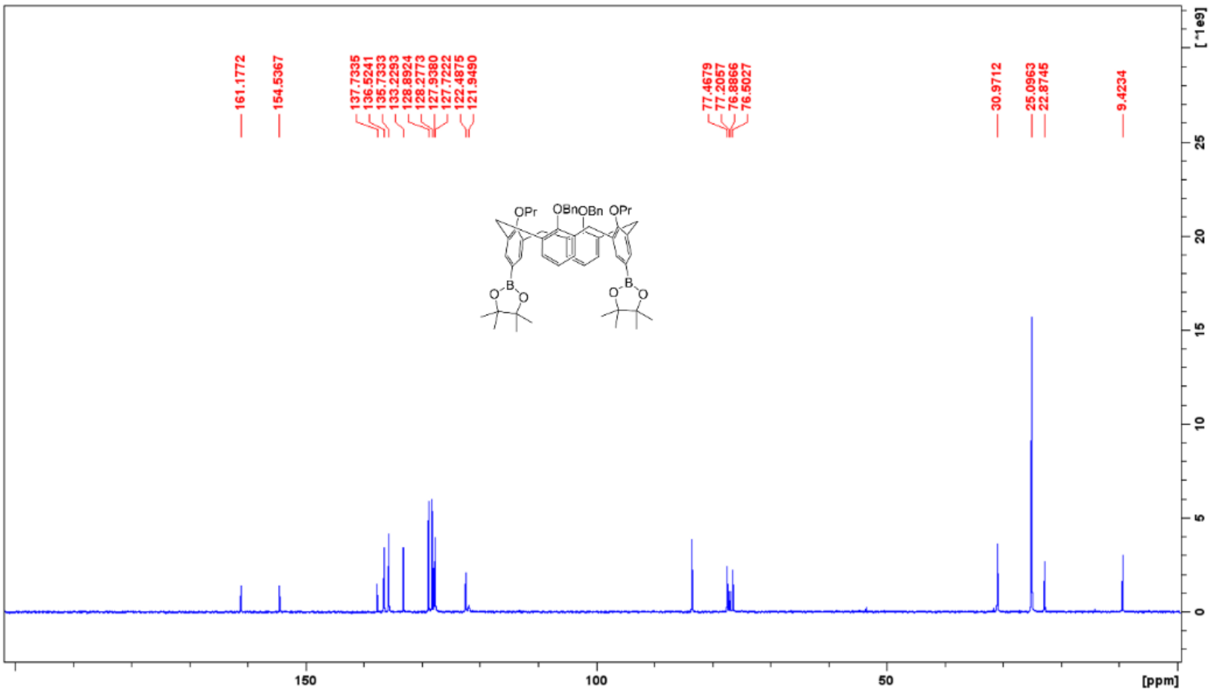

*Fig. S29 - $^{13}C\{^1H\}$ NMR spectrum of **8*** (100 MHz, $CDCl_3$)

Elemental Composition Report **Page 1**

Single Mass Analysis
Tolerance = 3.0 PPM / DBE: min = -1.5, max = 50.0
Element prediction: Off
Number of isotope peaks used for i-FIT = 3

Monoisotopic Mass, Even Electron Ions
101 formula(e) evaluated with 1 results within limits (all results (up to 1000) for each mass)
Elements Used:
C: 0-70 H: 0-70 O: 0-8 Na: 1-1 B: 0-2

compound 14 Michal Farber
Vigalok_Michal_14032024_14 10 (0.196) Cm (10:11) 1: TOF MS ES+
8.48e+007

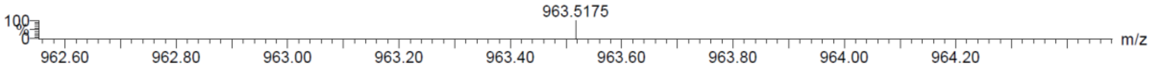

Minimum:				-1.5				
Maximum:		5.0	3.0	50.0				
Mass	Calc. Mass	mDa	PPM	DBE	i-FIT	Norm	Conf(%)	Formula
963.5175	963.5154	2.1	2.2	26.5	8.6	n/a	n/a	C60 H70 O8 Na B2

*Fig. S30 - Mass spectrum of **8***

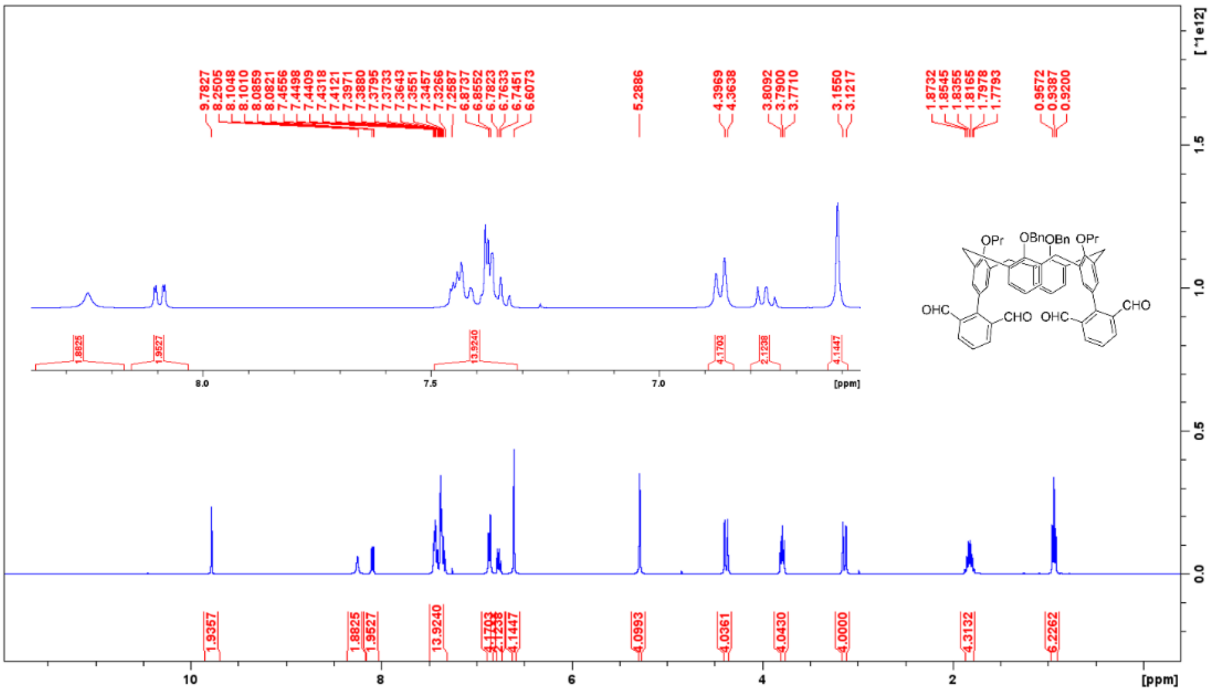

*Fig. S31 - ^{1}H NMR spectrum of **9*** (400 MHz, $CDCl_3$)

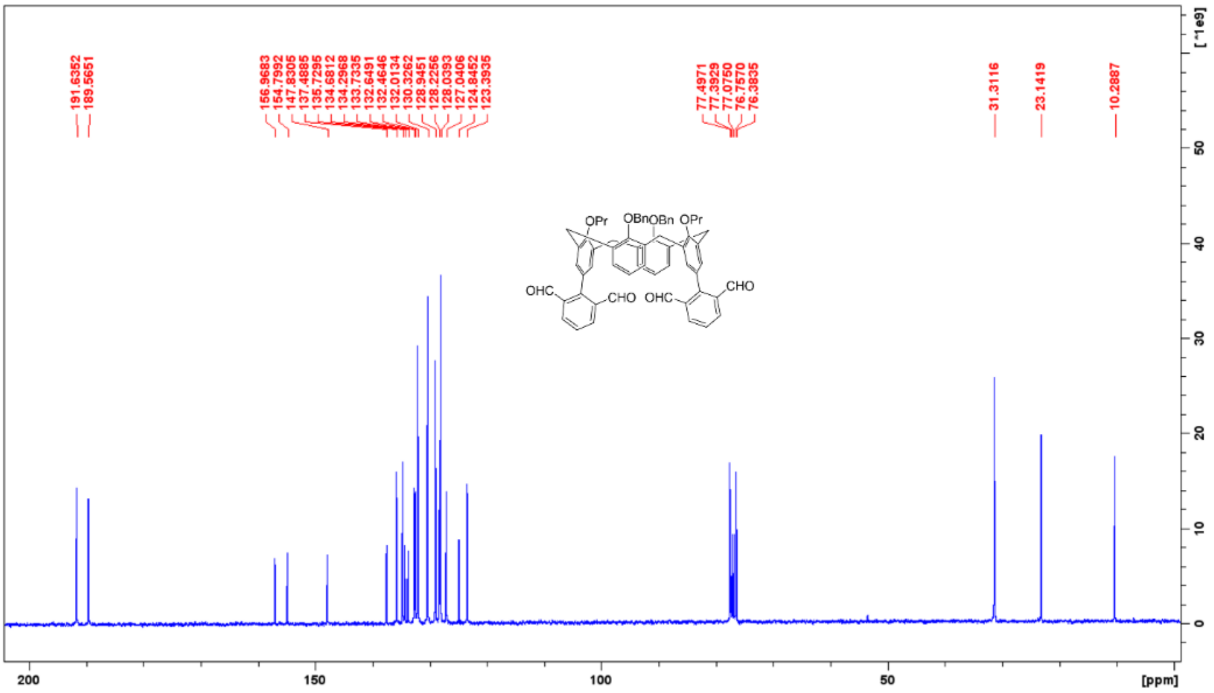

*Fig. S32 - ^{13}C{^{1}H} NMR spectrum of **9*** (100 MHz, $CDCl_3$)

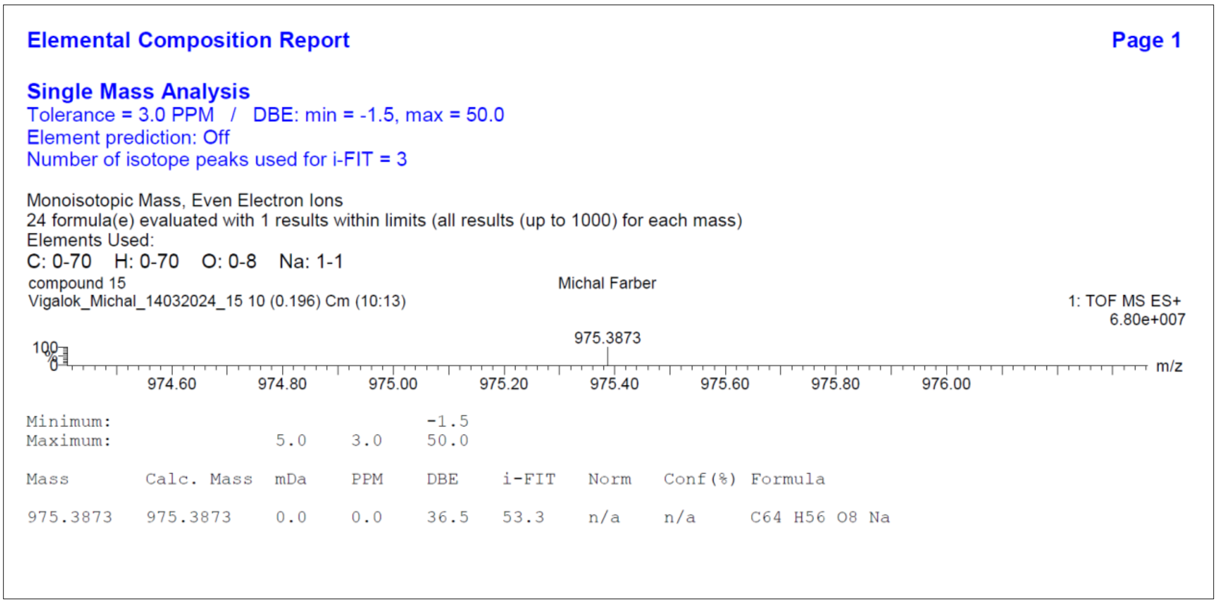

Elemental Composition Report **Page 1**

Single Mass Analysis
Tolerance = 3.0 PPM / DBE: min = -1.5, max = 50.0
Element prediction: Off
Number of isotope peaks used for i-FIT = 3

Monoisotopic Mass, Even Electron Ions
24 formula(e) evaluated with 1 results within limits (all results (up to 1000) for each mass)
Elements Used:
C: 0-70 H: 0-70 O: 0-8 Na: 1-1
compound 15 Michal Farber
Vigalok_Michal_14032024_15 10 (0.196) Cm (10:13) 1: TOF MS ES+ 6.80e+007

Minimum:				-1.5				
Maximum:		5.0	3.0	50.0				
Mass	Calc. Mass	mDa	PPM	DBE	i-FIT	Norm	Conf(%)	Formula
975.3873	975.3873	0.0	0.0	36.5	53.3	n/a	n/a	C64 H56 O8 Na

*Fig. S33 - Mass spectrum of **9***

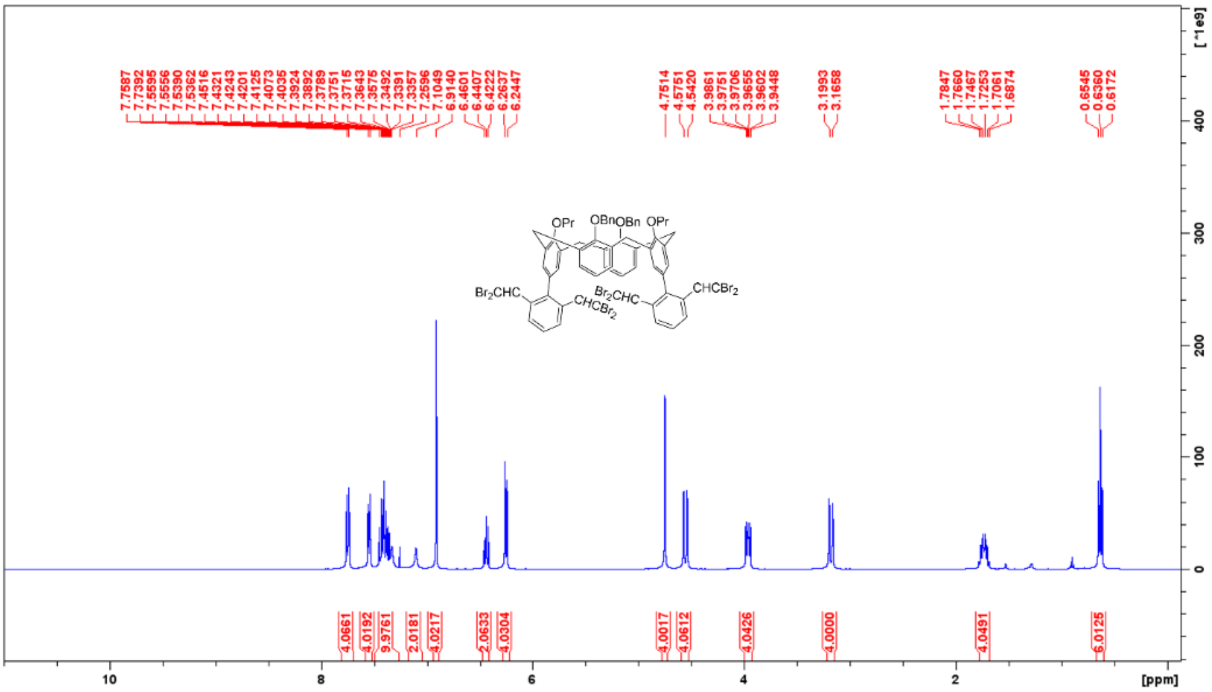

*Fig. S34 - ^{1}H NMR spectrum of **10a*** (400 MHz, $CDCl_3$)

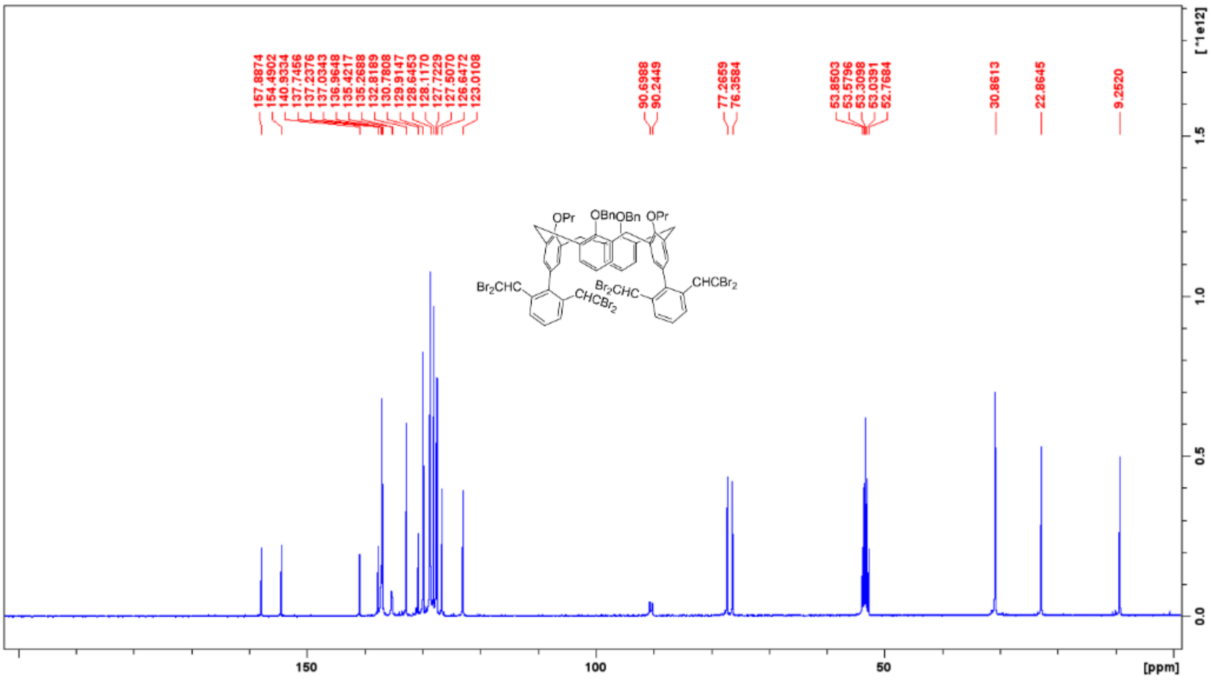

*Fig. S35 - $^{13}C\{^1H\}$ NMR spectrum of **10a*** (100 MHz, CD_2Cl_2)

Elemental Composition Report **Page 1**

Single Mass Analysis
Tolerance = 3.0 PPM / DBE: min = -1.5, max = 50.0
Element prediction: Off
Number of isotope peaks used for i-FIT = 3

Monoisotopic Mass, Even Electron Ions
42 formula(e) evaluated with 1 results within limits (all results (up to 1000) for each mass)
Elements Used:
C: 0-70 H: 0-70 O: 0-8 Na: 1-1 79Br: 2-4 81Br: 4-4

compound 16 Michal Farber
Vigalok_Michal_14032024_16 10 (0.196) Cm (10:12) 1: TOF MS ES+
4.86e+006

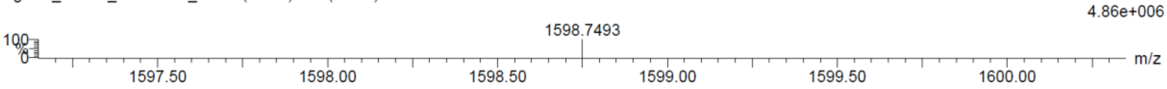

Minimum:				-1.5				
Maximum:		5.0	3.0	50.0				
Mass	Calc. Mass	mDa	PPM	DBE	i-FIT	Norm	Conf(%)	Formula
1598.7493	1598.7461	3.2	2.0	36.5	48.0	n/a	n/a	C68 H56 O4 Na 79Br4 81Br4

*Fig. S36 - Mass spectrum of **10a***

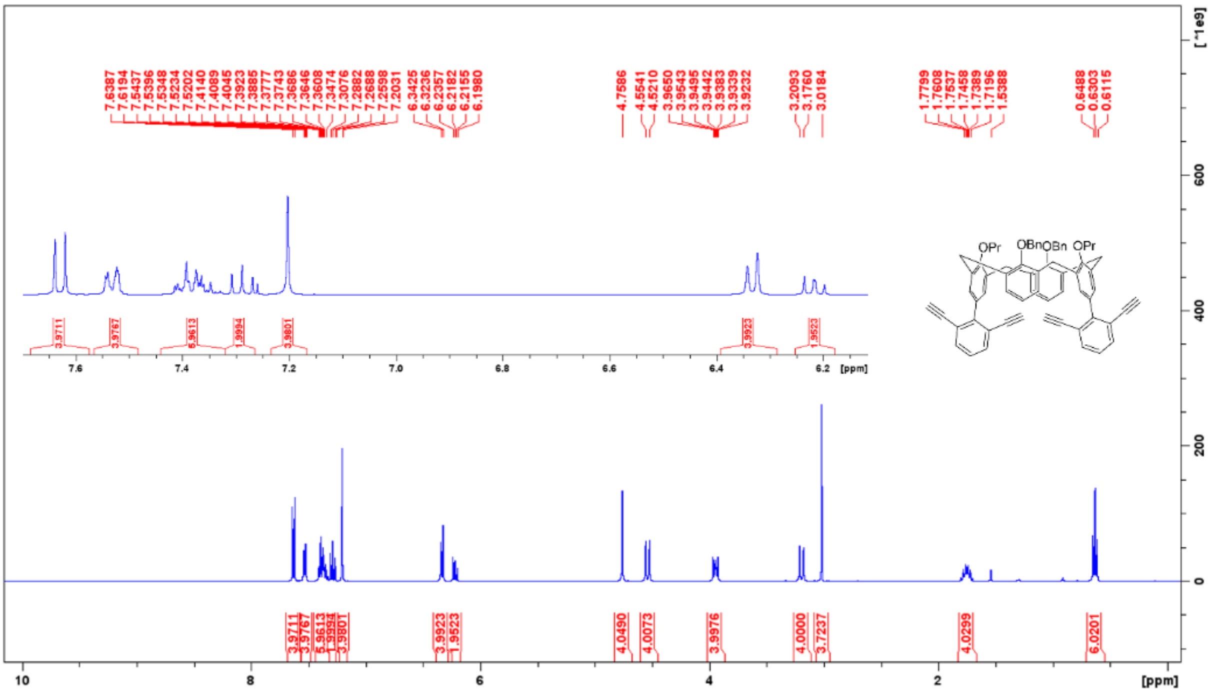

Fig. S37 - ^{1}H NMR spectrum of ***10*** (400 MHz, CDCl$_3$)

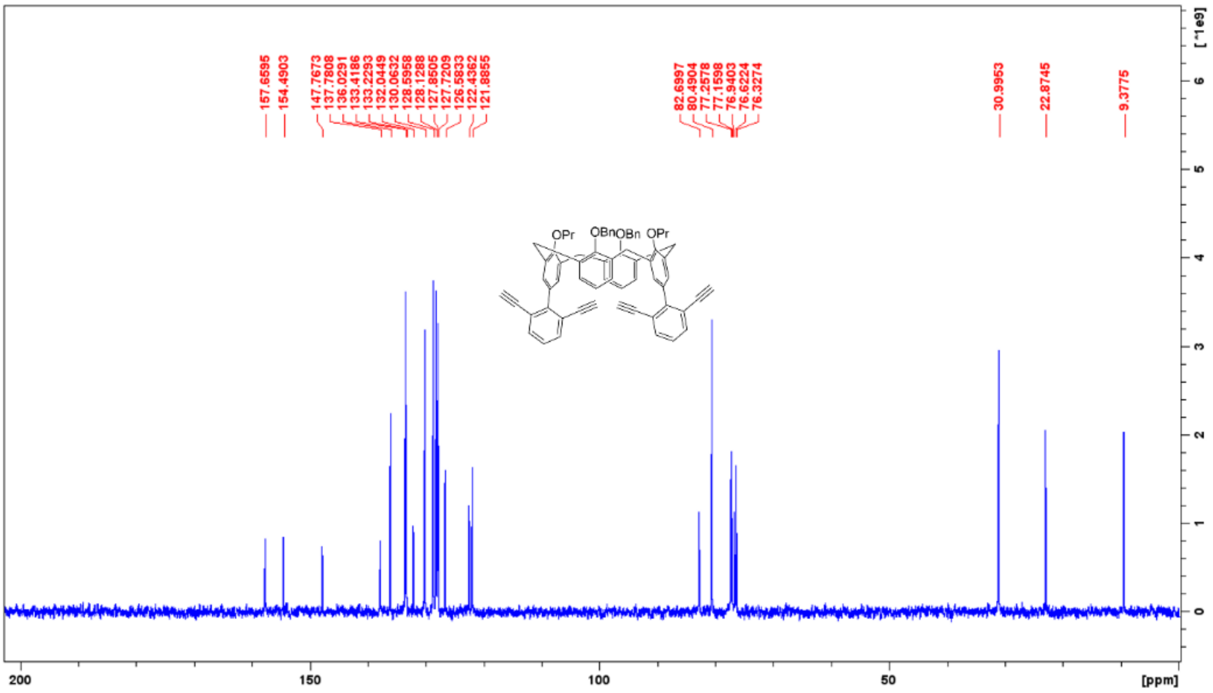

Fig. S38 - $^{13}C\{^1H\}$ NMR spectrum of ***10*** *(100 MHz, CDCl$_3$)*

Elemental Composition Report **Page 1**

Single Mass Analysis
Tolerance = 3.0 PPM / DBE: min = -1.5, max = 50.0
Element prediction: Off
Number of isotope peaks used for i-FIT = 3

Monoisotopic Mass, Even Electron Ions
30 formula(e) evaluated with 1 results within limits (all results (up to 1000) for each mass)
Elements Used:
C: 0-70 H: 0-70 O: 0-8 Na: 1-1

compound 17 Michal Farber
Vigalok_Michal_14032024_17 9 (0.179) Cm (9:10) 1: TOF MS ES+ 1.14e+007

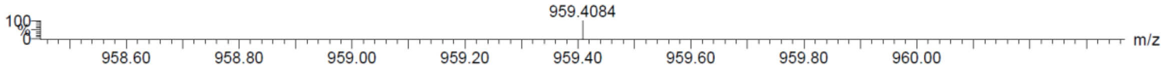

Mass	Calc. Mass	mDa	PPM	DBE	i-FIT	Norm	Conf(%)	Formula
Minimum:				-1.5				
Maximum:		5.0	3.0	50.0				
959.4084	959.4076	0.8	0.8	40.5	49.3	n/a	n/a	C68 H56 O4 Na

*Fig. S39 - Mass spectrum of **10***

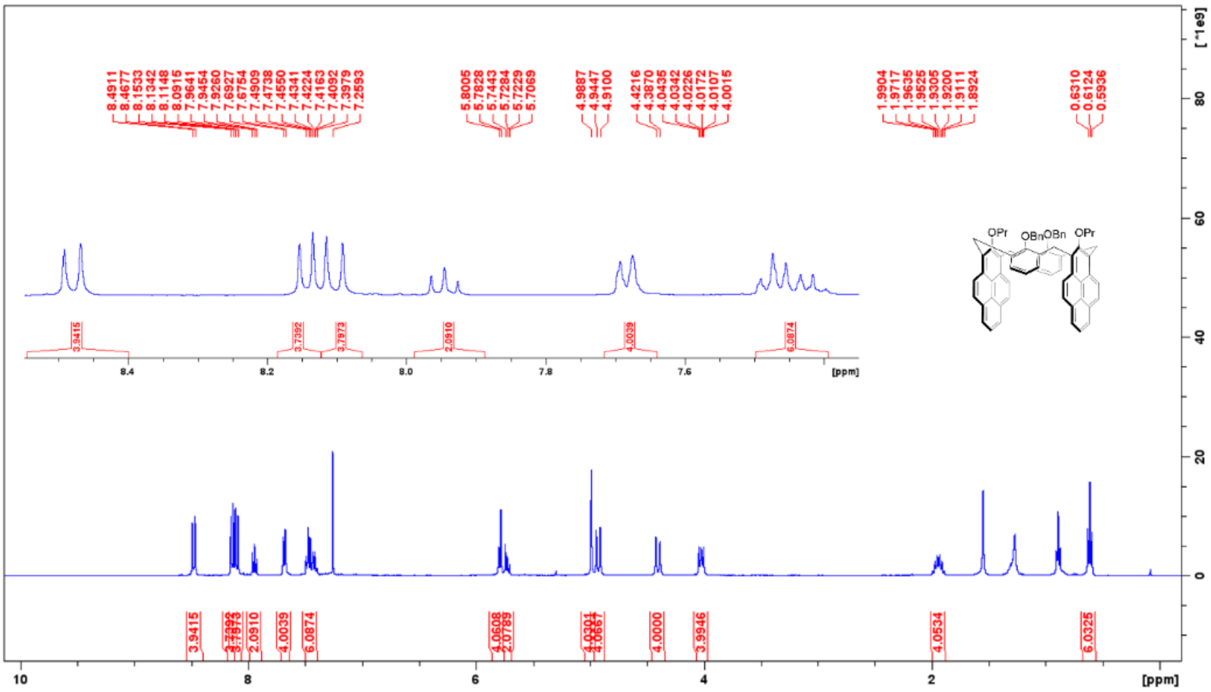

*Fig. S40 - ^{1}H NMR spectrum of **11*** (400 MHz, $CDCl_3$)

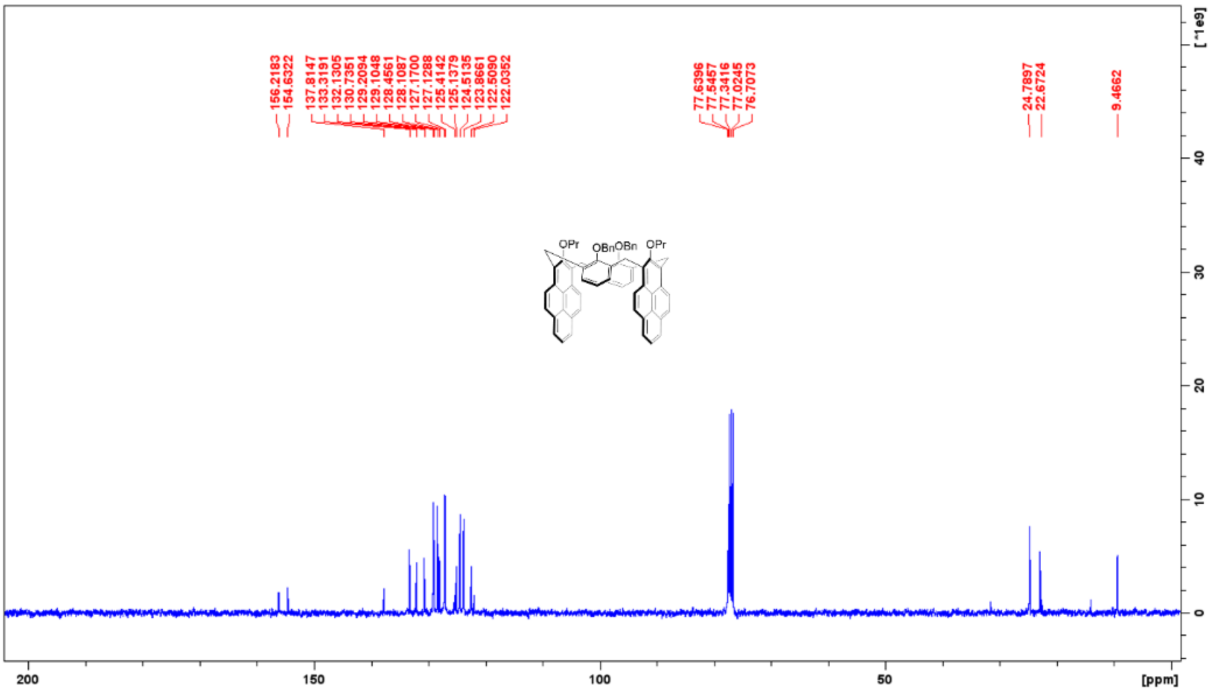

Fig. S41 - $^{13}C\{^1H\}$ NMR spectrum of ***11*** (100 MHz, $CDCl_3$)

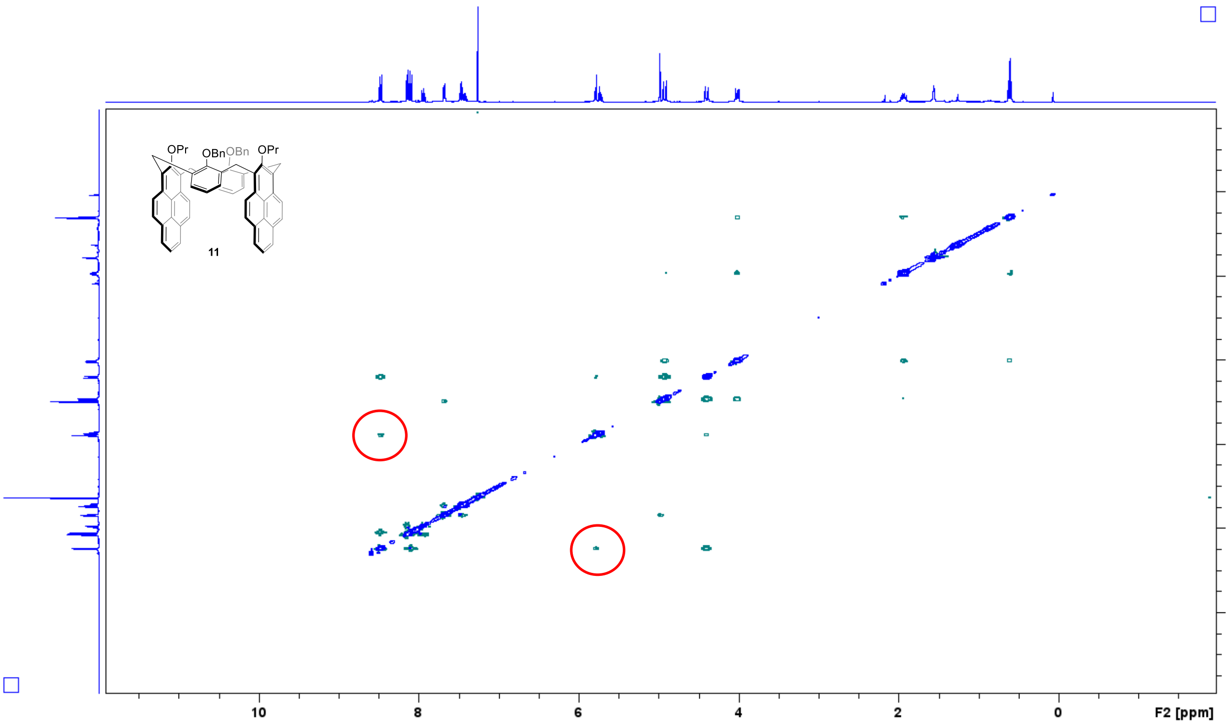

Fig. S42 - ^1H NMR NOESY spectrum of ***11*** (400 MHz, $CDCl_3$)

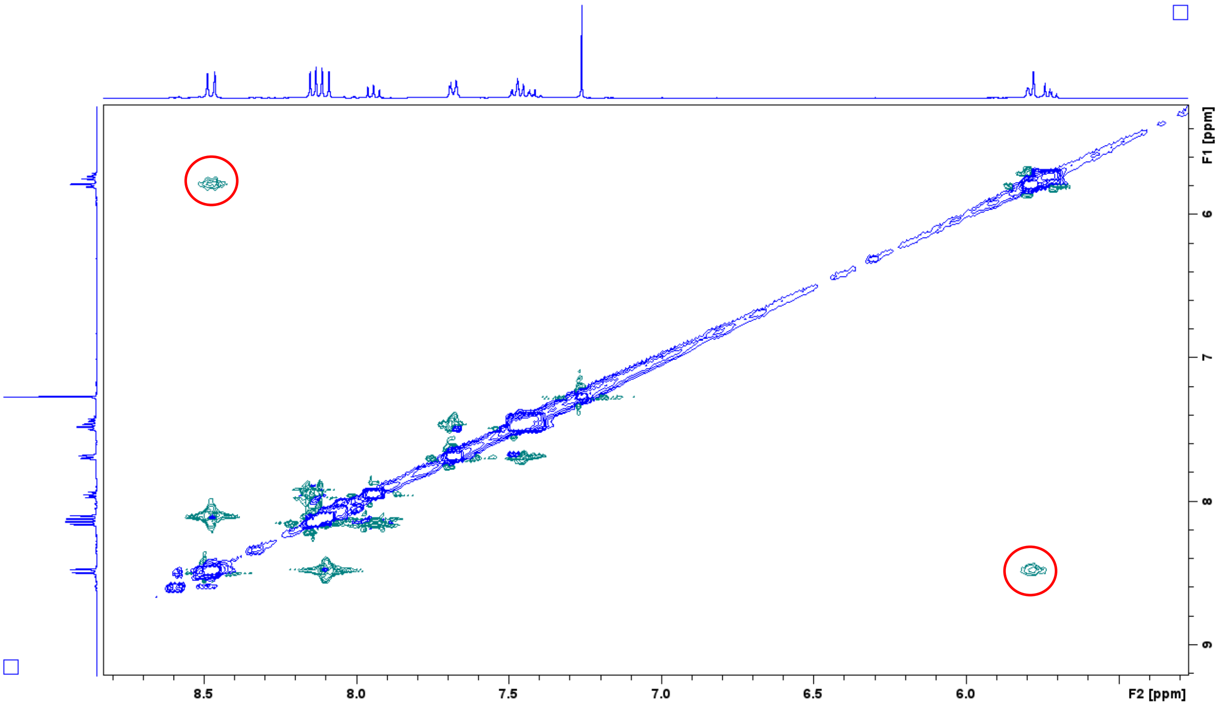

Fig. S43 – Aromatic region of the [1]*H NMR NOESY spectrum of* ***11*** (400 MHz, $CDCl_3$)

Elemental Composition Report **Page 1**

Single Mass Analysis
Tolerance = 3.0 PPM / DBE: min = -1.5, max = 50.0
Element prediction: Off
Number of isotope peaks used for i-FIT = 3

Monoisotopic Mass, Even Electron Ions
30 formula(e) evaluated with 1 results within limits (all results (up to 1000) for each mass)
Elements Used:
C: 0-70 H: 0-70 O: 0-8 Na: 1-1

compound 18 Michal Farber
Vigalok_Michal_14032024_18 9 (0.179) Cm (9:12) 1: TOF MS ES+
9.22e+007

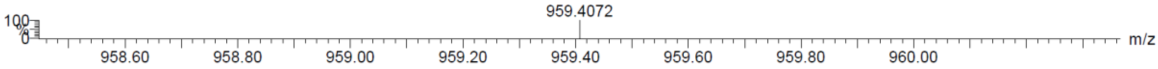

Minimum:				-1.5				
Maximum:		5.0	3.0	50.0				
Mass	Calc. Mass	mDa	PPM	DBE	i-FIT	Norm	Conf(%)	Formula
959.4072	959.4076	-0.4	-0.4	40.5	53.9	n/a	n/a	C68 H56 O4 Na

Fig. S44 - Mass spectrum of ***11***

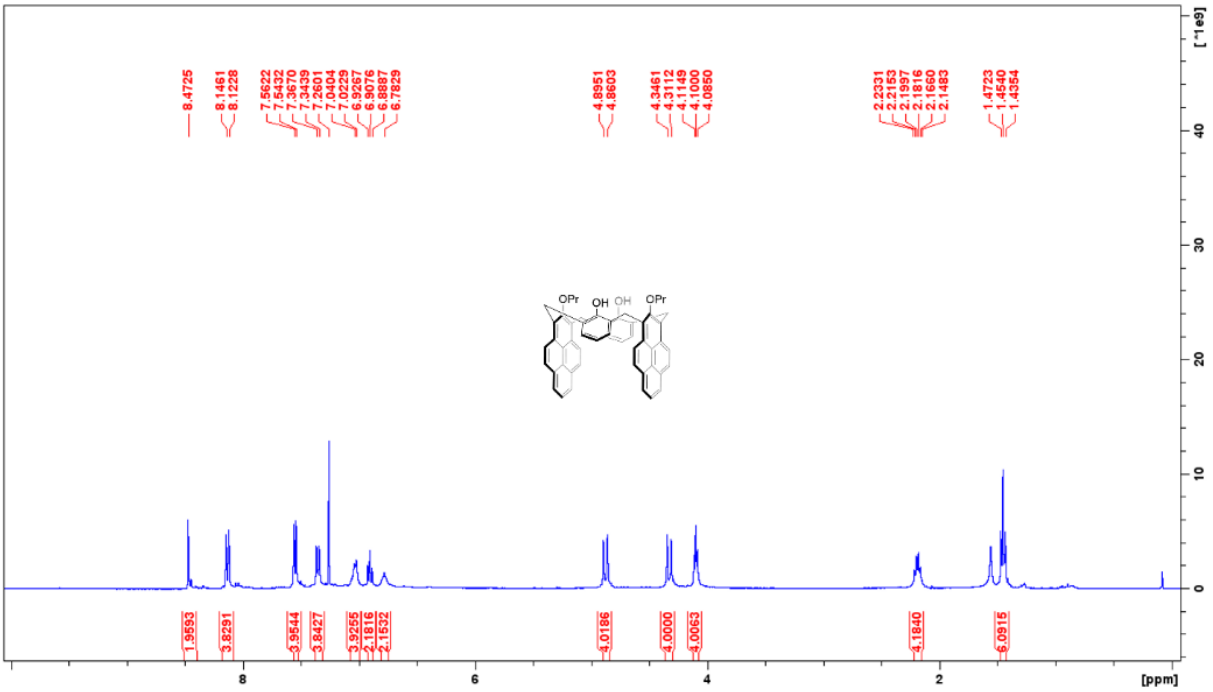

*Fig. S45 - ^{1}H NMR spectrum of **2*** (400 MHz, $CDCl_3$)

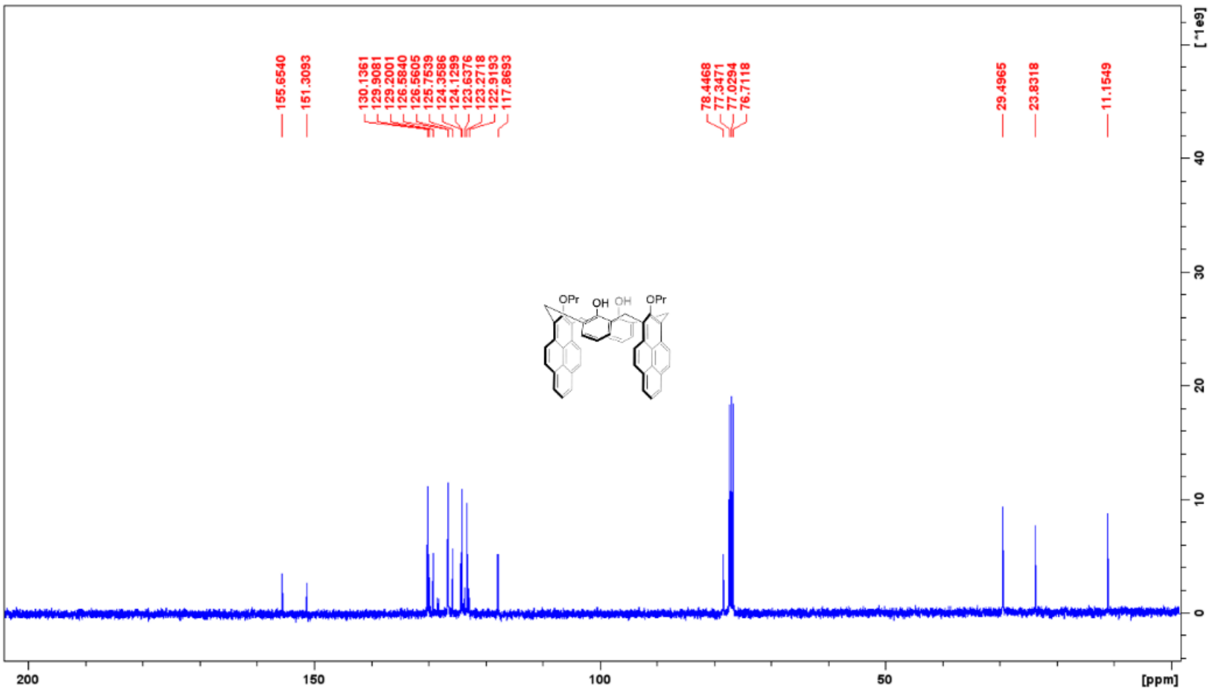

*Fig. S46 - $^{13}C\{^{1}H\}$ NMR spectrum of **2*** (100 MHz, $CDCl_3$)

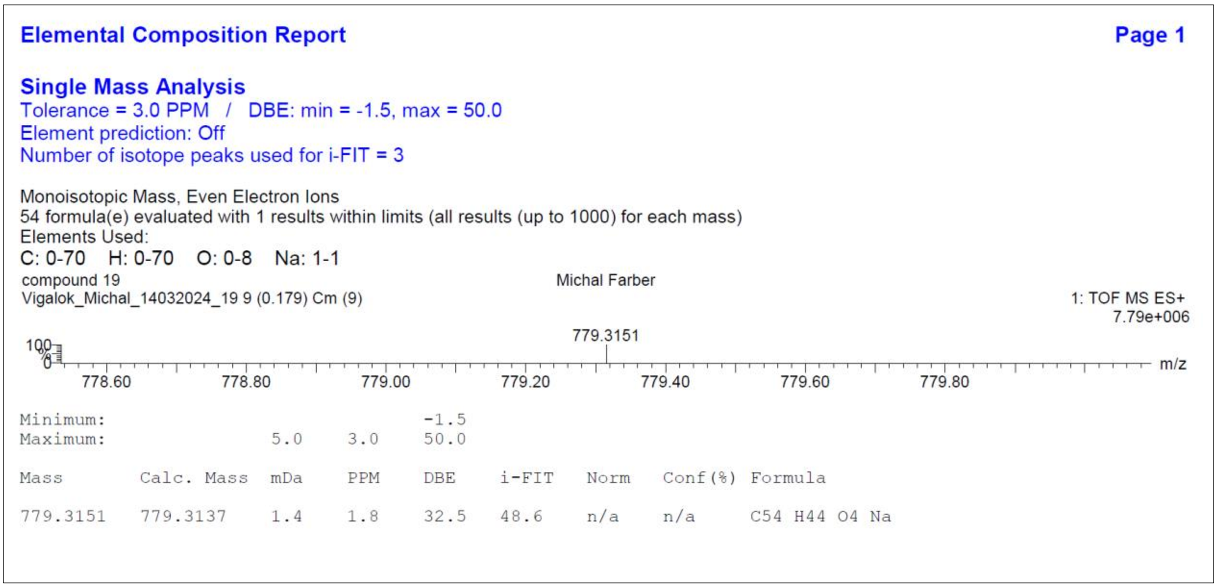

Fig. S47 - Mass spectrum of ***2***

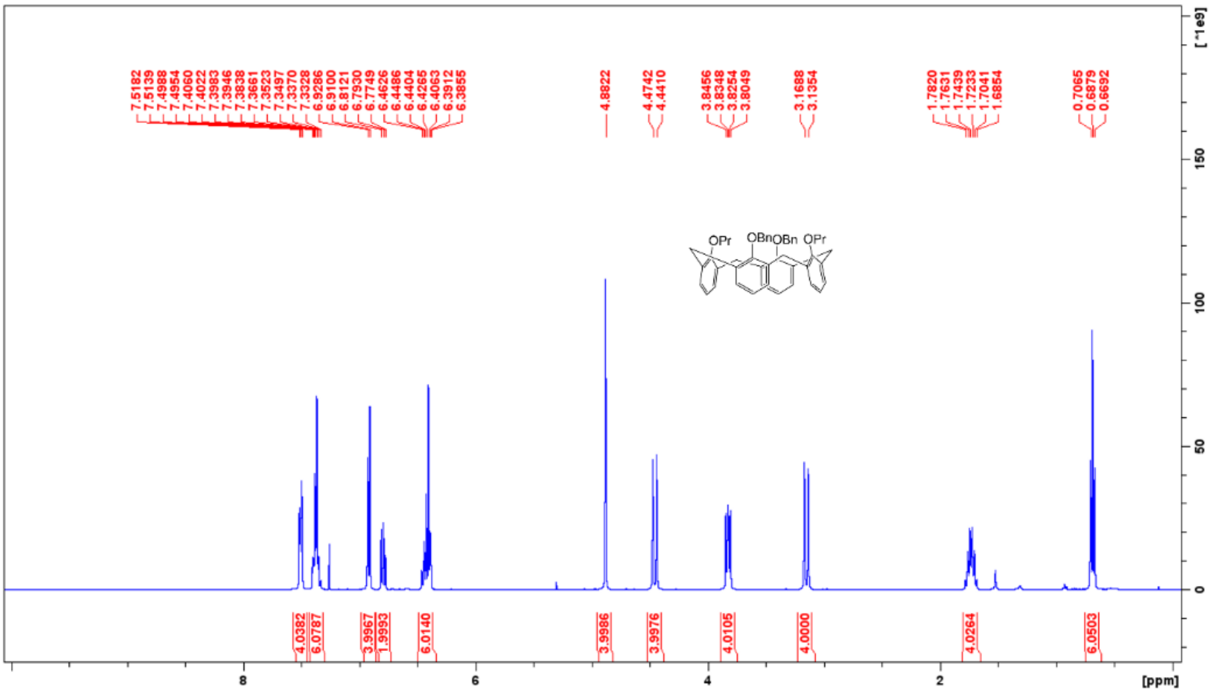

Fig. S48 - ^1H NMR spectrum of ***13*** (400 MHz, $CDCl_3$)

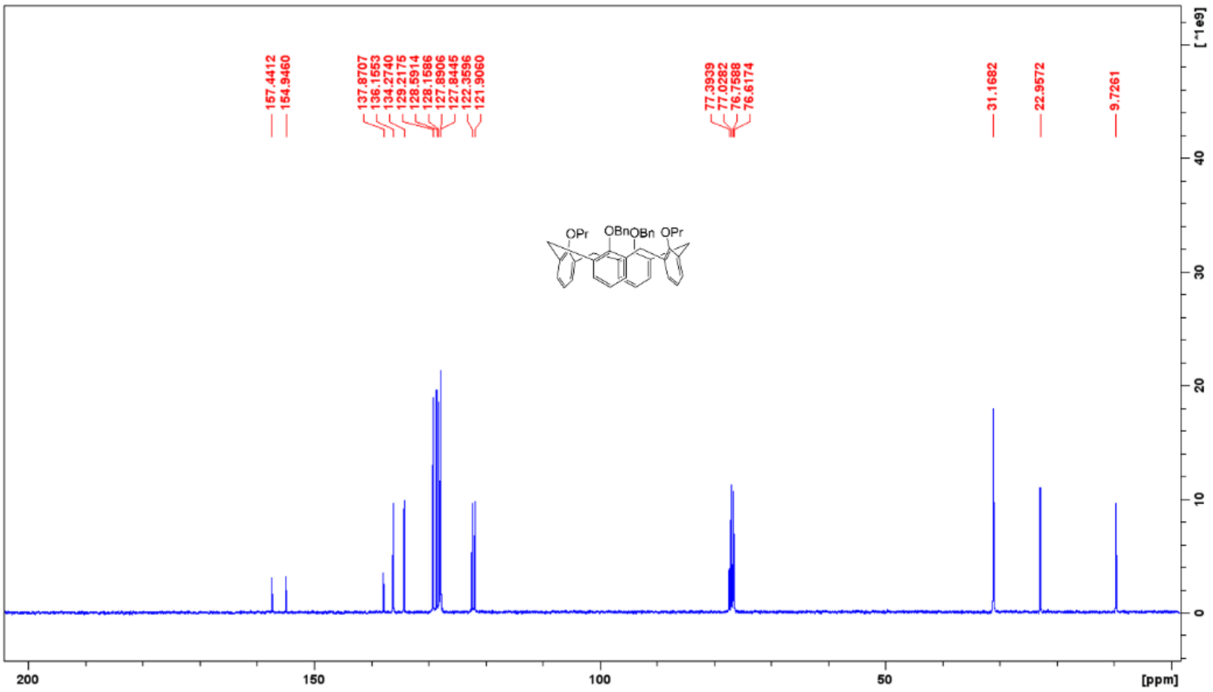

*Fig. S49 - $^{13}C\{^{1}H\}$ NMR spectrum of **13*** (100 MHz, $CDCl_3$)

Elemental Composition Report **Page 1**

Single Mass Analysis
Tolerance = 3.0 PPM / DBE: min = -1.5, max = 50.0
Element prediction: Off
Number of isotope peaks used for i-FIT = 3

Monoisotopic Mass, Even Electron Ions
54 formula(e) evaluated with 1 results within limits (all results (up to 1000) for each mass)
Elements Used:
C: 0-70 H: 0-70 O: 0-8 Na: 1-1
compound 20 Michal Farber
Vigalok_Michal_14032024_20 11 (0.221) Cm (11:13) 1: TOF MS ES+
4.22e+008

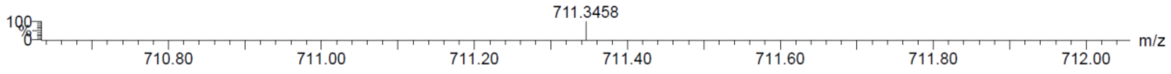

Minimum:				-1.5				
Maximum:		5.0	3.0	50.0				
Mass	Calc. Mass	mDa	PPM	DBE	i-FIT	Norm	Conf(%)	Formula
711.3458	711.3450	0.8	1.1	24.5	57.2	n/a	n/a	C48 H48 O4 Na

*Fig. S50 - Mass spectrum of **13***

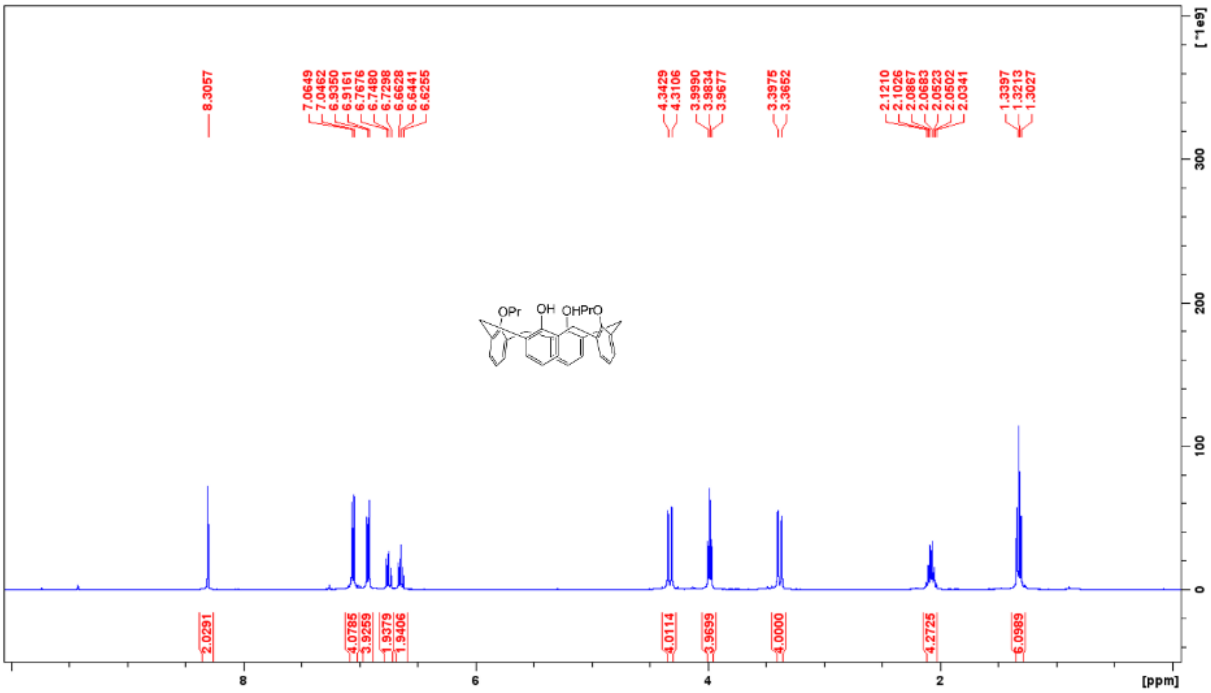

Fig. S51 - ^{1}H NMR spectrum of ***14*** (400 MHz, $CDCl_3$)

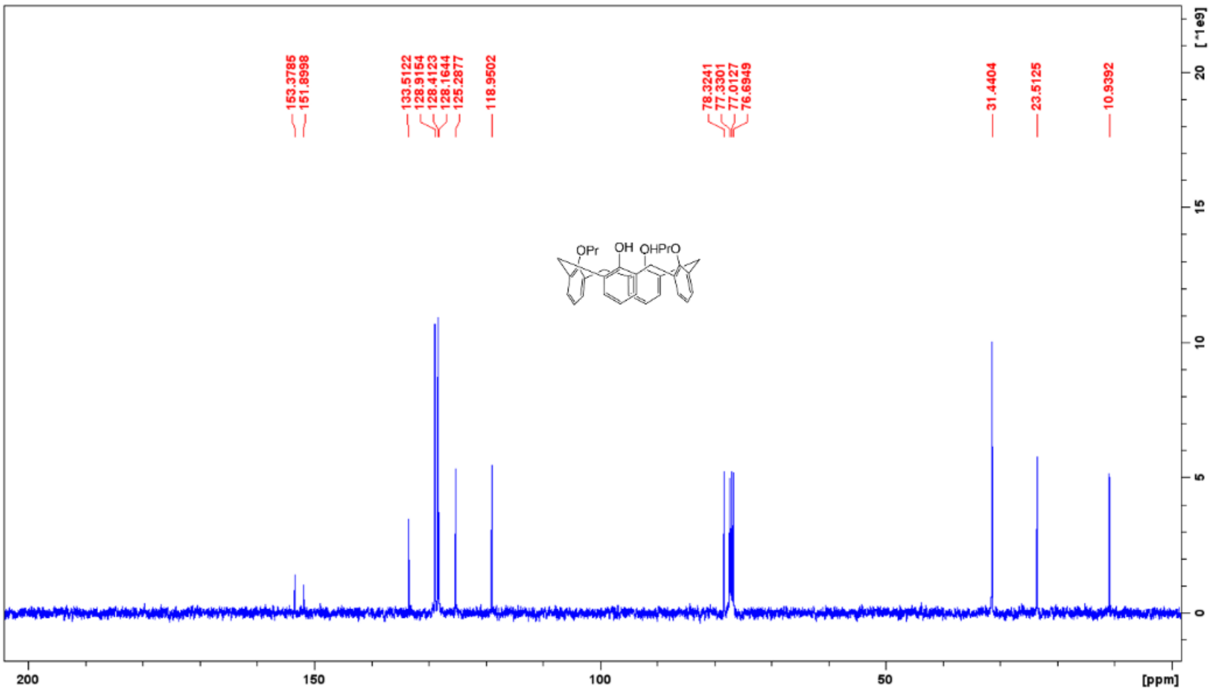

Fig. S52 - ^{13}C{^{1}H} NMR spectrum of ***14*** (100 MHz, $CDCl_3$)

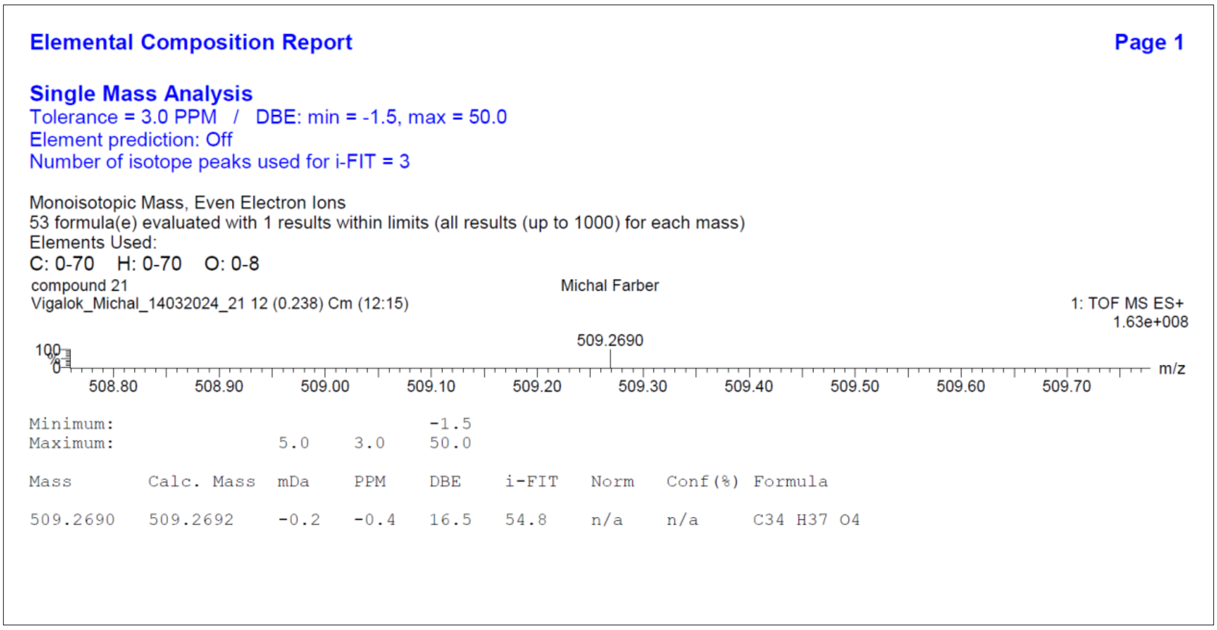

Elemental Composition Report Page 1

Single Mass Analysis
Tolerance = 3.0 PPM / DBE: min = -1.5, max = 50.0
Element prediction: Off
Number of isotope peaks used for i-FIT = 3

Monoisotopic Mass, Even Electron Ions
53 formula(e) evaluated with 1 results within limits (all results (up to 1000) for each mass)
Elements Used:
C: 0-70 H: 0-70 O: 0-8
compound 21 Michal Farber
Vigalok_Michal_14032024_21 12 (0.238) Cm (12:15) 1: TOF MS ES+ 1.63e+008

Minimum:				-1.5				
Maximum:		5.0	3.0	50.0				
Mass	Calc. Mass	mDa	PPM	DBE	i-FIT	Norm	Conf(%)	Formula
509.2690	509.2692	-0.2	-0.4	16.5	54.8	n/a	n/a	C34 H37 O4

Fig. S53 - Mass spectrum of ***14***

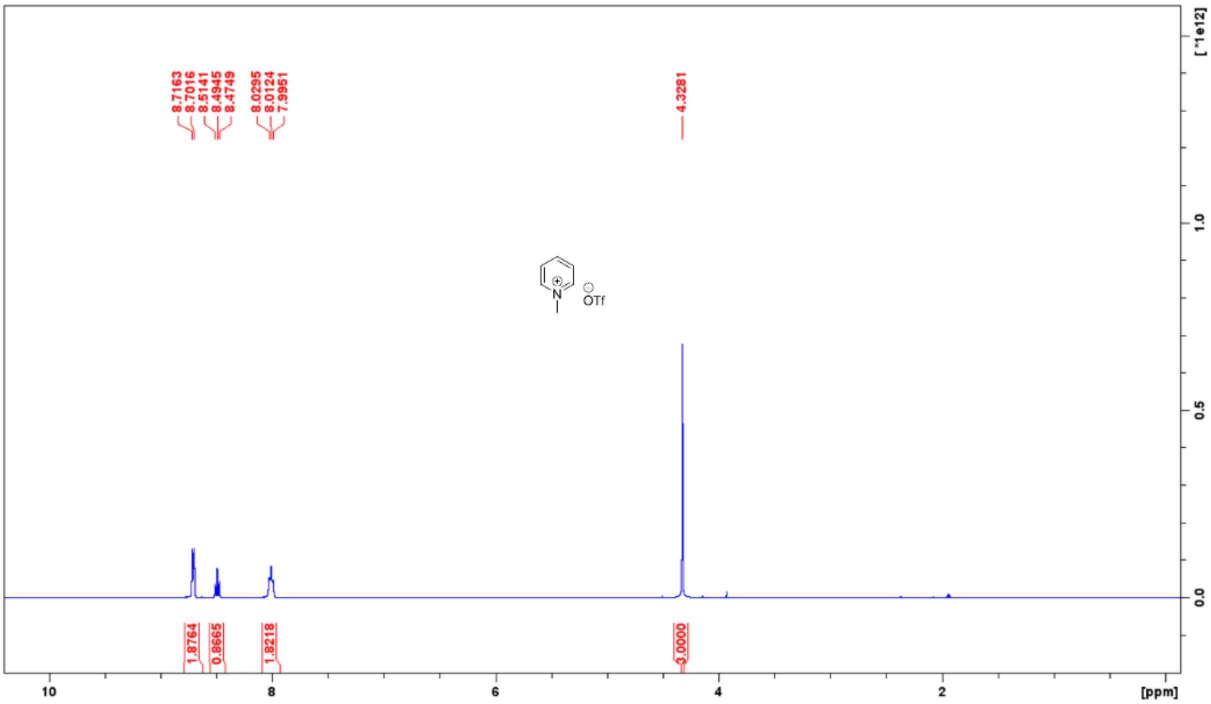

Fig. S54 - ^{1}H NMR spectrum of ***12*** (400 MHz, CD_3CN)

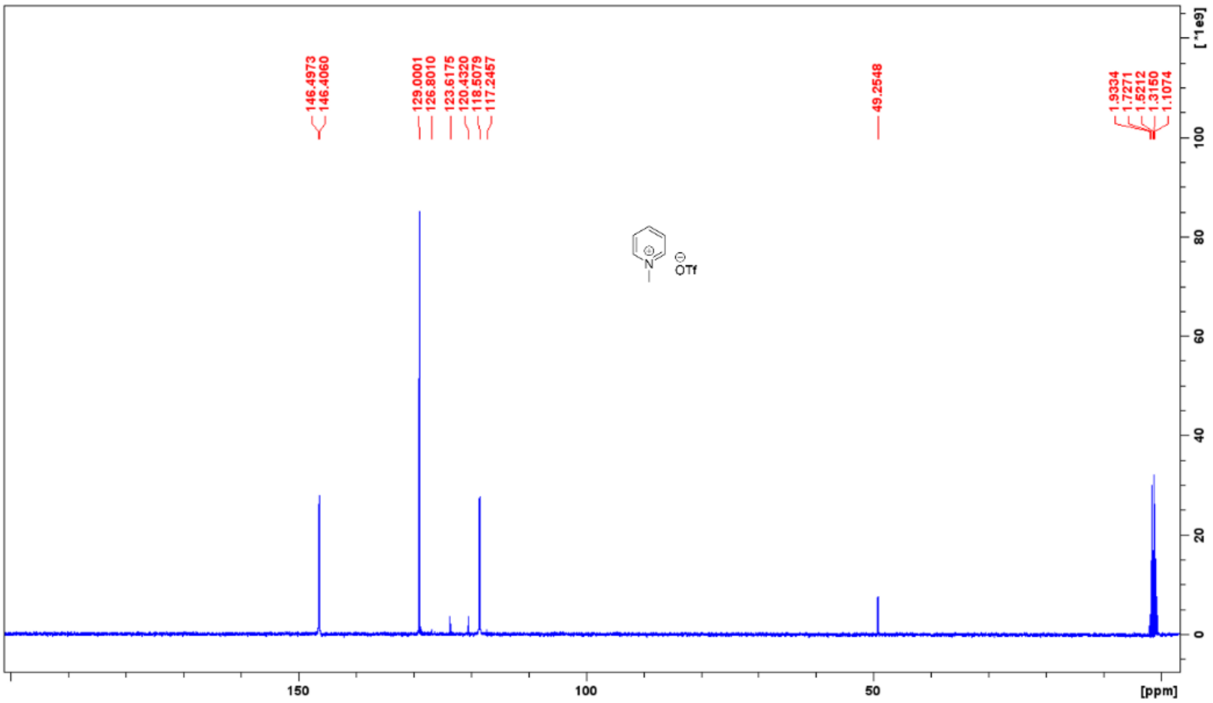

Fig. S55 - ^{13}C{^{1}H} NMR spectrum of ***12*** (100 MHz, CD_3CN)

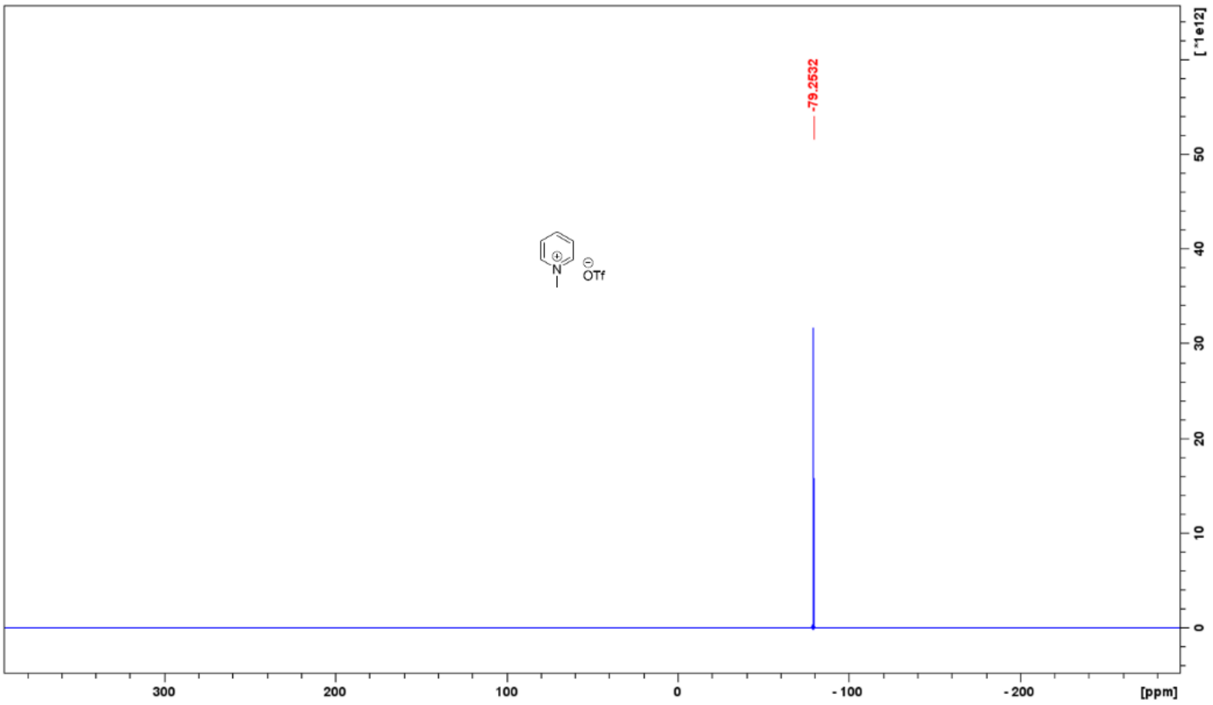

Fig. S56 – ^{19}F{^{1}H} NMR spectrum of ***12*** (376 MHz, CD_3CN)

Elemental Composition Report **Page 1**

Single Mass Analysis
Tolerance = 3.0 PPM / DBE: min = -1.5, max = 50.0
Element prediction: Off
Number of isotope peaks used for i-FIT = 3

Monoisotopic Mass, Even Electron Ions
3 formula(e) evaluated with 1 results within limits (all results (up to 1000) for each mass)
Elements Used:
C: 0-10 H: 0-10 N: 0-2
compound 22 Michal Farber
Vigalok_Michal_17032024_06 6 (0.127) Cm (6) 1: TOF MS ES+
3.18e+006

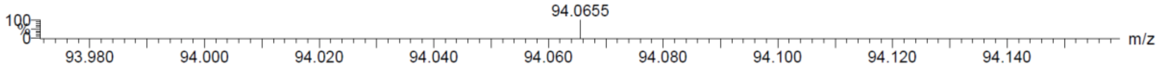

Minimum:				-1.5				
Maximum:		5.0	3.0	50.0				
Mass	Calc. Mass	mDa	PPM	DBE	i-FIT	Norm	Conf(%)	Formula
94.0655	94.0657	-0.2	-2.1	3.5	42.6	n/a	n/a	C6 H8 N

*Fig. S57 - Mass spectrum of **12***

4. Absorption and Emission Spectra

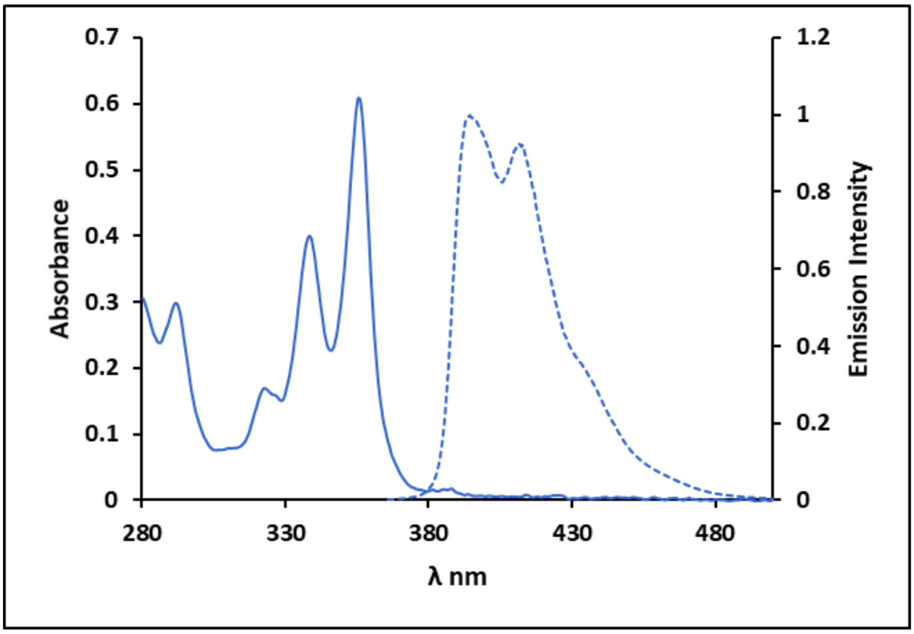

Fig. S58 –Absorption (solid line) and emission (dashed line) spectra of ***7***

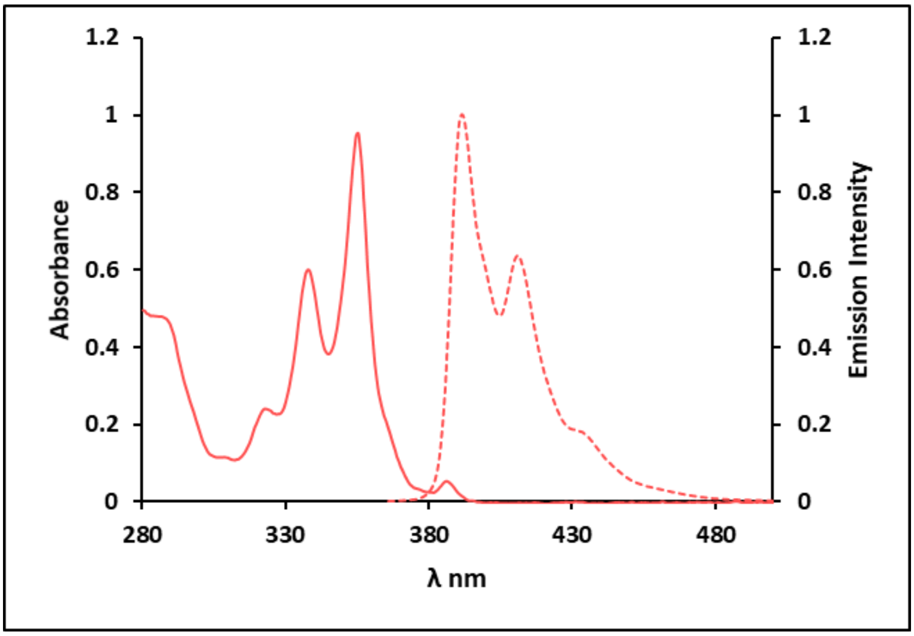

Fig. S59 –Absorption (solid line) and emission (dashed line) spectra of ***1***

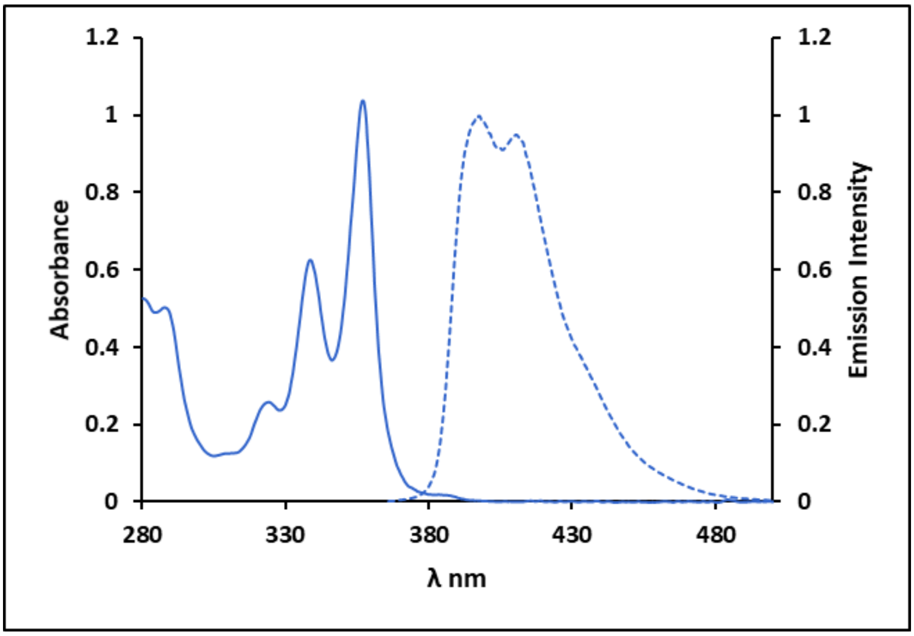

*Fig. S60 –Absorption (solid line) and emission (dashed line) spectra of **11***

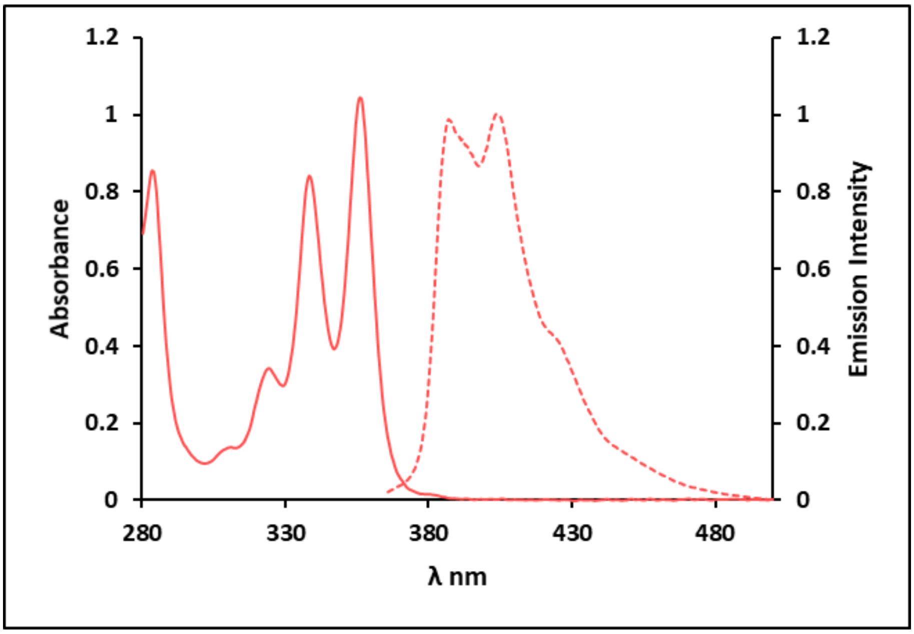

*Fig. S61 –Absorption (solid line) and emission (dashed line) spectra of **12***

5. Host-guest binding experiments

5.1 NMR Experiments

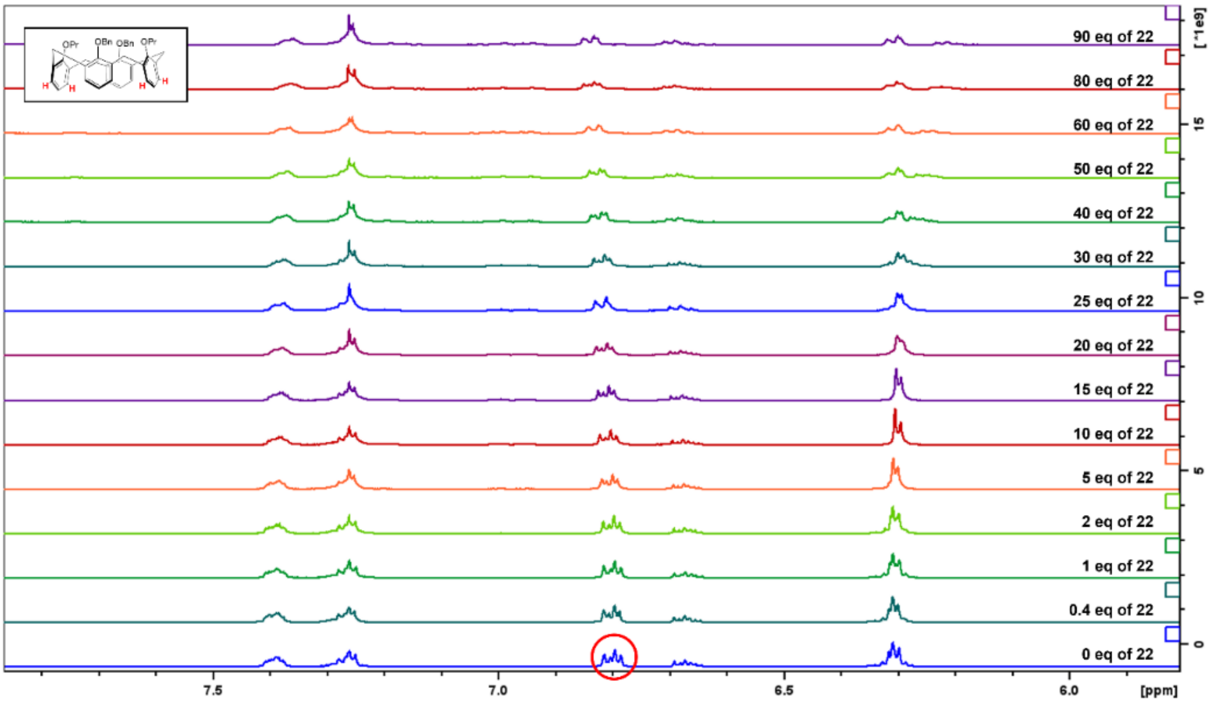

Fig. S62 – Aromatic region of the ^{1}H NMR spectra (9:1 $CDCl_3$:CD3CN) of **13** at various concentrations of **12**

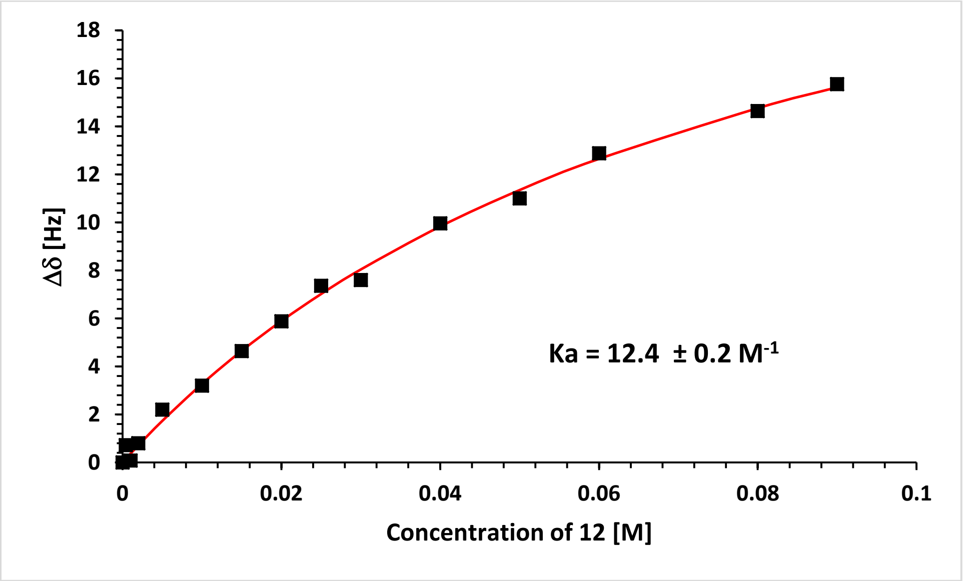

Fig. S63 – Calculation of the Ka for **13**.

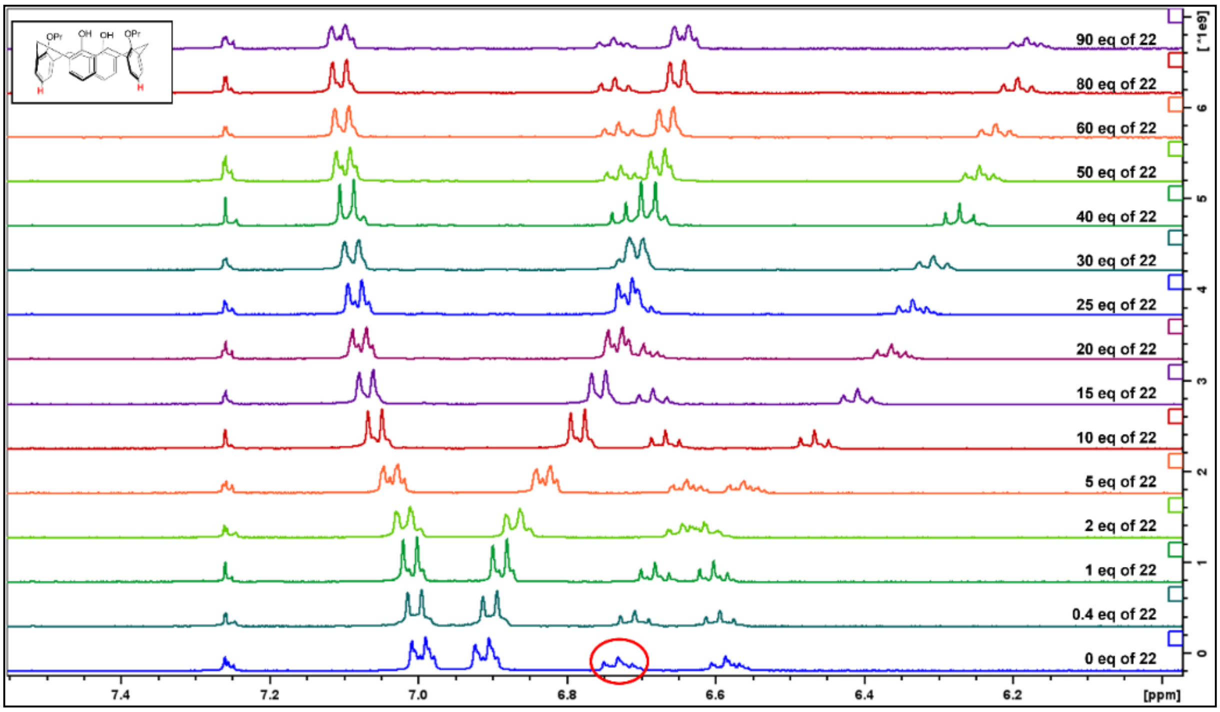

Fig. S64 – Aromatic region of the ^{1}H NMR spectra (9:1 $CDCl_3$:CD3CN) of **14** at various concentrations of **12**

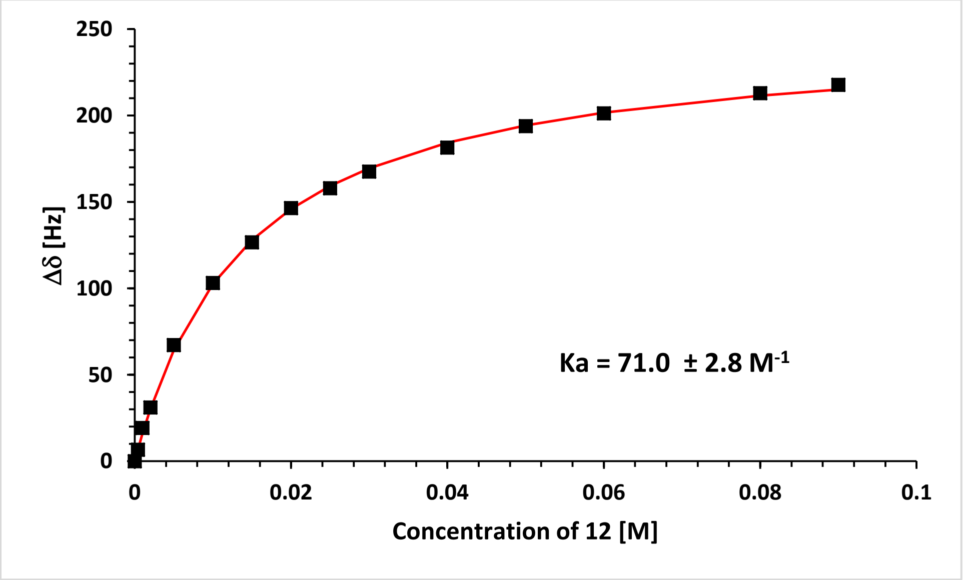

Fig. S65 – Calculation of the Ka for **14**.

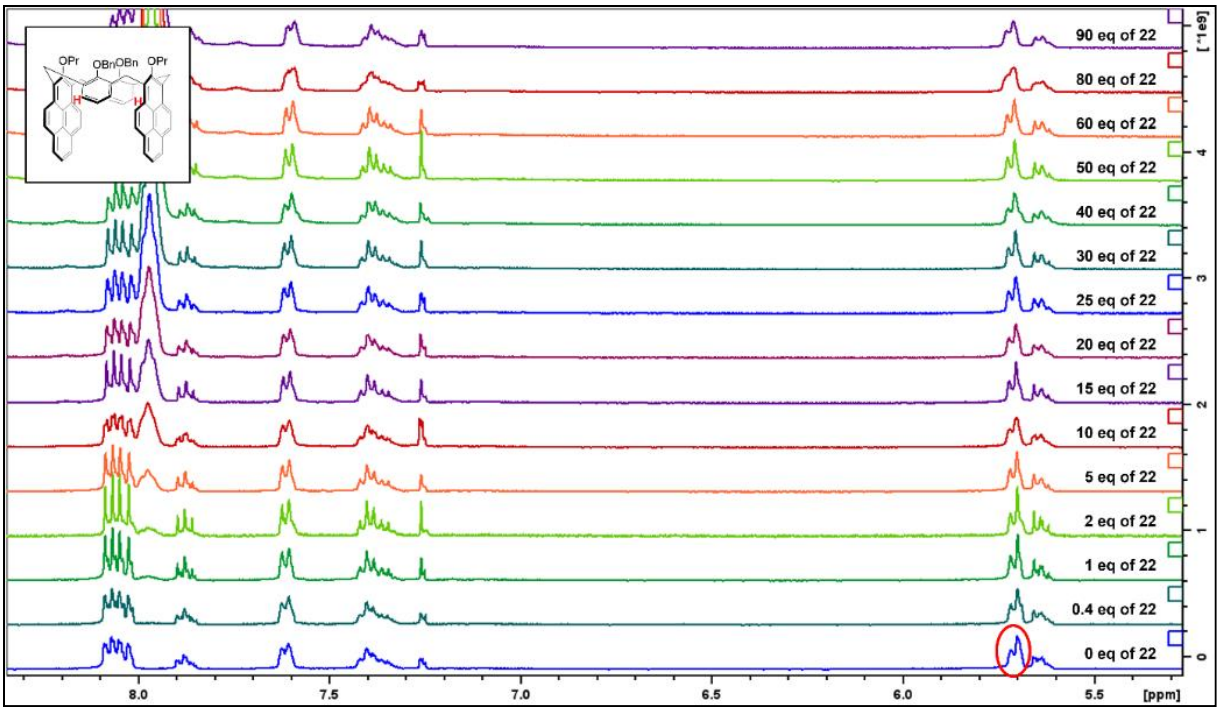

Fig. S66 – Aromatic region of the ^{1}H NMR spectra (9:1 $CDCl_3$:CD3CN) of **11** at various concentrations of **12**

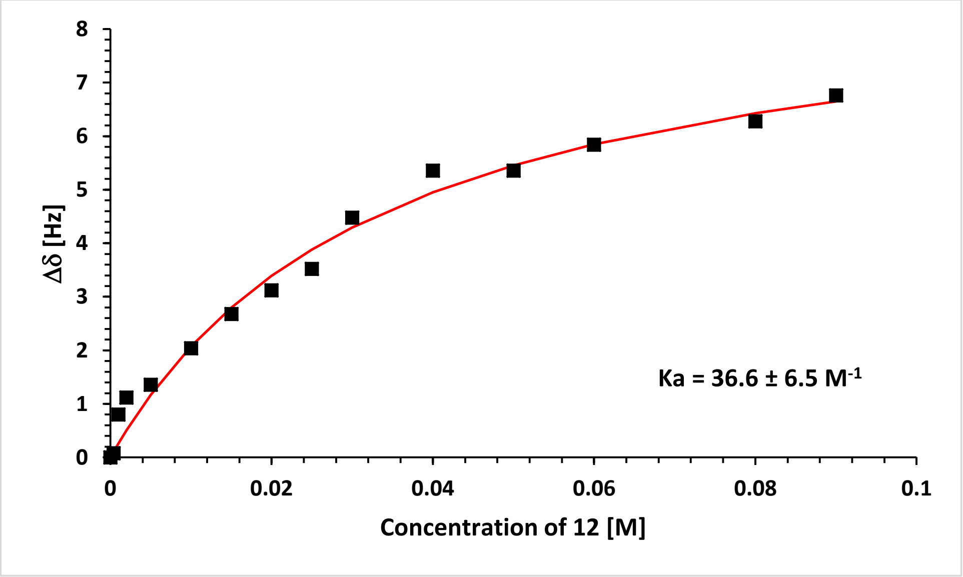

Fig. S67 – Calculation of the Ka for **11**.

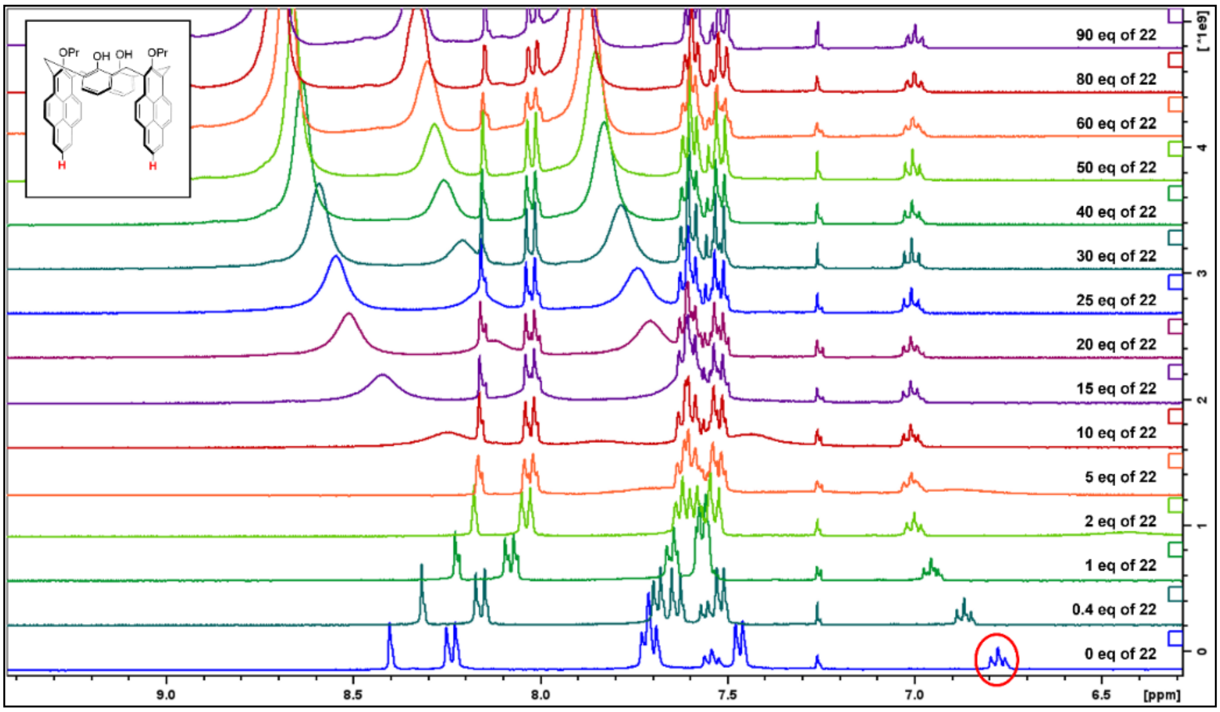

Fig. S68 – Aromatic region of the ^{1}H NMR spectra (9:1 $CDCl_3$:CD3CN) of **2** at various concentrations of **12**

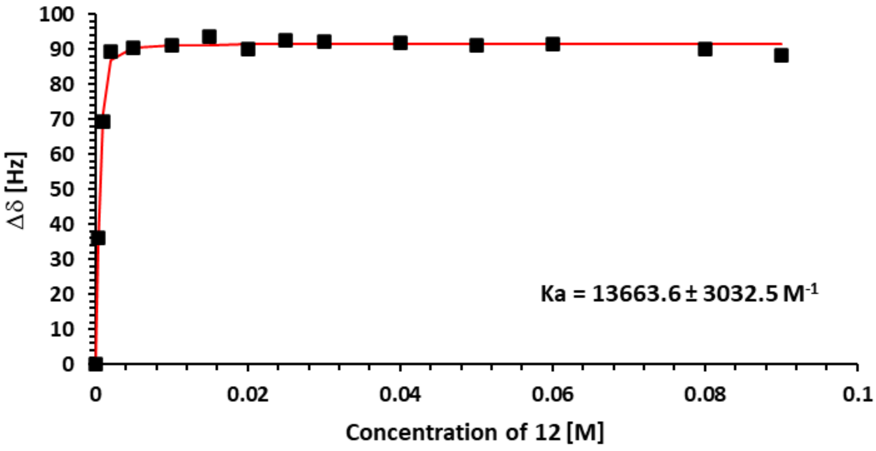

Fig. S69 – Calculation of the Ka for **2**.

5.2 Fluorescence experiments

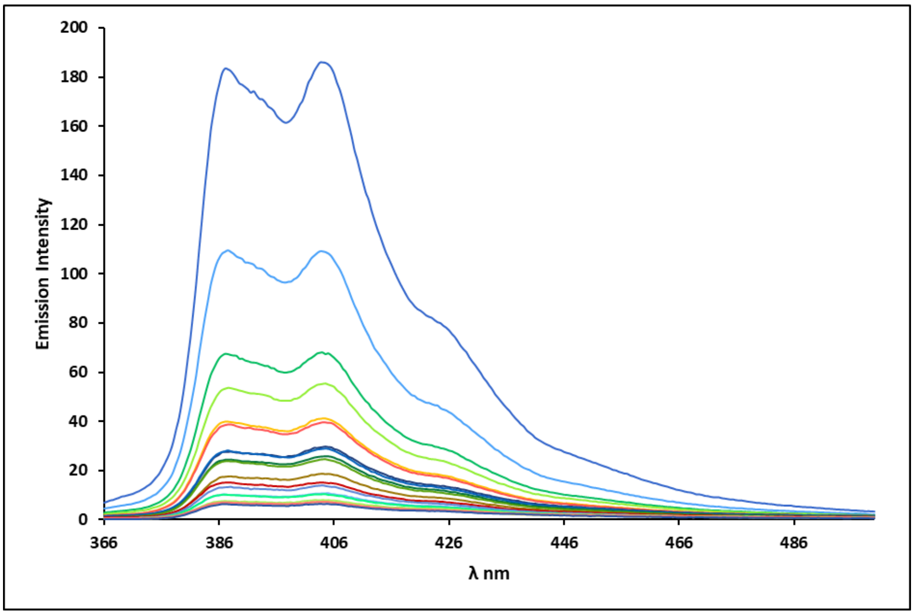

Fig. S70 – Emission changes of **2** (10 µM in $CHCl_3$-CH_3CN, 9:1) upon titration with **12** (1-50 eq.)

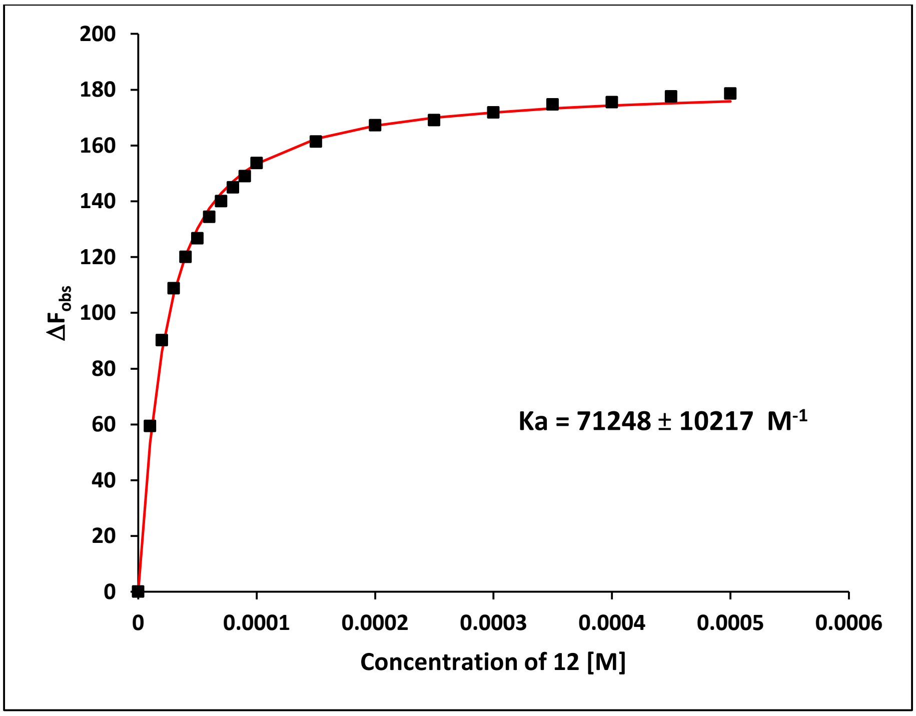

Fig. S71 – Calculation of Ka for **2**.

6. X-ray data collection and structure refinement

Single crystals of complexes CCDC 2346334-2346337 were obtained by slow evaporation of their chloroform solutions. All crystals were coated in oil and flash-frozen in a liquid nitrogen stream. Data were collected at 100 K structures **1** and **2** and 200K for structures **11** and **12⊂2**. Data were collected on a Rigaku Synergy-R diffractometer equipped with a HyPix ARC 150° detector and CuKα (λ=1.54184Å). Data were processed with CrysAlisPRO and structures were solved with SHELXT.[12] All non-hydrogen atoms were further refined by SHELXL[13] with anisotropic displacement coefficients. Hydrogens were placed in calculated positions and refined in a riding mode. Refinement was carried out with the OLEX-2[14] GUI.

Pictures were done with CrystalMaker.[15]

Table S1. Crystallographic data.

Species	1	2	11	12⊂2
**CCDC No.**	2346334	2346335	2346336	2346337
**Formula***	$2C_{41}H_{34}O_4$+$3CH_2Cl_2$	$C_{54}H_{44}O_4$	$C_{68}H_{56}O_4$+H_2O	$C_{54}H_{44}O_4$+C_6H_8N+CF_3O_3S+H_2O
**Molecular weight**	1436.4	756.89	955.14	1018.11
**Crystal system**	Triclinic	Triclinic	Triclinic	Triclinic
**Space group**	*P-1*	*P-1*	*P-1*	*P-1*
**Crystal size (mm)**	0.195×0.083×0.049	0.403×0.105×0.031	0.497×0.131×0.049	0.158×0.104×0.059
**Crystal color and shape**	Colorless Prism	Colorless Plate	Colorless Plate	Colorless Plate
**Temperature (K)**	100	100	200	200
**Wavelength (Å)**	1.54184	1.54184	1.54184	1.54184
**a (Å)**	8.9885(2)	11.6523(2)	11.5529(1)	11.95977(17)
**b (Å)**	13.7934(3)	12.0474(2)	19.2225(2)	12.36365(15)
**c (Å)**	14.3376(4)	15.9369(3)	24.2393(3)	18.01333(19)
**α (º)**	83.408(2)	74.243(2)	79.699(1)	89.2715(9)
**β (º)**	88.665(2)	76.250(2)	78.270(1)	75.6586(11)
**γ (º)**	77.049(2)	65.427(2)	83.875(1)	70.3047(12)
**Volume ($Å^3$)**	1720.93(7)	1938.15(7)	5171.75(10)	2422.27(6)
**Z**	1	2	4	2
**r_{calcd} ($g·cm^{-1}$)**	1.386	1.297	1.227	1.396
**μ (mm^{-1})**	2.764	0.629	0.593	1.205
**No. of reflections (unique)**	79925 (6938)	115596(7773)	209753(20120)	122245 (9764)
**R_{int}**	0.0465	0.0514	0.0545	0.0398
**Completeness to θ (%)**	99.4	99.2	98.9	99.3
**θ max**	75.108	74.445	74.013	75.010
**Data / restraints / parameters**	6938 / 0 / 469	7773 / 0 / 543	20120 / 81 / 1328	9764 / 266 / 788
**Goodness-of-fit on F^2**	1.088	1.044	1.040	1.044
**Final R_1 and wR_2 indices**	0.0471, 0.1342	0.0529, 0.1545	0.0793, 0.2424	0.0455, 0.1301

**[*I*>2s(*I*)]**				
**R_1 and wR_2 indices (all data)**	0.0541, 0.1404	0.0637, 0.1677	0.1129, 0.2906	0.0579, 0.1398
Largest electron density peak and largest hole	0.440 and -0.520	0.409 and -0.255	0.508 and -0.231	0.274 and -0.313

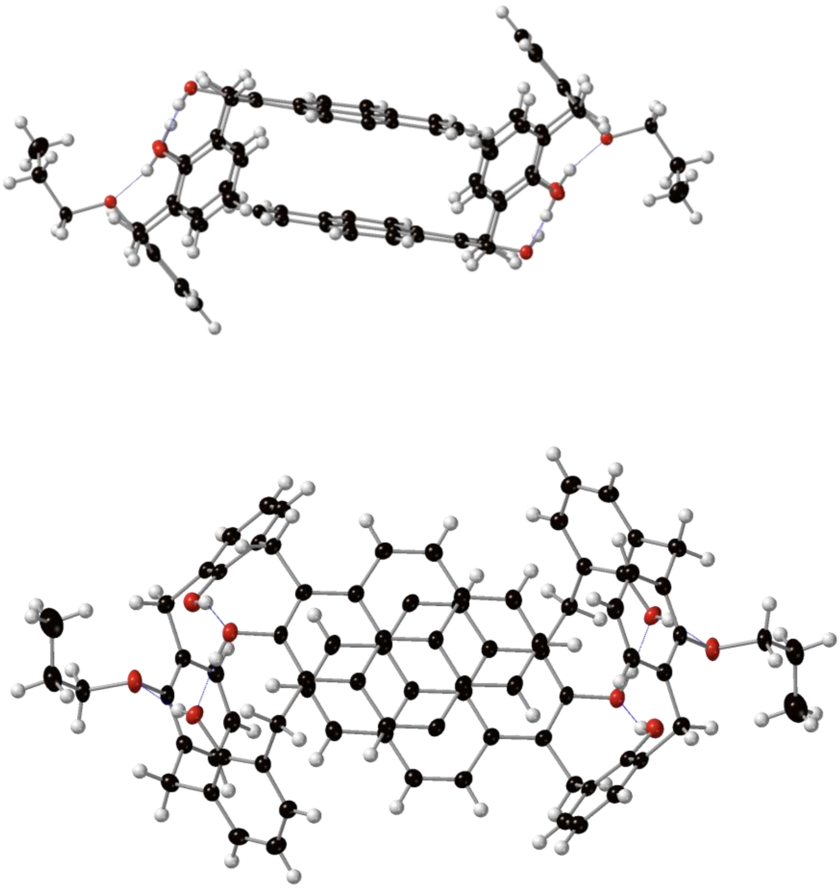

Fig. S72. ORTEP presentation of **1.** Two molecules in the asymmetric unit cell forming a dimer by π-π stacking. Top-side view, Bottom- top view. The thermal ellipsoids are presented in a probability level of 40%. Hydrogens are presented as spheres. Hydrogen bonds are thin dashed blue lines. The DCM molecules were omitted for clarity (C-black, O-red, H-white)

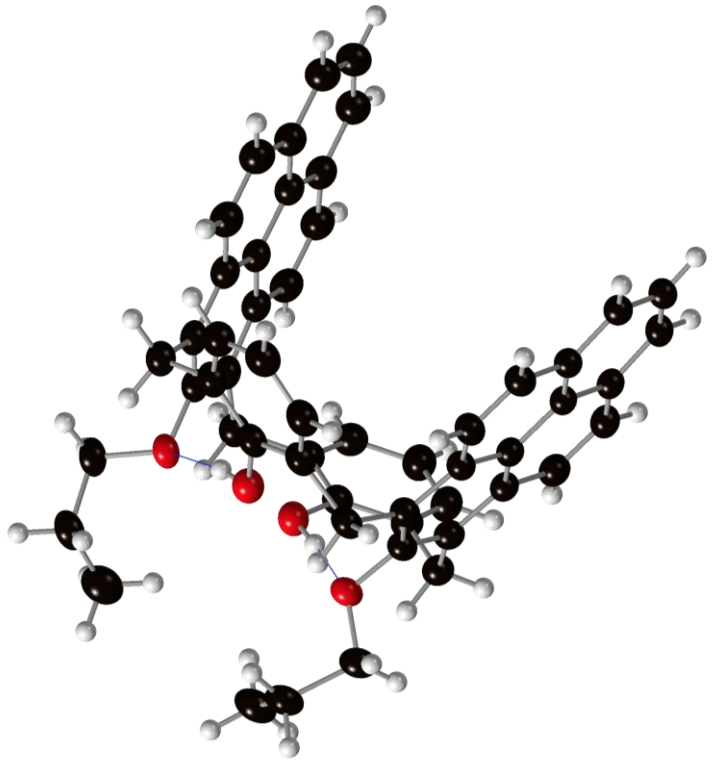

Fig. S73. ORTEP presentation of **2.** The thermal ellipsoids are presented in a probability level of 40%. Hydrogens are presented as spheres. Hydrogen bonds are thin dashed blue lines. (C-black, O-red, H-white)

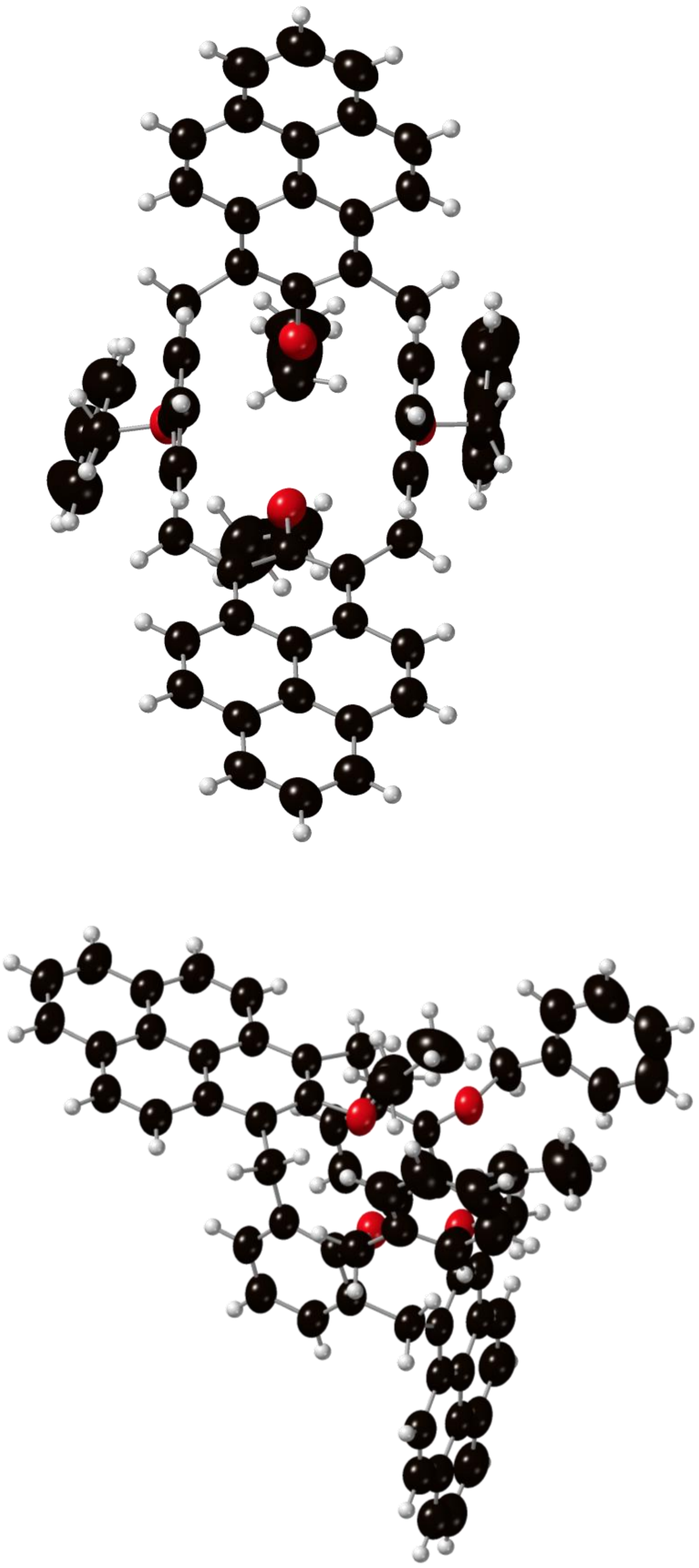

Fig. S74. ORTEP presentation of **11.** The thermal ellipsoids are presented in a probability level of 40%. Hydrogens are presented as spheres. Hydrogen bonds are thin dashed blue lines. One water molecule is omitted. Top -bottom view, bottom -side view (C-black, O-red, H-white)

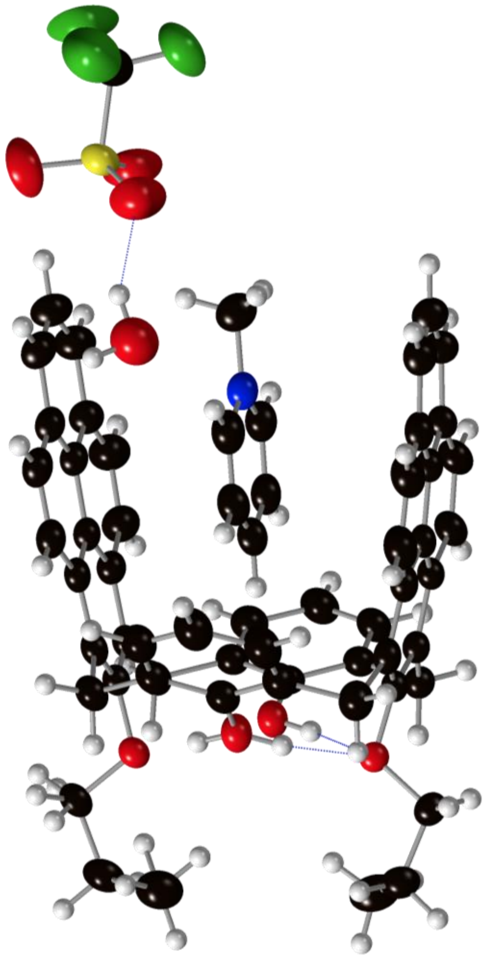

Fig. S75. ORTEP presentation of **12⊂2.** The thermal ellipsoids are presented in a probability level of 40%. Hydrogens are presented as spheres. Hydrogen bonds are thin dashed blue lines. (C-black, O-red, N-blue, S-Yellow, F- green and H-white)

7. References

(1) Thordarson, P.; Coumans, R. G. E.; Elemans, J. A. A. W.; Thomassen, P. J.; Visser, J.; Rowan, A. E.; Nolte, R. J. M. Allosterically Driven Multicomponent Assembly. *Angew. Chem. Int. Ed.* **2004**, *43*, 4755–4759. https://doi.org/10.1002/anie.200460398.

(2) Thordarson, P. Determining Association Constants from Titration Experiments in Supramolecular Chemistry. *Chem. Soc. Rev.* **2011**, *40*, 1305–1323. https://doi.org/10.1039/c0cs00062k.

(3) Rawat, V.; Baheti, A.; Tiwari, O. S.; Vigalok, A. Carbazole-Fused Calixarene Cavities: Single and Mixed AIEgen Systems for NO Detection. *Chem. Commun.* **2023**, *59*, 5543–5546. https://doi.org/10.1039/d3cc01181j.

(4) Zhang, Y. Z.; Xu, M. M.; Si, X. G.; Hou, J. L.; Cai, Q. Enantioselective Synthesis of Inherently Chiral Calix[4]Arenes via Palladium-Catalyzed Asymmetric Intramolecular C-H Arylations. *J. Am. Chem. Soc.* **2022**, *144*, 22858–22864. https://doi.org/10.1021/jacs.2c10606.

(5) Šturala, J.; Boháčová, S.; Chudoba, J.; Metelková, R.; Cibulka, R. Electron-Deficient Heteroarenium Salts: An Organocatalytic Tool for Activation of Hydrogen Peroxide in Oxidations. *J. Org. Chem.* **2015**, *80*, 2676–2699. https://doi.org/10.1021/jo502865f.

(6) Schlüter, D.; Korsching, K. R.; Azov, V. A. Lower-Rim-Modified Calix[4]Arene-Pyrrolotetrathiafulvalene Molecular Tweezers. *Eur. J. Org. Chem.* **2021**, *2021*, 4469–4476. https://doi.org/10.1002/ejoc.202100676.

(7) Gaussian 09, Revision D.01, Frisch, M. J., Trucks, G. W., Schlegel, H. B., Scuseria, G. E., Robb, M. A., Cheeseman, J. R., Scalmani, G., Barone, V., Mennucci, B., Petersson, G. A., Nakatsuji, H., Caricato, M., Li, X., Hratchian, H. P., Izmaylov, A. F., Bloino, J., Zheng, G., Sonnengerg, J. L., Hada, M., Ehara, M., Toyota, K., Fukuda, R., Hasegawa, J., Ishida, Nakajima, T., Honda, Y., Kitao, O., Nakai, H., Vreven, T., Montgomery Jr, J. A., Peralta, J. E., Ogliaro, F., Bearpark, M., Heyd, J. J., Brothers, E., Kudin, K. N., Staroverov, V. N., Keith, T., Kobayashi, R., Normand, J., Raghavachari, K., Rendell, A., Burant, J. C., Iyengar, S. S., Tomasi, J., Cossi, M., Rega, N., Millam, N. J., Klene, M., Knox, J. E., Cross, J. B., Bakken, V., Adamo, C., Jaramillo, J., Gomperts, R., Stratmann, R. E., Yazyev, O., Austin, A. J., Cammi, R., Pomelli, C., Ochterski, J. W., Martin, R. L., Morokuma, K., Zakrzewski, V. G., Voth, G. A., Salvador, P., Dannenberg, J. J., Dapprich, S., Daniels, A. D., Farkas, Ö., Foresman, J. B., Ortiz, J. V., Cioslowski, J. & Fox, D. J. Gaussian, Inc., Wallingford CT, (2010).

(8) a) Perdew, J. P. Density-Functional Approximation for the Correlation Energy of the Inhomogeneous Electron Gas. *Phys. Rev. B* **1986**, *33*, 8822–8824; b) Grimme, S.; Steinmetz, M. Effects of London Dispersion Correction in Density Functional Theory on the Structures of Organic Molecules in the Gas Phase. *Phys. Chem. Chem. Phys*. **2013**, *15*, 16031–16042.

(9) Wu, Y.; Li, M.; Sun, J.; Zheng, G.; Zhang, Q. Synthesis of Axially Chiral Aldehydes by N-Heterocyclic-Carbene-Catalyzed Desymmetrization Followed by Kinetic Resolution. *Angew. Chem. Int. Ed.* **2022**, *61*, e202117340. https://doi.org/10.1002/ANIE.202117340.

(10) Grosjean, S.; Hassan, Z.; Wöll, C.; Bräse, S. Diverse Multi-Functionalized Oligoarenes and Heteroarenes for Porous Crystalline Materials. *Eur. J. Org. Chem.* **2019**, *2019*, 1446–1460. https://doi.org/10.1002/EJOC.201801232.

(11) Zhao, K.; He, Y.; Shan, C.; Wojtas, L.; Ren, J.; Yan, Y.; Shi, H.; Wang, H.; Song, Z.; Shi, X. Construction of Stable Helical Metal-Organic Frameworks with a Conformationally Rigid "Concave Ligand." *Chem. Eur. J.* **2021**, *27*, 10833–10838. https://doi.org/10.1002/CHEM.202101173.

(12) Sheldrick, G. M. SHELXT – Integrated Space-Group and Crystal-Structure Determination *Acta Crystallogr. Sect. A* **2015**, *71*, 3-8.

(13) Sheldrick, G. M. A Short History of SHELX. *Acta Crystallogr. Sect. A* **2008**, *64*, 112–122.

(14) Dolomanov, O. V., Bourhis, L. J., Gildea, R. J., Howard, J. A. K., and Puschmann, H. OLEX2: a Complete Structure Solution, Refinement and Analysis Program. *J. Appl. Crystallogr.* **2009**, *42*, 339–341.

(15) CrystalMaker-11.
